# From the bottle: simple iron salts for the efficient synthesis of pyrrolidines *via* catalytic C–H bond amination†

**DOI:** 10.1039/d2cy02065c

**Published:** 2023-01-25

**Authors:** Wowa Stroek, Lilian Hoareau, Martin Albrecht

**Affiliations:** a Department of Chemistry, Biochemistry and Pharmaceutical Sciences, University of Bern Freiestrasse 3 CH-3012 Bern Switzerland martin.albrecht@unibe.ch

## Abstract

Commercially available iron salts FeX_2_ are remarkably active catalysts for pyrrolidine formation from organic azides *via* direct C–H bond amination. With FeI_2_, amination is fast and selective, (<30 min for 80% yield at 2 mol% loading), TONs up to 370 are reached with just 0.1 mol% catalyst, different functional groups are tolerated, and a variety of C–H bonds were activated.

## Abstract 2

FeI_2_ is a simple and easily accessible yet highly efficient catalyst for C–H bond amination using organic azides, thus making this reaction accessible also without the use of catalysts with sophisticated ligands.

## Graphical abstract

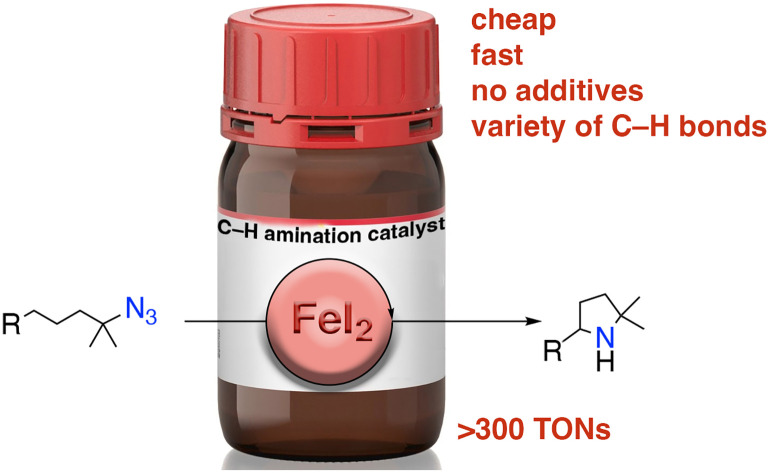

Since its discovery by Betley in 2013, intramolecular C–H amination using organic azides has become an attractive method for the synthesis of N-heterocycles.^1^ This method allows the key step of the C–N bond formation to be highly atom economical, contrary to classical methods such as cross-coupling reactions, reductive elimination, and substitution reactions.^2–6^ Following this pioneering work several other transition metal catalysts have been developed in the recent past for the intramolecular C–H amination with organic azides, including systems based on iron,^1,7–16^ cobalt,^17–21^ nickel,^22,23^ ruthenium^24,25^ and palladium.^26,27^

Iron-based catalysts are particularly active, and are of course also the most attractive due to the high abundance, low toxicity and low costs of the metal.^28^ Although they require Boc_2_O as additive to suppress product inhibition (Fig. 1).^7^ While synthetically convenient, this addition significantly decreases the sustainability and atom-economy. Recently, we demonstrated that a mesoionic carbene ligand suppresses this product inhibition, providing a process that eliminates the necessity for product protection and that runs highly robustly with 7600 turnover numbers (TONs).^14^ Despite these improvements, broad usability has been limited, in parts, by the multistep synthesis required to access the catalysts precursor.

**Fig. 1:**
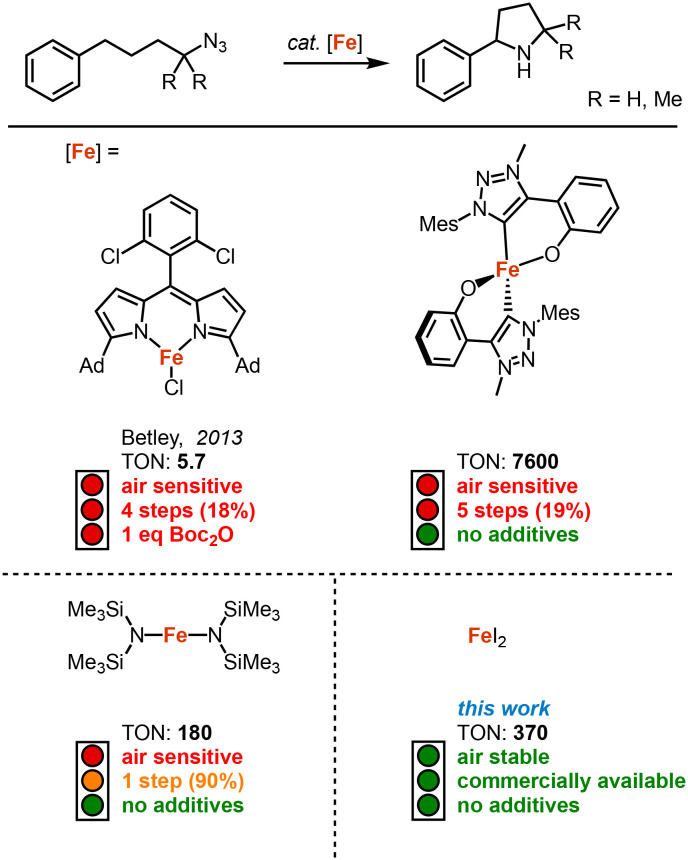
Selected examples of previously reported iron-based catalysts for the catalytic intramolecular C–H amination. The number of steps for the synthesis of the catalyst is represented with its total yield.

The synthetic issue has been partially addressed with the development of Fe(HMDS)_2_ as catalyst,^8^ which is synthesized in just one step from commercial sources (Fig. 1). We became particularly intrigued by blank measurements using commercially available FeCl_2_, which revealed a mediocre 9% yield of pyrrolidine at 1 mol% loading.^14^ Even though this yield is far from synthetically useful, it revealed catalytic activity that, in fact, is similar to that of Betley's pioneering Fe dipyrrin complex (Fig. 1; 9 *vs.* 5.7 TON). Based on these observations, we here report on FeX_2_ salts as convenient, commercially available, and air-stable catalyst precursors for the intramolecular C–H bond amination. We have benchmarked these salts in the preparation of different pyrrolidines, revealing high yields from organic azides without the need for any additives. This method therefore provides a remarkably sustainable and facile C–N bond formation protocol.

Our previous results showed that the model reaction of (4-azido-4-methylpentyl)benzene (1a) with 1 mol% FeCl_2_ resulted in a 9% yield of pyrrolidine 1b after 6 h at 120 °C (Table 1, entry 1).^14^ To optimize this process, the catalysis was run with an increased FeCl_2_ loading (10 mol%), which improved the yield of the amine product to 49% (entry 2). Moreover, significant amounts of the imine side product 1c were formed (21%), which was characterized by a distinct multiplet in the ^1^H NMR spectrum at 7.75 ppm for the aryl proton in *para*-position. Also, trace amounts of enamine 1d were observed, characterized by the allylic signal at 5.19 ppm. The overall yield for C–H bond activation is therefore around 70%. Notably, the tetrahydrate version of FeCl_2_, gave a much lower 8% yield, suggesting that H_2_O is detrimental to catalytic activity (entry 3). Based on the significant catalytic activity of FeCl_2_ at 10 mol% loading, a variety of iron salts were screened as catalyst precursors under these conditions. FeBr_2_ showed activity and selectivity similar to FeCl_2_ (entry 4). Interestingly, when exposing the reaction mixture to air with addition of pentane, colorless crystals suitable for X-ray analysis were obtained. The crystals were composed of the protonated amine 1b with a bromide counterion, 1b**.HBr** (Fig. 2). We hypothesize that HBr is formed upon exposing the reactive iron catalyst to air, and subsequently trapped by the pyrrolidine 1b. Using FeI_2_ instead of FeCl_2_ increased the yield of the amine substantially to 74% and lowered the amounts of imine and enamine by-products (Table 1, entry 5). In contrast, the iron salts Fe(OAc)_2_, Fe(OTf)_2_, Fe(BF_4_)_2_·6H_2_O and FeSO_4_·7H_2_O did not show any detectable activity in C–H amination catalysis (entries 6–9).

**Table 1:** Use of different commercially available iron salts for the intramolecular C–H amination^a^

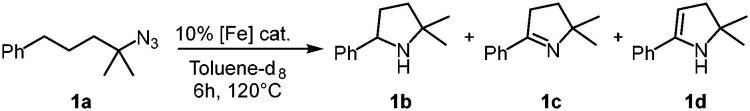						
Entry	Catalyst	Mol%	Yield^b^ (%)			
			1b	1c	1d	Total
1 (ref. 14)	FeCl_2_	1	9	<2	n.d.	9
2	FeCl_2_	10	49	21	<2	70
3	FeCl_2_·4H_2_O	10	8	<2	<2	8
4	FeBr_2_	10	48	17	<2	65
5	FeI_2_	10	74	6	<2	80
6	Fe(OAc)_2_	10	n.d.	n.d.	n.d.	0
7	Fe(OTf)_2_	10	n.d.	n.d.	n.d.	0
8	Fe(BF_4_)_2_·6H_2_O	10	n.d.	n.d.	n.d.	0
9	FeSO_4_·7H_2_O	10	n.d.	n.d.	n.d.	0

a Catalysis was performed on a 0.25 mmol scale in J Young NMR tubes; see ESI† for exact experimental details.

b Yields and conversions were determined by ^1^H NMR spectroscopy using 1,3,5-trimethoxybenzene as internal standard.

**Fig. 2:**
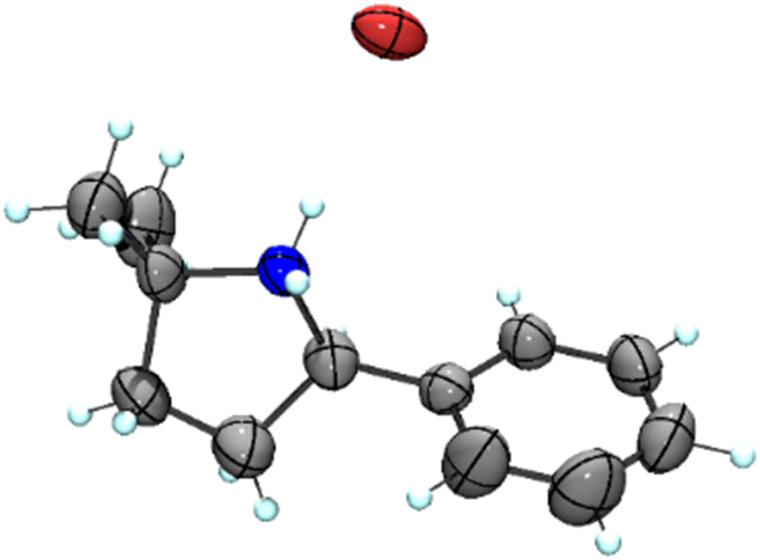
Molecular structure from X-ray diffraction analysis of protonated amine 1b**.HBr** (50% probability ellipsoids).

Further catalytic optimization entailed the variation of the solvent (Table S1†), and, in particular, the FeI_2_ loading between 0.1 and 50 mol%. These experiments revealed a remarkable trend, as increasing the catalyst loading decreased the product yield (Table 2). Using 30–50 mol% of catalyst resulted in full substrate conversion but only (sub)stoichiometric amounts of product (Table 2, entries 1–3). Upon lowering the catalyst loading to the 2–20 mol% range, full conversion is maintained, paired with a gradual increase in product yield from 52% to 83% as catalyst loading is lowered (entries 4–7). We attribute this unusual correlation of catalyst loading and product yield to the binding of either the azide substrate or, more likely, the product to the paramagnetic iron species. Such binding idles this fraction for ^1^H NMR integration and has also been observed with Fe(HMDS)_2_.^8^ This model rationalizes the higher yields of cyclic amine observed at lower catalyst loadings. Further lowering of the catalyst loading to 0.5 and 0.1 mol% did not result in full conversion anymore and gave yields of 70% and 27%, respectively (entries 8–9). Extending the reaction time to 24 h with 0.1 mol% FeI_2_ increased the yield to 37%, yet a further increase of reaction time to 7 days did not improve the yield any further (entry 10). The maximum turnover number therefore is 370 under these conditions.

**Table 2:** Effect of different loadings of FeI_2_ on the catalytic intramolecular C–H amination

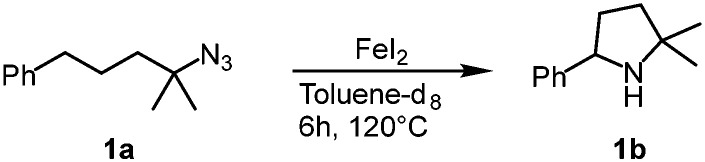			
Entry^a^	FeI_2_ (mol%)	Yield 1b^b^ (%)	Conversion^b^ (%)
1	50	8	98
2	40	18	98
3	30	33	98
4	20	52	98
5	10	74	98
6	5	81	96
7	2	83	97
8	0.5	70	86
9	0.1	27	29
10^c^	0.1	37	49

a Catalysis was performed on a 0.25 mmol scale in J Young NMR tubes; see ESI† for exact experimental details.

b Yields and conversions were determined by ^1^H NMR spectroscopy using 1,3,5-trimethoxybenzene as internal standard.

c Reaction time was increased to 24 h.

To get more insight into the catalytic activity of FeI_2_ at low catalyst loading, the reaction was followed by ^1^H NMR spectroscopy. After heating at 120 °C for 5 min using 5 mol% FeI_2_, the yield was 26%, corresponding to an initial turnover frequency (TOF) of at least 60 h^−1^ (Fig. S2†). In comparison, the highest TOF observed for this transformation is 150 h^−1^ using 1 mol% of a mesoionic carbene iron complex.^14^ The catalytic rate then gradually slows down as expected for a first order reaction. Mechanistically, FeI_2_ may operate similar to other iron catalysts *via* imidyl/nitrene formation and subsequent hydrogen atom abstraction and sequential or concerted C–N bond formation.^8,20–22^ In agreement with the involvement of an open-shell species, conversion was completely stalled when TEMPO was added to a catalytic run at early reaction stages (14% conversion). Though other scenarios like coordination by TEMPO or iron oxidation obviously may also cause this effect.

To benchmark FeI_2_ as a C–H amination catalyst against known iron catalysts, a set of standard organic azide substrates with diverse substitution patterns were evaluated (Fig. 3). Primary azides are not suitable substrates as azide 2a did not form any detectable pyrrolidine and instead, a mixture of unidentified products was obtained. Primary azides are known to be challenging substrates, as the protons in the α-position can undergo a hydrogen atom abstraction (HAA) upon formation of the nitrene.^29^ This limitation has been observed with some, but not all,^1,7,9–11,16^ previously developed Fe catalysts.^14,22^

**Fig. 3:**
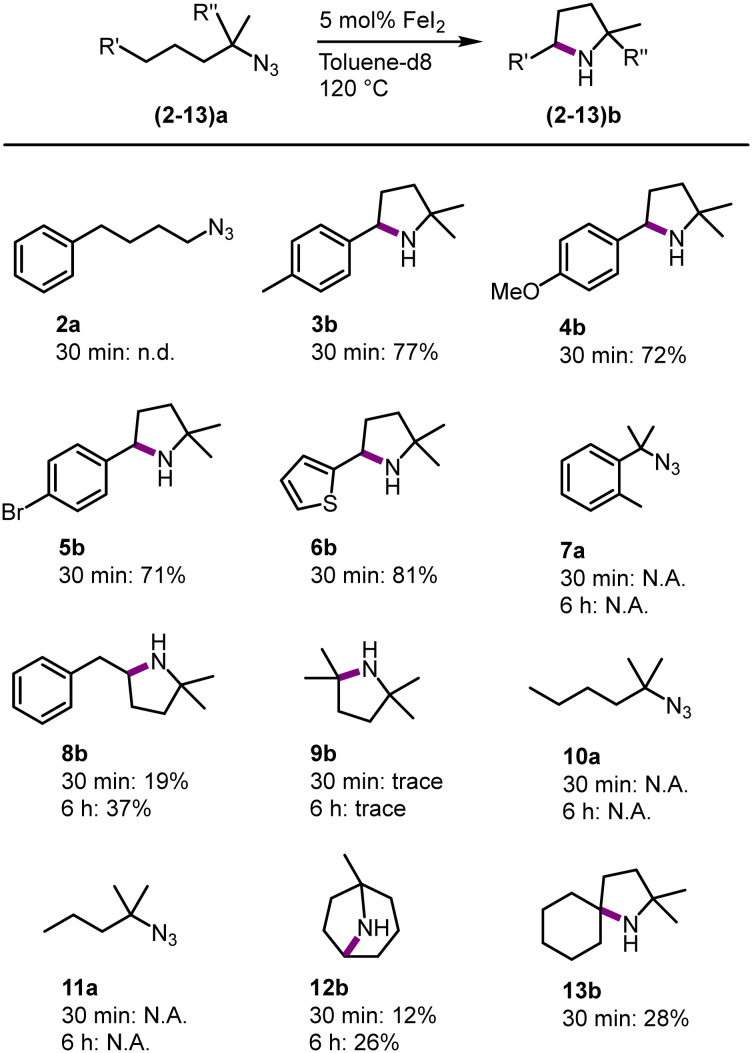
Substrate scope of the intramolecular C–H amination using 5 mol% of FeI_2_ in toluene-d_8_ at 120 °C, spectroscopic yields determined by ^1^H NMR spectroscopy using 1,3,5-trimethoxybenzene as internal standard.

In contrast, FeI_2_ catalyzes the C–H amination with a variety of tertiary azides. Substituting the *para*-position of the model substrate by a Me, OMe, Br or a thiophene group resulted in a 70–81% yield of the cyclic amine (3b–6b) after 30 min. The activity is similar to 1b and suggests only a minor influence of aromatic substituents on the amination reactivity. Activation of a primary rather than secondary benzylic C–H bond as in substrate 7a did not lead to any detectable amount of the corresponding indolidine. Instead, mainly starting material was observed, with small unidentified impurities, even after prolonged reaction times (6 h). This reactivity contrasts with the high conversion of this substrate with iron catalysts that contain chelating ligands.^1,7,8,14^ Expanding the alkyl chain by one methylene group (substrate 8a) selectively affords the pyrrolidine 8b (37% after 6 h), while no signals were detected for a piperidine homologue that would result from benzylic C–H bond activation. This result indicates that the reaction is kinetically controlled to give the 5-membered heterocycle, rather than producing the less ring-strained 6-membered piperidine from activation of the weaker benzylic C–H bond.^30^ This selectivity is identical to that of other homogeneous iron catalysts containing robustly bound ligands,^8,14^ yet distinct from other catalytic systems that tend to afford mixtures of both heterocycles.^31^ Intrigued by the ability of FeI_2_ as a precatalyst for the activation of the strong aliphatic C–H bond in 8a, other aliphatic substrates were evaluated. The tertiary aliphatic C–H bond in 9a is poorly aminated, producing the aliphatic pyrrolidine 9b only in trace amounts even after longer reaction times. Secondary and primary aliphatic C–H bonds are unreactive and 10a and 11a did not lead to any cyclized product. Remarkably, tertiary and even secondary aliphatic C–H bonds are activated when using substrates 12a and 13a, respectively. While these C–H bond strengths are comparable to those in 9a and 10a, respectively, the amine product is sterically more protected. This distinct reactivity pattern may point to product inhibition, which is expected to be more relevant with less sterically shielded amines and with catalysts containing small and kinetically labile ligands. Indeed, the introduction of bidentate mesoionic carbene ligands at the iron center efficiently suppresses such inhibition.^14^ Mimicking this effect by adding exogeneous ligands such as pyridine, bipyridine, or PPh_3_ did not improve conversion, or even suppressed activity completely (bi- or terpyridine; Table S2†). Overall, FeI_2_ has considerable limitations as a catalyst for the amination of unshielded aliphatic C–H bonds, though it offers an attractive alternative to sophisticated iron complexes for the amination of benzylic C–H bonds as well aliphatic C–H bonds that are producing sterically rather shielded amine products. It may therefore become a viable methodology for the construction of the pyrrolidine motif in agrochemical and pharmaceutical products with this motif.^32–36^

In conclusion, a convenient and sustainable method is described for the synthesis of pyrrolidines from organic azides through direct C–H amination using FeI_2_ as a cheap, commercially available, and air-stable catalyst. Low catalyst loadings accomplish the synthesis of these N-heterocycles in high yields with low reaction times. Some functional groups are tolerated by this catalyst, and activation of benzylic C–H bonds is considerably more efficient than activation of stronger aliphatic C–H bonds. The latter requires sterically congested pyrrolidine products to be formed in order to limit product inhibition. The convenience of an air-stable and commercial catalyst is attractive for implementing C–H amination as a method for pyrrolidine synthesis both for organic synthesis and industrial applications.

## Author contributions

W. S. and L. H. performed the work and analysed the data. W. S. and M. A. conceived the project and wrote the manuscript. M. A. supervised the project.

## Conflicts of interest

There are no conflicts to declare.

## Supplementary Material

CY-013-D2CY02065C-s001

CY-013-D2CY02065C-s002
