# Supplementary material for: From the bottle: simple iron salts for the efficient synthesis of pyrrolidines via catalytic C–H bond amination
Source: Catal Sci Technol. 2023 Jan 25;13(4):958–62. doi: 10.1039/d2cy02065c (PMC9939938; doi:10.1039/d2cy02065c)
Supplement: CY-013-D2CY02065C-s001 [file CY-013-D2CY02065C-s001.pdf]

Electronic Supplementary Material (ESI) for Catalysis Science & Technology.
This journal is © The Royal Society of Chemistry 2023

Electronic Supporting Information

From the Bottle: Simple Iron Salts for the Efficient Synthesis of Pyrrolidines via Catalytic C–H Bond Amination

Wowa Stroek[1], Lilian Hoareau[1] and Martin Albrecht[1,*]

[1] Department of Chemistry, Biochemistry and Pharmaceutical Sciences, University of Bern, Freiestrasse 3, CH-3012 Bern, Switzerland

Table of Contents

General consideration **S3**

Substrate synthesis **S4**

Catalysis **S17**

General procedure S17

Catalysis with low catalyst loadings S17

Solvent scope S18

Effect of additives S19

Radical trapping S19

Characterization of C–H aminated products S20

NMR spectra **S23**

Substrate synthesis S23

Catalysis S53

Crystallographic and refinement data **S60**

References **S61**

General considerations

Chemicals, solvents and synthesis

All manipulations involving transition metal complexes were performed inside an argon filled MBraun glovebox with <0.1 O_2 and H_2O levels using dry and degassed solvents, unless stated otherwise. THF-d_8, C_6D_6 and toluene-d_8 were distilled over NaK, degassed by three freeze-pump-thaw cycles and dried over 4 Å molecular sieves. DMF-d_7 and DMSO-d_6 were degassed by three freeze-pump-thaw cycles and dried over 4 Å molecular sieves for a week 4 times. Molecular sieves were pre-dried in a 1000W microwave for 10 minutes, in 30 seconds intervals. After which they were dried under vacuum at 220 °C for 7 days.

All organic synthesis was performed under aerobic conditions with commercially available solvents, unless stated otherwise. All other chemicals were used as received from commercial sources.

NMR-spectroscopy

All ^1H and ^{13}C NMR spectra were recorded on a Bruker AVANCE III HD 300. The chemical shifts are reported relative to $SiMe_4$ using the chemical shift of residual solvent peaks as reference.[S1]

Single crystal X-ray diffraction

A crystal of **1b.HBr** was measured on a RIGAKU Synergy S area-detector diffractometer[S2] using mirror optics monochromated Cu Kα radiation (λ = 1.54184 Å).

Data reduction was performed using the *CrysAlisPro*[S2] program. The intensities were corrected for Lorentz and polarization effects, and an absorption correction based on the multi-scan method using SCALE3 ABSPACK in *CrysAlisPro*[S2] was applied.

The structure was solved by direct methods using *SHELXT*,[S3] which revealed the positions of all non-hydrogen atoms of the title compounds. All non-hydrogen atoms were refined anisotropically. H-atoms were assigned in geometrically calculated positions and refined using a riding model where each H-atom was assigned a fixed isotropic displacement parameter with a value equal to 1.2 Ueq of its parent atom (1.5 Ueq for methyl groups).

Refinement of the structure was carried out on F^2 using full-matrix least-squares procedures, which minimized the function $\Sigma w(F_o^2 - F_c^2)^2$. The weighting scheme was based on counting statistics and included a factor to downweight the intense reflections. All calculations were performed using the *SHELXL-2014/7*[S3] program in OLEX2.[S4] Further crystallographic details are compiled in table S3. Crystallographic data for the structure of **1b.HBr** has been deposited with the Cambridge Crystallographic Data entre (CCDC) as supplementary publication number 2208587.

Substrate synthesis

All the syntheses of substrates **1a**-**13a** have been reported previously,[S14] and included here for the sake of convenience and completion.

General procedure

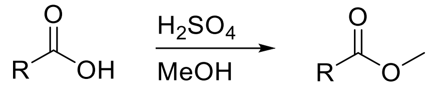

Synthesized according to a literature procedure.[S5] Corresponding carboxylic acid was dissolved in MeOH and 10 drops of concentrated sulphuric acid were added. The solution was stirred for 16 h and concentrated under reduced pressure. Water was added and the emulsion was extracted with Et_2O, washed with brine, dried over Na_2SO_4, filtered and concentrated to obtain the corresponding ester as the product.

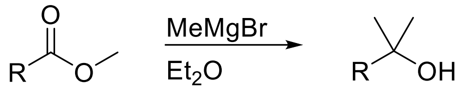

Synthesized according to a literature procedure.[S5] In an oven dried Schlenk under an argon atmosphere corresponding ester (1.0 eq) was dissolved in anhydrous Et_2O and cooled to 0 °C. A solution of 3.0 M MeMgBr (3.0 eq) in Et_2O was added dropwise and the obtained white suspension was stirred for 16 h. The mixture was quenched with concentrated aqueous NH_4Cl solution and extracted with Et_2O, washed with brine, dried over Na_2SO_4, filtered and concentrated to obtain the corresponding alcohol as the product.

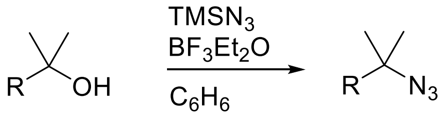

Synthesized according to a literature procedure.[S5] In an oven dried Schlenk under an argon atmosphere corresponding alcohol (1.0 eq) and $TMSN_3$ (1.2 eq) was dissolved in anhydrous C_6H_6. BF_3Et_2O (1.2 eq) was added dropwise and the solution was stirred for 16 h. The obtained mixture was quenched with water, extracted with Et_2O, washed with brine, dried over Na_2SO_4, filtered and concentrated. The crude product was purified by column chromatography over SiO_2 using hexane as eluent.

All azide products were transferred into a J Young Schlenk, degassed by four freeze-pump-thaw cycles and dried over 4 Å molecular sieves for at least one week before use in catalysis.

Substrate **1a**

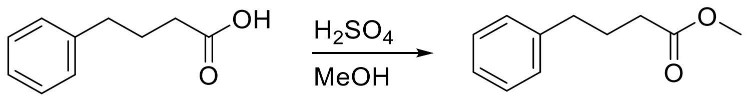

Synthesized according to a literature procedure.[S6] 4-phenylbutanoic acid (50.0 g; 305 mmol; 1.0 eq) was dissolved in MeOH (500 mL) and 10 drops of concentrated sulphuric acid were added. The solution was stirred for 16 h and concentrated under reduced pressure. Water (100 mL) was added and the emulsion was extracted with Et_2O (3x 250 mL), washed with brine (100 mL), dried over Na_2SO_4, filtered and concentrated. The product was obtained as a colorless oil (52.11 g; 292.4 mmol; 96%).

Spectral data were consistent with previously reported characterization of the product.[S6] ^1H NMR (300 MHz, CD_2Cl_2) δ 7.32 – 7.23 (m, 2H), 7.23 – 7.05 (m, 3H), 3.64 (s, 3H), 2.64 (dd, *J* = 8.5, 6.8 Hz, 2H), 2.32 (t, *J* = 7.5 Hz, 2H), 1.93 (p, *J* = 7.5 Hz, 2H).

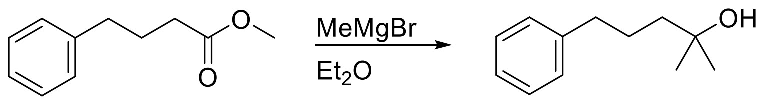

Synthesized according to a literature procedure.[S6] In an oven dried Schlenk under an argon atmosphere methyl 4-phenylbutanoate (52.0 g; 292 mmol; 1.0 eq) was dissolved in anhydrous Et_2O (300 mL) and cooled to 0 °C. A solution of 3.0 M MeMgBr (292 mL; 875 mmol; 3.0 eq) in Et_2O was added dropwise and the obtained white suspension was stirred for 16 h. The mixture was quenched with concentrated aqueous NH_4Cl (200 mL) solution and extracted with Et_2O (5x 250 mL), washed with brine (100 mL), dried over Na_2SO_4, filtered and concentrated. The product was obtained as a colorless oil (38.91 g; 218.3 mmol; 75%).

Spectral data were consistent with previously reported characterization of the product.[S6] ^1H NMR (300 MHz, $CDCl_3$) δ 7.26 – 7.15 (m, 2H), 7.15 – 7.07 (m, 3H), 2.54 (t, *J* = 7.6 Hz, 2H), 1.70 – 1.54 (m, 2H), 1.49 – 1.37 (m, 2H), 1.12 (s, 6H).

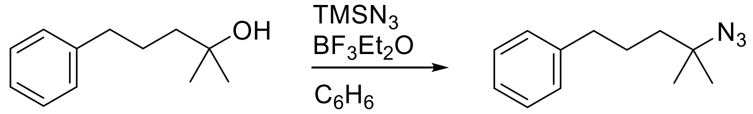

Synthesized according to a literature procedure.[S6] In an oven dried Schlenk under an argon atmosphere 2-methyl-5-phenylpentan-2-ol (30.0 g; 168 mmol; 1.0 eq) and $TMSN_3$ (26.8 mL; 202 mmol; 1.2 eq) was dissolved in anhydrous C_6H_6 (500 mL). BF_3Et_2O (24.9 mL; 202 mmol; 1.2 eq) was added dropwise and the solution was stirred for 16 h. The obtained mixture was quenched with water (200 mL), extracted with Et_2O (3x 250 mL), washed with brine (100 mL), dried over Na_2SO_4, filtered and concentrated. The crude product was purified by column chromatography over SiO_2 using hexane as eluent. The product was obtained as a colorless oil (13.55 g; 66.7 mmol; 40%).

Spectral data were consistent with previously reported characterization of the product.[S6] ^1H NMR (300 MHz, $CDCl_3$) δ 7.42 – 7.31 (m, 2H), 7.31 – 7.20 (m, 3H), 2.70 (t, J = 7.5 Hz, 2H), 1.89 – 1.67 (m, 2H), 1.67 – 1.42 (m, 2H), 1.33 (s, 6H). ^{13}C NMR (75 MHz, $CDCl_3$) δ 142.17, 128.50, 126.01, 61.71, 41.19, 36.18, 26.24, 26.13.

Substrate **2a**

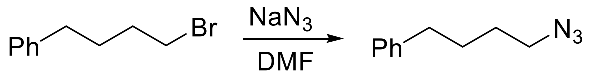

Adjusted from a literature procedure.[S7] (4-bromobutyl)benzene (15.00 g; 12.1 mL; 70.4 mmol; 1.0 eq) and NaN_3 (13.73 g; 211.2 mmol; 3.0 eq) were dissolved in DMF (250 mL) and stirred for 16 hours at 80 °C. The reaction was allowed to cool to room temperature and H_2O (200 mL) was added. The mixture was extracted with Et_2O (3x 150 mL), dried over Na_2SO_4, filtered and concentrated. The crude mixture was purified by flash column chromatography over SiO_2 using hexane as eluent. The product was obtained as a colorless oil (11.39 g; 65.0 mmol; 92%).

Spectral data were consistent with previously reported characterization of the product.[S5] ^1H NMR (300 MHz, $CDCl_3$) δ 7.26 – 7.16 (m, 2H), 7.12 (m, 3H), 3.21 (t, J = 6.5 Hz, 2H), 2.57 (t, J = 7.2 Hz, 2H), 1.75 – 1.50 (m, 4H). ^{13}C NMR (75 MHz, $CDCl_3$) δ 141.97, 128.53, 128.51, 126.05, 51.49, 35.51, 28.59.

Substrate **3a**

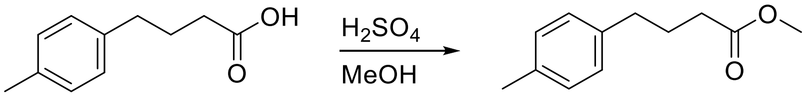

Synthesized according to a literature procedure.[S8] 4-(p-tolyl)butanoic acid (8.00 g; 44.9 mmol; 1.0 eq) was dissolved in MeOH (100 mL) and 10 drops of concentrated sulphuric acid were added. The solution was stirred for 16 h and concentrated under reduced pressure. Water (100 mL) was added and the emulsion was extracted with Et_2O (3x 100 mL), washed with brine (100 mL), dried over Na_2SO_4, filtered and concentrated. The product was obtained as a colorless oil (7.80 g; 40.6 mmol; 90%).

Spectral data were consistent with previously reported characterization of the product.[S8] ^1H NMR (300 MHz, $CDCl_3$) δ 7.05 – 6.92 (m, 4H), 3.57 (s, 3H), 2.52 (t, J = 7.6 Hz, 2H), 2.23 (d, J = 2.7 Hz, 5H), 1.84 (p, J = 7.6 Hz, 2H). ^{13}C NMR (75 MHz, $CDCl_3$) δ 174.13, 138.41, 135.55, 129.19, 128.49, 51.62, 34.81, 33.53, 26.73, 21.12.

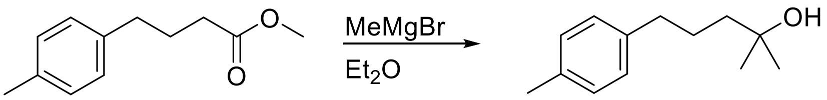

Synthesized according to a literature procedure.[S6] In an oven dried Schlenk under an argon atmosphere methyl 4-(p-tolyl)butanoate (7.79 g; 40.5 mmol; 1.0 eq) was dissolved in anhydrous Et_2O (200 mL) and cooled to 0 °C. A solution of 3.0 M MeMgBr (40.5 mL; 122 mmol; 3.0 eq) in Et_2O was added dropwise and the obtained white suspension was stirred for 16 h. The mixture was quenched with concentrated aqueous NH_4Cl (25 mL) solution and extracted with Et_2O (3x 100 mL), washed with brine (100 mL), dried over Na_2SO_4, filtered and concentrated. The product was obtained as a colorless oil (7.00 g; 36.4 mmol; 90%).

Spectral data were consistent with previously reported characterization of the product.[S6] ^{1}H NMR (300 MHz, $CDCl_3$) δ 7.10 (s, 4H), 2.59 (t, *J* = 7.5 Hz, 2H), 2.33 (s, 3H), 1.77 – 1.60 (m, 2H), 1.57 – 1.45 (m, 2H), 1.31 (s, 1H), 1.21 (s, 6H). ^{13}C NMR (75 MHz, $CDCl_3$) δ 139.47, 135.28, 129.12, 128.40, 71.08, 43.64, 36.01, 29.36, 26.52, 21.12.

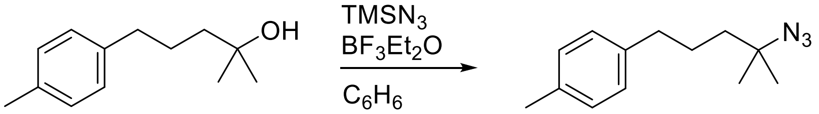

Synthesized according to a literature procedure.[S6] In an oven dried Schlenk under an argon atmosphere 2-methyl-5-(p-tolyl)pentan-2-ol (6.44 g; 33.5 mmol; 1.0 eq) and $TMSN_3$ (5.3 mL; 40.2 mmol; 1.2 eq) was dissolved in anhydrous C_6H_6 (200 mL). BF_3Et_2O (4.96 mL; 40.2 mmol; 1.2 eq) was added dropwise and the solution was stirred for 16 h. The obtained mixture was quenched with water (100 mL), extracted with Et_2O (3x 100 mL), washed with brine (100 mL), dried over Na_2SO_4, filtered and concentrated. The crude product was purified by column chromatography over SiO_2 using hexane as eluent. The product was obtained as a colorless oil (1.77 g; 8.1 mmol; 24%).

Spectral data were consistent with previously reported characterization of the product.[S6] ^{1}H NMR (300 MHz, $CDCl_3$) δ 7.15 – 7.03 (m, 4H), 2.59 (t, *J* = 7.5 Hz, 2H), 2.33 (s, 3H), 1.76 – 1.56 (m, 2H), 1.56 – 1.41 (m, 2H), 1.25 (s, 6H). ^{13}C NMR (75 MHz, $CDCl_3$) δ 139.09, 135.44, 129.19, 128.37, 61.73, 41.20, 35.73, 26.36, 26.12, 21.14.

<u>Substrate 4**a**</u>

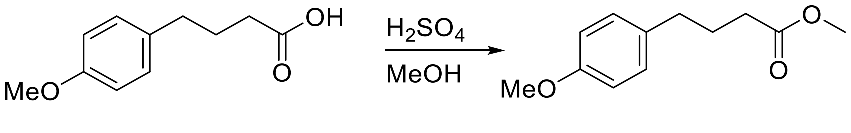

Synthesized according to a literature procedure.[S6] 4-(4-methoxyphenyl)butanoic acid (8.00 g; 41.2 mmol; 1.0 eq) was dissolved in MeOH (100 mL) and 10 drops of concentrated sulphuric acid were added. The solution was stirred for 16 h and concentrated under reduced pressure. Water (100 mL) was added and the emulsion was extracted with Et_2O (3x 100 mL), washed with brine (100 mL), dried over Na_2SO_4, filtered and concentrated. The product was obtained as a colorless oil (8.12 g; 39.0 mmol; 95%).

Spectral data were consistent with previously reported characterization of the product.[S6] ^{1}H NMR (300 MHz, $CDCl_3$) δ 7.14 – 7.05 (m, 2H), 6.87 – 6.78 (m, 2H), 3.79 (s, 3H), 3.66 (s, 3H), 2.59 (t, *J* = 7.6 Hz, 2H), 2.32 (t, *J* = 7.5 Hz, 2H), 2.00 – 1.87 (m, 2H). ^{13}C NMR (75 MHz, $CDCl_3$) δ 174.15, 158.03, 133.57, 129.51, 113.93, 55.39, 51.63, 34.34, 33.48, 26.86.

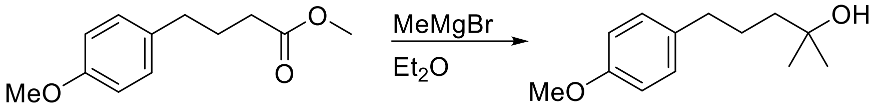

Synthesized according to a literature procedure.[S6] In an oven dried Schlenk under an argon atmosphere methyl 4-(4-methoxyphenyl)butanoate (8.07 g; 38.8 mmol; 1.0 eq) was dissolved in anhydrous Et_2O (200 mL) and cooled to 0 °C. A solution of 3.0 M MeMgBr (38.8 mL; 116 mmol; 3.0 eq) in Et_2O was added dropwise and the obtained white suspension was stirred for 16 h. The mixture was quenched with concentrated aqueous NH_4Cl (50 mL) solution and extracted with Et_2O (3x 100 mL), washed with brine (100 mL), dried over Na_2SO_4, filtered and concentrated. The product was obtained as a colorless oil (7.34 g; 35.2 mmol; 91%).

Spectral data were consistent with previously reported characterization of the product.[S6] ^{1}H NMR (300 MHz, $CDCl_3$) δ 7.15 – 7.06 (m, 2H), 6.87 – 6.78 (m, 2H), 3.79 (s, 3H), 2.57 (t, *J* = 7.5 Hz, 2H), 1.75 – 1.55 (m, 2H), 1.54 – 1.44 (m, 2H), 1.38 (d, *J* = 14.1 Hz, 1H), 1.20 (s, 6H). ^{13}C NMR (75 MHz, $CDCl_3$) δ 157.85, 134.67, 129.39, 113.86, 71.09, 55.39, 43.58, 35.54, 29.38, 26.63.

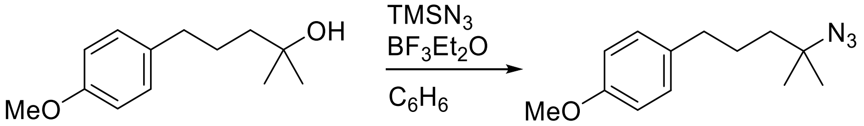

Synthesized according to a literature procedure.[S6] In an oven dried Schlenk under an argon atmosphere 5-(4-methoxyphenyl)-2-methylpentan-2-ol (6.88 g; 33.0 mmol; 1.0 eq) and $TMSN_3$ (5.3 mL; 40 mmol; 1.2 eq) was dissolved in anhydrous C_6H_6 (200 mL). BF_3Et_2O (4.9 mL; 40 mmol; 1.2 eq) was added dropwise and the solution was stirred at 60 °C for 40 h. The obtained mixture was quenched with water (100 mL), extracted with Et_2O (3x 100 mL), washed with brine (100 mL), dried over Na_2SO_4, filtered and concentrated. The crude product was purified by column chromatography over SiO_2 using hexane as eluent. The product was obtained as a colorless oil (0.93 g; 4.0 mmol; 12%).

Spectral data were consistent with previously reported characterization of the product.[S6] ^{1}H NMR (300 MHz, $CDCl_3$) δ 7.14 – 7.05 (m, 2H), 6.88 – 6.79 (m, 2H), 3.79 (s, 3H), 2.56 (t, *J* = 7.4 Hz, 2H), 1.72 – 1.57 (m, 2H), 1.56 – 1.46 (m, 2H), 1.24 (s, 6H). ^{13}C NMR (75 MHz, $CDCl_3$) δ 157.94, 134.26, 129.37, 113.92, 61.73, 55.40, 41.13, 35.25, 26.47, 26.13.

Substrate **5a**

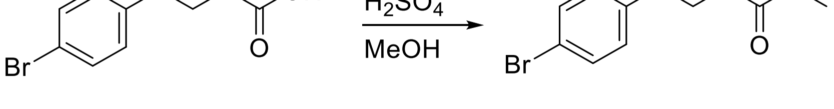

Synthesized according to a literature procedure.[S8] 4-(4-bromophenyl)butanoic acid (20.00 g; 82.3 mmol; 1.0 eq) was dissolved in MeOH (300 mL) and 10 drops of concentrated sulphuric acid were added. The solution was stirred for 16 h and concentrated under reduced pressure. Water (200 mL) was added and the emulsion was extracted with Et_2O (3x 200 mL), washed with brine (100 mL), dried over Na_2SO_4, filtered and concentrated. The product was obtained as a colorless oil (19.54 g; 76.0 mmol; 92%).

Spectral data were consistent with previously reported characterization of the product.[S8] ^{1}H NMR (300 MHz, $CDCl_3$) δ 7.44 – 7.35 (m, 2H), 7.09 – 7.00 (m, 2H), 3.66 (d, *J* = 1.2 Hz, 3H), 2.60 (t, *J* = 7.6 Hz, 2H), 2.31 (t, *J* = 7.4 Hz, 2H), 2.01 – 1.87 (m, 2H). ^{13}C NMR (75 MHz, CDCl3) δ 173.86, 140.43, 131.56, 130.36, 119.87, 51.68, 34.61, 33.33, 26.41.

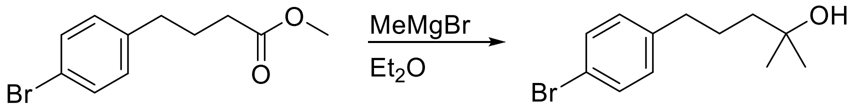

Synthesized according to a literature procedure.[S9] In an oven dried Schlenk under an argon atmosphere methyl 4-(4-bromophenyl)butanoate (19.54 g; 72.06 mmol; 1.0 eq) was dissolved in anhydrous Et_2O (400 mL) and cooled to 0 °C. A solution of 3.0 M MeMgBr (72.1 mL; 216 mmol; 3.0 eq) in Et_2O was added dropwise and the obtained white suspension was stirred for 16 h. The mixture was quenched with concentrated aqueous NH_4Cl (100 mL) solution and extracted with Et_2O (3x 200 mL), washed with brine (100 mL), dried over Na_2SO_4, filtered and concentrated. The product was obtained as a colorless oil (16.77 g; 61.8 mmol; 86%).

Spectral data were consistent with previously reported characterization of the product.[S9] ^{1}H NMR (300 MHz, $CDCl_3$) δ 7.44 – 7.35 (m, 2H), 7.11 – 7.01 (m, 2H), 2.58 (td, *J* = 7.6, 2.0 Hz, 2H), 1.76 – 1.59 (m, 2H), 1.54 – 1.43 (m, 2H), 1.28 (d, *J* = 3.2 Hz, 1H), 1.20 (d, *J* = 2.0 Hz, 6H). ^{13}C NMR (75 MHz, $CDCl_3$) δ 141.48, 131.47, 130.31, 119.58, 70.99, 43.40, 35.82, 29.43, 26.17.

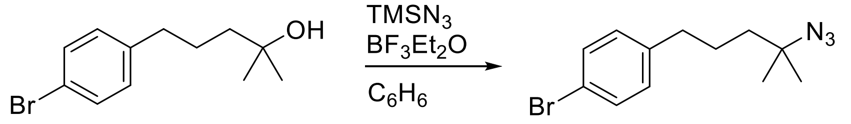

Synthesized according to a literature procedure.[S10] In an oven dried Schlenk under an argon atmosphere 5-(4-bromophenyl)-2-methylpentan-2-ol (4.27 g; 16.6 mmol; 1.0 eq) and $TMSN_3$ (2.6 mL; 20 mmol; 1.2 eq) was dissolved in anhydrous C_6H_6 (200 mL). BF_3Et_2O (2.5 mL; 20 mmol; 1.2 eq) was added dropwise and the solution was stirred for 16 h. The obtained mixture was quenched with water (100 mL), extracted with Et_2O (3x 100 mL), washed with brine (100 mL), dried over Na_2SO_4, filtered and concentrated. The crude product was purified by column chromatography over SiO_2 using hexane as eluent. The product was obtained as a colorless oil (2.55 g; 9.0 mmol; 54%).

Spectral data were consistent with previously reported characterization of the product.[S10] ^{1}H NMR (300 MHz, $CDCl_3$) δ 7.44 – 7.35 (m, 2H), 7.11 – 7.01 (m, 2H), 2.57 (t, *J* = 7.5 Hz, 2H), 1.74

– 1.59 (m, 2H), 1.53 – 1.45 (m, 2H), 1.24 (s, 6H). ^{13}C NMR (75 MHz, $CDCl_3$) δ 141.08, 131.56, 130.27, 119.74, 61.60, 41.05, 35.52, 26.13, 26.02.

Substrate **6a**

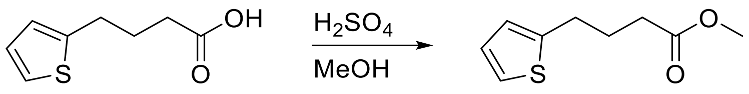

Synthesized according to a literature procedure.[S11] 4-(thiophen-2-yl)butanoic acid (8.05 g; 47.3 mmol; 1.0 eq) was dissolved in MeOH (100 mL) and 10 drops of concentrated sulphuric acid were added. The solution was stirred for 16 h and concentrated under reduced pressure. Water (100 mL) was added and the emulsion was extracted with Et_2O (3x 100 mL), washed with brine (100 mL), dried over Na_2SO_4, filtered and concentrated. The product was obtained as a brown oil (8.00 g; 43.4 mmol; 92%).

Spectral data were consistent with previously reported characterization of the product.[S11] ^{1}H NMR (300 MHz, $CDCl_3$) δ 7.12 (dd, *J* = 5.1, 1.2 Hz, 1H), 6.92 (dd, *J* = 5.1, 3.4 Hz, 1H), 6.80 (dq, *J* = 3.3, 1.0 Hz, 1H), 3.68 (s, 3H), 2.97 – 2.82 (m, 2H), 2.38 (t, *J* = 7.4 Hz, 2H), 2.01 (p, *J* = 7.5 Hz, 2H).

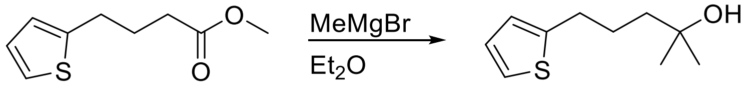

Synthesized according to a literature procedure.[S11] In an oven dried Schlenk under an argon atmosphere methyl 4-(thiophen-2-yl)butanoate (8.00 g; 43.4 mmol; 1.0 eq) was dissolved in anhydrous Et_2O (200 mL) and cooled to 0 °C. A solution of 3.0 M MeMgBr (43.4 mL; 130 mmol; 3.0 eq) in Et_2O was added dropwise and the obtained white suspension was stirred for 16 h. The mixture was quenched with concentrated aqueous NH_4Cl (50 mL) solution and extracted with Et_2O (3x 100 mL), washed with brine (100 mL), dried over Na_2SO_4, filtered and concentrated. The product was obtained as a brown oil (6.78 g; 36.8 mmol; 85%).

Spectral data were consistent with previously reported characterization of the product.[S11] ^{1}H NMR (300 MHz, $CDCl_3$) δ 7.11 (dd, *J* = 5.1, 1.2 Hz, 1H), 6.92 (dd, *J* = 5.1, 3.4 Hz, 1H), 6.79 (dq, *J* = 3.3, 1.0 Hz, 1H), 2.85 (td, *J* = 7.5, 1.0 Hz, 2H), 1.85 – 1.66 (m, 2H), 1.60 – 1.48 (m, 2H), 1.35 – 1.28 (m, 1H), 1.22 (s, 6H). ^{13}C NMR (75 MHz, $CDCl_3$) δ 145.47, 126.83, 124.25, 123.04, 71.01, 43.36, 30.43, 29.41, 26.78.

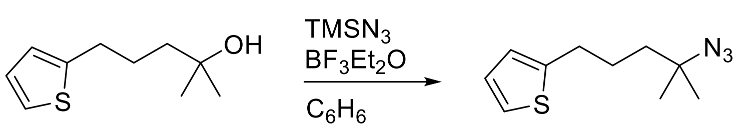

Synthesized according to a literature procedure.[S11] In an oven dried Schlenk under an argon atmosphere 2-methyl-5-(thiophen-2-yl)pentan-2-ol (6.67 g; 36.2 mmol; 1.0 eq) and $TMSN_3$ (5.8 mL; 43 mmol; 1.2 eq) was dissolved in anhydrous C_6H_6 (200 mL). BF_3Et_2O (5.4 mL; 43 mmol; 1.2 eq) was added dropwise and the solution was stirred for 16 h. The obtained mixture

was quenched with water (100 mL), extracted with Et_2O (3x 100 mL), washed with brine (100 mL), dried over Na_2SO_4, filtered and concentrated. The crude product was purified by column chromatography over SiO_2 using hexane as eluent. The product was obtained as a colorless oil (1.40 g; 6.7 mmol; 18%).

Spectral data were consistent with previously reported characterization of the product.[S11] ^{1}H NMR (300 MHz, $CDCl_3$) δ 7.11 (dd, *J* = 5.1, 1.2 Hz, 1H), 6.92 (dd, *J* = 5.1, 3.4 Hz, 1H), 6.79 (dq, *J* = 3.3, 1.0 Hz, 1H), 2.85 (td, *J* = 7.5, 1.0 Hz, 2H), 1.85 – 1.66 (m, 2H), 1.60 – 1.48 (m, 2H), 1.35 – 1.28 (m, 1H), 1.22 (s, 6H). ^{13}C NMR (75 MHz, $CDCl_3$) δ 145.47, 126.83, 124.25, 123.04, 71.01, 43.36, 30.43, 29.41, 26.78.

Substrate **7a**

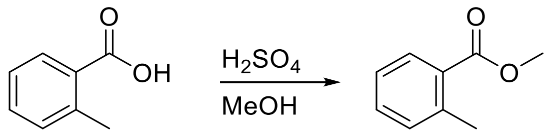

Synthesized according to a literature procedure.[S10] 2-methylbenzoic acid (8.00 g; 58.8 mmol; 1.0 eq) was dissolved in MeOH (100 mL) and 10 drops of concentrated sulphuric acid were added. The solution was stirred for 72 h at 60 °C and concentrated under reduced pressure. Water (100 mL) was added and the emulsion was extracted with Et_2O (3x 100 mL), washed with brine (100 mL), dried over Na_2SO_4, filtered and concentrated. The product was obtained as a colorless oil (8.14 g; 54.2 mmol; 92%).

Spectral data were consistent with previously reported characterization of the product.[S10] ^{1}H NMR (300 MHz, $CDCl_3$) δ 7.83 (dd, *J* = 8.1, 1.5 Hz, 1H), 7.32 (td, *J* = 7.5, 1.5 Hz, 1H), 7.23 – 7.10 (m, 2H), 3.81 (s, 3H), 2.53 (s, 3H). ^{13}C NMR (75 MHz, $CDCl_3$) δ 168.21, 140.30, 132.08, 131.80, 130.68, 129.69, 125.81, 51.92, 21.84.

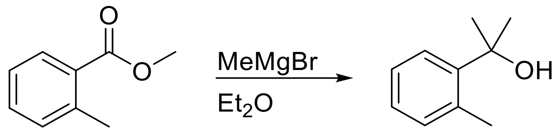

Synthesized according to a literature procedure.[S10] In an oven dried Schlenk under an argon atmosphere methyl 2-methylbenzoate (8.14 g; 54.2 mmol; 1.0 eq) was dissolved in anhydrous Et_2O (200 mL) and cooled to 0 °C. A solution of 3.0 M MeMgBr (54.2 mL; 163 mmol; 3.0 eq) in Et_2O was added dropwise and the obtained white suspension was stirred for 16 h. The mixture was quenched with concentrated aqueous NH_4Cl (50 mL) solution and extracted with Et_2O (3x 100 mL), washed with brine (100 mL), dried over Na_2SO_4, filtered and concentrated. The product was obtained as a colorless oil (7.28 g; 48.5 mmol; 89%).

Spectral data were consistent with previously reported characterization of the product.[S10] ^{1}H NMR (300 MHz, $CDCl_3$) δ 7.51 – 7.40 (m, 1H), 7.20 – 7.13 (m, 3H), 2.61 (s, 3H), 1.67 (s, 6H). ^{13}C NMR (75 MHz, $CDCl_3$) δ 145.85, 136.03, 132.78, 127.15, 125.76, 125.34, 73.79, 30.95, 22.33.

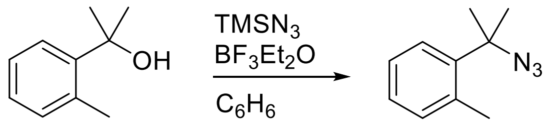

Synthesized according to a literature procedure.[S10] In an oven dried Schlenk under an argon atmosphere 2-(o-tolyl)propan-2-ol (7.14 g; 47.5 mmol; 1.0 eq) and $TMSN_3$ (7.6 mL; 57 mmol; 1.2 eq) was dissolved in anhydrous C_6H_6 (200 mL). BF_3Et_2O (7.0 mL; 57 mmol; 1.2 eq) was added dropwise and the solution was stirred for 16 h. The obtained mixture was quenched with water (100 mL), extracted with Et_2O (3x 100 mL), washed with brine (100 mL), dried over Na_2SO_4, filtered and concentrated. The crude product was purified by column chromatography over SiO_2 using hexane as eluent. The product was obtained as a colorless oil (3.60 g; 20.5 mmol; 43%).

Spectral data were consistent with previously reported characterization of the product.[S10] ^1H NMR (300 MHz, $CDCl_3$) δ 7.33 – 7.21 (m, 1H), 7.17 – 7.02 (m, 3H), 2.52 (s, 3H), 1.61 (s, 6H). ^{13}C NMR (75 MHz, $CDCl_3$) δ 141.22, 136.58, 133.01, 127.88, 126.04, 125.91, 64.57, 27.91, 21.68.

Substrate **8a**

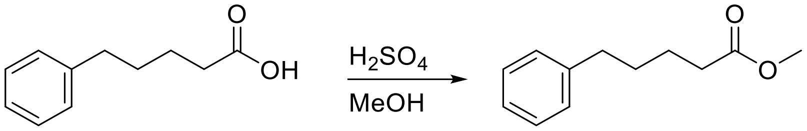

Synthesized according to a literature procedure.[S12] 5-phenylpentanoic acid (8.00 g; 44.9 mmol; 1.0 eq) was dissolved in MeOH (100 mL) and 10 drops of concentrated sulphuric acid were added. The solution was stirred for 16 h and concentrated under reduced pressure. Water (100 mL) was added and the emulsion was extracted with Et_2O (3x 100 mL), washed with brine (100 mL), dried over Na_2SO_4, filtered and concentrated. The product was obtained as a colorless oil (7.92 g; 41.2 mmol; 92%).

Spectral data were consistent with previously reported characterization of the product.[S12] ^1H NMR (300 MHz, $CDCl_3$) δ 7.25 – 7.16 (m, 2H), 7.15 – 7.06 (m, 3H), 3.59 (s, 3H), 2.62 – 2.49 (m, 2H), 2.34 – 2.20 (m, 2H), 1.69 – 1.49 (m, 4H). ^{13}C NMR (75 MHz, $CDCl_3$) δ 174.22, 142.26, 128.51, 128.45, 125.91, 51.62, 35.70, 34.08, 31.03, 24.72.

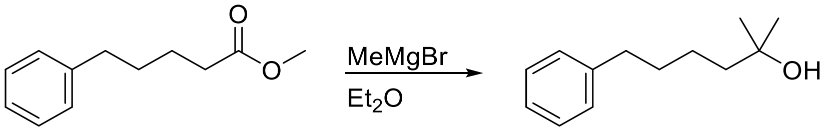

Synthesized according to a literature procedure.[S13] In an oven dried Schlenk under an argon atmosphere methyl 5-phenylpentanoate (7.92 g; 41.2 mmol; 1.0 eq) was dissolved in anhydrous Et_2O (200 mL) and cooled to 0 °C. A solution of 3.0 M MeMgBr (41.2 mL; 124 mmol; 3.0 eq) in Et_2O was added dropwise and the obtained white suspension was stirred for 16 h. The mixture was quenched with concentrated aqueous NH_4Cl (50 mL) solution and extracted with Et_2O (3x 100 mL), washed with brine (100 mL), dried over Na_2SO_4, filtered and concentrated. The product was obtained as a colorless oil (7.28 g; 37.9 mmol; 92%).

Spectral data were consistent with previously reported characterization of the product.[S13] ^{1}H NMR (300 MHz, $CDCl_3$) δ 7.28 – 7.16 (m, 2H), 7.16 – 6.99 (m, 3H), 2.56 (t, *J* = 8.1 Hz, 2H), 1.67 – 1.49 (m, 2H), 1.49 – 1.39 (m, 2H), 1.39 – 1.26 (m, 2H), 1.21 (s, 1H), 1.13 (s, 6H). ^{13}C NMR (75 MHz, $CDCl_3$) δ 142.77, 128.52, 128.41, 125.79, 71.14, 43.90, 36.09, 32.18, 29.39, 24.19.

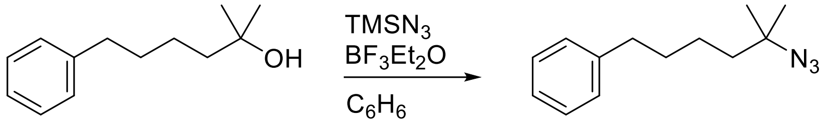

Synthesized according to a literature procedure.[S14] In an oven dried Schlenk under an argon atmosphere 2-methyl-6-phenylhexan-2-ol (7.23 g; 37.6 mmol; 1.0 eq) and $TMSN_3$ (6.0 mL; 45 mmol; 1.2 eq) was dissolved in anhydrous C_6H_6 (200 mL). BF_3Et_2O (5.6 mL; 45 mmol; 1.2 eq) was added dropwise and the solution was stirred for 16 h. The obtained mixture was quenched with water (100 mL), extracted with Et_2O (3x 100 mL), washed with brine (100 mL), dried over Na_2SO_4, filtered and concentrated. The crude product was purified by column chromatography over SiO_2 using hexane as eluent. The product was obtained as a colorless oil (3.69 g; 17.0 mmol; 45%).

Spectral data were consistent with previously reported characterization of the product.[S14] ^{1}H NMR (300 MHz, $CDCl_3$) δ 7.26 – 7.15 (m, 2H), 7.15 – 7.05 (m, 3H), 2.55 (t, 2H), 1.62 – 1.49 (m, 2H), 1.49 – 1.40 (m, 2H), 1.40 – 1.26 (m, 2H), 1.17 (s, 6H). ^{13}C NMR (75 MHz, $CDCl_3$) δ 142.57, 128.50, 128.44, 125.85, 61.79, 41.44, 35.99, 31.89, 26.13, 24.10.

Substrate **9a**

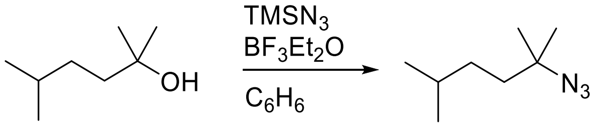

Synthesized according to a literature procedure.[S5] In an oven dried Schlenk under an argon atmosphere 2,5-dimethylhexan-2-ol (5.25 g; 40.3 mmol; 1.0 eq) and $TMSN_3$ (6.4 mL; 48 mmol; 1.2 eq) was dissolved in anhydrous C_6H_6 (200 mL). BF_3Et_2O (6.0 mL; 48 mmol; 1.2 eq) was added dropwise and the solution was stirred for 16 h. The obtained mixture was quenched with water (100 mL), extracted with Et_2O (3x 100 mL), washed with brine (100 mL), dried over Na_2SO_4, filtered and concentrated. The crude product was purified by column chromatography over SiO_2 using hexane as eluent. The product was obtained as a colorless oil (3.14 g; 20.2 mmol; 50%).

Spectral data were consistent with previously reported characterization of the product.[S5] ^{1}H NMR (300 MHz, $CDCl_3$) δ 1.58 – 1.42 (m, 3H), 1.30 – 1.16 (m, 8H), 0.90 (d, *J* = 6.6 Hz, 6H). ^{13}C NMR (75 MHz, $CDCl_3$) δ 61.88, 39.37, 33.34, 28.49, 26.14, 22.72.

Substrate **10a**

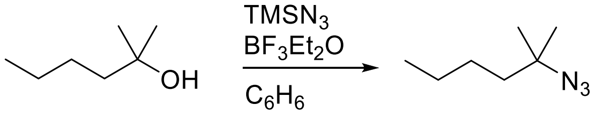

Synthesized according to a literature procedure.[S5] In an oven dried Schlenk under an argon atmosphere 2-methylhexan-2-ol (5.23 g; 45.0 mmol; 1.0 eq) and $TMSN_3$ (7.2 mL; 54 mmol; 1.2 eq) was dissolved in anhydrous C_6H_6 (200 mL). BF_3Et_2O (6.7 mL; 54 mmol; 1.2 eq) was added dropwise and the solution was stirred for 16 h. The obtained mixture was quenched with water (100 mL), extracted with Et_2O (3x 100 mL), washed with brine (100 mL), dried over Na_2SO_4, filtered and concentrated. The crude product was purified by column chromatography over SiO_2 using hexane as eluent. The product was obtained as a colorless oil (3.69 g; 26.1 mmol; 58%).

Spectral data were consistent with previously reported characterization of the product.[S5] ^{1}H NMR (300 MHz, $CDCl_3$) δ 1.54 – 1.42 (m, 2H), 1.42 – 1.27 (m, 4H), 1.25 (s, 6H), 0.99 – 0.84 (m, 3H). ^{13}C NMR (75 MHz, $CDCl_3$) δ 61.83, 41.32, 26.57, 26.13, 23.15, 14.16.

<u>Substrate **11a**</u>

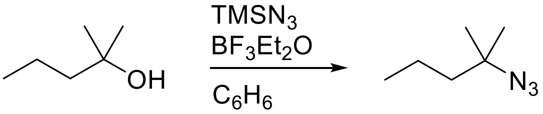

Synthesized according to a literature procedure.[S5] In an oven dried Schlenk under an argon atmosphere 2-methylpentan-2-ol (5.25 g; 51.4 mmol; 1.0 eq) and $TMSN_3$ (8.2 mL; 62 mmol; 1.2 eq) was dissolved in anhydrous C_6H_6 (200 mL). BF_3Et_2O (7.6 mL; 62 mmol; 1.2 eq) was added dropwise and the solution was stirred for 16 h. The obtained mixture was quenched with water (100 mL), extracted with Et_2O (3x 100 mL), washed with brine (100 mL), dried over Na_2SO_4, filtered and concentrated. The crude product was purified by column chromatography over SiO_2 using hexane as eluent. The product was obtained as a colorless oil (4.99 g; 51.4 mmol; 76%).

Spectral data were consistent with previously reported characterization of the product.[S5] ^{1}H NMR (300 MHz, $CDCl_3$) δ 1.53 – 1.30 (m, 4H), 1.25 (s, 6H), 0.93 (t, J = 7.4 Hz, 3H). ^{13}C NMR (75 MHz, $CDCl_3$) δ 61.83, 43.90, 26.13, 17.70, 14.52.

<u>Substrate **12a**</u>

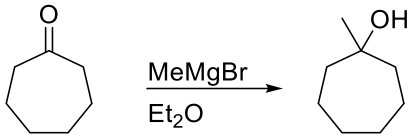

Synthesized according to a literature procedure.[S15] In an oven dried Schlenk under an argon atmosphere cycloheptanone (5.15 g; 45.9 mmol; 1.0 eq) was dissolved in anhydrous Et_2O (200 mL) and cooled to 0 °C. A solution of 3.0 M MeMgBr (30.6 mL; 91.8 mmol; 2.0 eq) in Et_2O was added dropwise and the obtained white suspension was stirred for 16 h. The mixture was quenched with concentrated aqueous NH_4Cl (50 mL) solution and extracted with Et_2O (3x 100 mL), washed with brine (100 mL), dried over Na_2SO_4, filtered and concentrated. The product was obtained as a colorless oil (4.28 g; 33.4 mmol; 73%).

Spectral data were consistent with previously reported characterization of the product.[S15] ^1H NMR (300 MHz, $CDCl_3$) δ 1.76 – 1.45 (m, 12H), 1.44 – 1.29 (m, 3H), 1.22 (s, 3H). ^{13}C NMR (75 MHz, $CDCl_3$) δ 74.10, 43.17, 31.29, 29.85, 22.76.

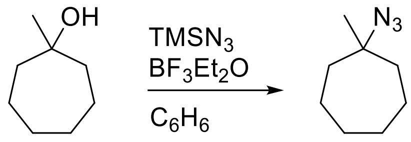

Synthesized according to a literature procedure.[S15] In an oven dried Schlenk under an argon atmosphere 1-methylcycloheptan-1-ol (4.13 g; 32.2 mmol; 1.0 eq) and $TMSN_3$ (5.1 mL; 39 mmol; 1.2 eq) was dissolved in anhydrous C_6H_6 (200 mL). BF_3Et_2O (4.8 mL; 39 mmol; 1.2 eq) was added dropwise and the solution was stirred for 16 h. The obtained mixture was quenched with water (100 mL), extracted with Et_2O (3x 100 mL), washed with brine (100 mL), dried over Na_2SO_4, filtered and concentrated. The crude product was purified by column chromatography over SiO_2 using hexane as eluent. The product was obtained as a colorless oil (2.12 g; 13.8 mmol; 43%).

Spectral data were consistent with previously reported characterization of the product.[S15] ^1H NMR (300 MHz, $CDCl_3$) δ 1.86 – 1.33 (m, 12H), 1.29 (s, 3H). ^{13}C NMR (75 MHz, $CDCl_3$) δ 65.36, 40.24, 29.44, 27.59, 22.68.

<u>Substrate **13a**</u>

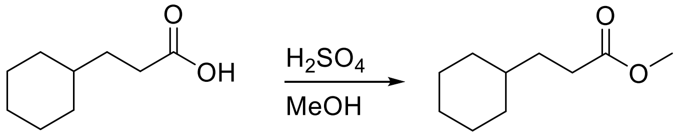

Synthesized according to a literature procedure.[S16] 3-cyclohexylpropanoic acid (8.06 g; 51.6 mmol; 1.0 eq) was dissolved in MeOH (100 mL) and 10 drops of concentrated sulphuric acid were added. The solution was stirred for 72 h at 60 °C and concentrated under reduced pressure. Water (100 mL) was added and the emulsion was extracted with Et_2O (3x 100 mL), washed with brine (100 mL), dried over Na_2SO_4, filtered and concentrated. The product was obtained as a colorless oil (8.22 g; 48.3 mmol; 94%).

Spectral data were consistent with previously reported characterization of the product.[S16] ^1H NMR (300 MHz, $CDCl_3$) δ 3.60 (s, 3H), 2.25 (t, J = 7.7 Hz, 2H), 1.73 – 1.51 (m, 5H), 1.46 (q, J = 7.2 Hz, 2H), 1.27 – 0.97 (m, 4H), 0.92 – 0.74 (m, 2H). ^{13}C NMR (75 MHz, $CDCl_3$) δ 174.52, 51.39, 37.24, 32.98, 32.36, 31.65, 26.55, 26.23.

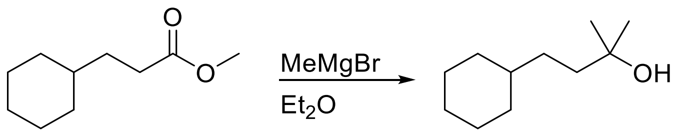

Synthesized according to a literature procedure.[S17] In an oven dried Schlenk under an argon atmosphere methyl 3-cyclohexylpropanoate (8.08 g; 47.5 mmol; 1.0 eq) was dissolved in anhydrous Et_2O (200 mL) and cooled to 0 °C. A solution of 3.0 M MeMgBr (47.5 mL; 122 mmol;

3.0 eq) in Et_2O was added dropwise and the obtained white suspension was stirred for 16 h. The mixture was quenched with concentrated aqueous NH_4Cl (50 mL) solution and extracted with Et_2O (3x 100 mL), washed with brine (100 mL), dried over Na_2SO_4, filtered and concentrated. The product was obtained as a colorless oil (5.67 g; 33.3 mmol; 70%).

Spectral data were consistent with previously reported characterization of the product.[S17] ^1H NMR (300 MHz, $CDCl_3$) δ 1.77 – 1.53 (m, 7H), 1.51 – 1.36 (m, 2H), 1.34 – 1.00 (m, 13H), 0.94 – 0.75 (m, 2H). ^{13}C NMR (75 MHz, $CDCl_3$) δ 71.08, 41.30, 38.27, 33.51, 31.99, 29.20, 26.76, 26.46.

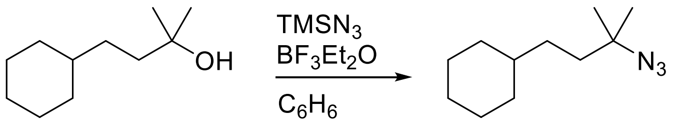

Synthesized according to a literature procedure.[S10] In an oven dried Schlenk under an argon atmosphere 4-cyclohexyl-2-methylbutan-2-ol (5.67 g; 33.3 mmol; 1.0 eq) and $TMSN_3$ (5.3 mL; 40 mmol; 1.2 eq) was dissolved in anhydrous C_6H_6 (200 mL). BF_3Et_2O (4.9 mL; 40 mmol; 1.2 eq) was added dropwise and the solution was stirred for 16 h. The obtained mixture was quenched with water (100 mL), extracted with Et_2O (3x 100 mL), washed with brine (100 mL), dried over Na_2SO_4, filtered and concentrated. The crude product was purified by column chromatography over SiO_2 using hexane as eluent. The product was obtained as a colorless oil (2.27 g; 11.6 mmol; 35%).

Spectral data were consistent with previously reported characterization of the product.[S10] ^1H NMR (300 MHz, $CDCl_3$) δ 1.79 – 1.57 (m, 5H), 1.54 – 1.40 (m, 2H), 1.36 – 1.01 (m, 12H), 0.98 – 0.79 (m, 2H). ^{13}C NMR (75 MHz, $CDCl_3$) δ 61.92, 38.89, 38.15, 33.51, 31.90, 26.78, 26.49, 26.12.

Catalysis

General procedure

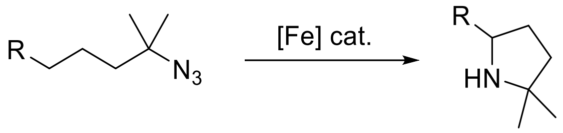

Inside an argon filled glovebox, the iron catalyst was weighed into a J Young NMR tube. A stock solution of internal standard was made by dissolving 1,3,5-trimethoxybenzene (45.5 mg; 0.0271 mmol) in 1 mL of deuterated solvent. The corresponding azide (25 mmol) was weighed into a vial, to which internal standard stock solution (0.1 mL) and deuterated solvent (0.4 mL) were added. The contents of the vial were transferred into a J Young NMR tube containing the iron catalyst. The NMR tube was taken outside the glovebox and heated in an oil bath (Figure S1). For analysis, the reaction was exposed to air, by transferring the contents of the NMR tube in a vial and adding pentane (5 mL). This mixture was left for 16 hours after which small amounts of precipitate formed. Part of the solution was decanted and concentrated to dryness to determine the yields by ^{1}H NMR spectroscopy.

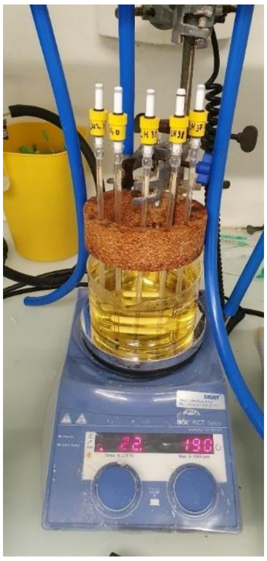

Figure S1: Typical setup for running catalytic experiments.

Catalysis with low catalyst loadings

Due to practical reason, it was impossible to weigh the catalyst directly in the NMR tube for 0.5 and 0.1% mol% catalyst loadings. For these experiments, a known amount of FeI_2 was mixed in 1.00 g of 1,3,5-trimethoxybenzene and ground with a pestle and mortal until a homogeneous powder was obtained. This stock ''solid'' was used to weigh out small amounts of FeI_2 in the NMR tube for catalysis.

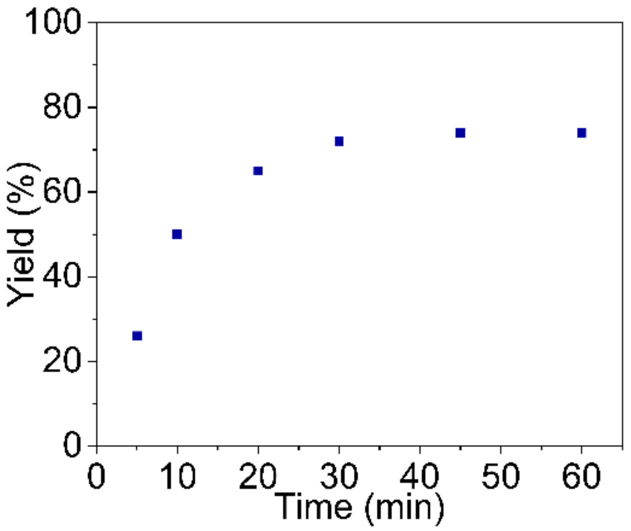

Figure S2. Evolution of yield over time of the cyclic amine **1b** using 5 mol% FeI_2 in toluene-d$_8$ at 120 °C.

Yields are most likely underestimated as some of the cyclic amine product binds to the iron complex, making it undetectable in ^{1}H NMR spectroscopy due to its paramagnetic nature. This caused the highest measured TOF of 60 h^{-1} to be underestimated as well.

Solvent scope

Since FeI_2 dissolves only poorly in toluene,[S18] more polar solvents were tested for this transformation. However, either using DMF-d$_7$ or DMSO-d$_6$ at 120 °C fully inhibited C–H amination (

Table S1, entries 1-2), presumably because of the coordinating ability of these solvents, which prevents any substrate binding. On the other hand, using THF-d$_8$ at 100 °C resulted in a modest 10% yield after 30 min, despite its coordinating ability (entry 3). In comparison, using toluene-d$_8$ at 100 °C gave 40% yield after 30 min (entry 4), and 72% at 120 °C (entry 5). A similar drop in activity in THF-d$_8$ was observed in previous work using $Fe(HMDS)_2$. These data therefore indicate a strong preference for non-coordinating solvents, when performing C–H amination with FeI_2 or other iron catalysts that lack a sophisticated ligand stabilization.

Table S1: Use of different solvents for the FeI_2 catalysed intramolecular C–H amination.

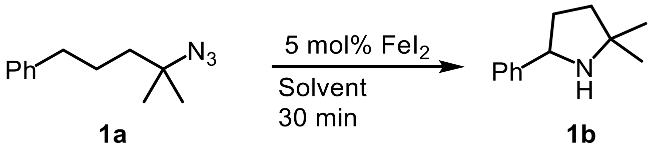

Entry[a]	Solvent	Temperature (°C)	Yield (%)[b]
1	DMF-d$_7$	120	0
2	DMSO-d$_6$	120	0
3	THF-d$_8$	100	10
4	Toluene-d$_8$	100	40

5	Toluene-d_8	120	72

[a] Catalysis was performed on a 0.25 mmol scale in J Young NMR tubes; see SI for exact experimental details. [b] Yields and conversions were determined by ^{1}H NMR spectroscopy using 1,3,5-trimethoxybenzene as internal standard.

Effect of additives

Introduction of potential ligands as additives to FeI_2 was investigated. Addition of 1 equiv PPh_3, under otherwise identical conditions using the model substrate, resulted in a 74% yield of cyclic amine **1b**, identical to runs in the absence of additive (Table S2, entries 1-2). Similarly, pyridine exerted no significant influence on the catalytic activity (entry 3). Contrastingly, multidentate ligands such as 2,2'-bipyridine or 2,2';6',2"-terpyridine fully inhibited catalytic activity (entries 4-5). This catalyst poisoning further suggests a homogeneous mode of operation of this catalyst. Performing the catalytic runs with substrate **9a**, which only gave trace amounts of **9b** in absence of additives, did not show any product formation using PPh_3 or pyridine as additive. This shows that addition of simple ligands does not suppress the tentative product inhibition.

Table S2: Use of different additives for the FeI_2 catalysed intramolecular C–H amination.

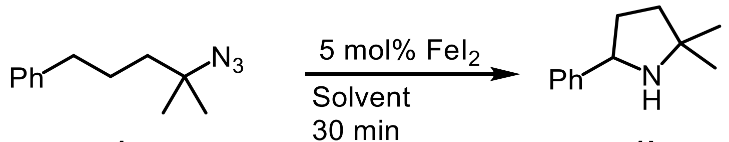

Entry[a]	Additive (5 mol%)	Yield (%)[b]
1	None	72
2	PPh_3	74
3	Pyridine	72
4	2,2'-Bipyridine	0
5	2,2';6',2"-Terpyridine	0

[a] Catalysis was performed on a 0.25 mmol scale in J Young NMR tubes; see SI for exact experimental details. [b] Yields and conversions were determined by ^{1}H NMR spectroscopy using 1,3,5-trimethoxybenzene as internal standard.

Radical trapping

A standard catalytic run was performed using 5 mol% of FeI_2 catalyst. After 2 min at 120 °C 50 mol% of (2,2,6,6-Tetramethylpiperidin-1-yl)oxyl (TEMPO) was added. The reaction was heated for another 28 min and quenched afterwards. This resulted in a conversion of 14%, significantly lower than without addition of TEMPO (Figure S2). This indicates a radical type mechanism, as TEMPO inhibits further conversion upon its addition.

Characterization of C–H aminated products

All products were characterized as crude mixtures after catalysis was completed, unless stated otherwise.

Product **1b**

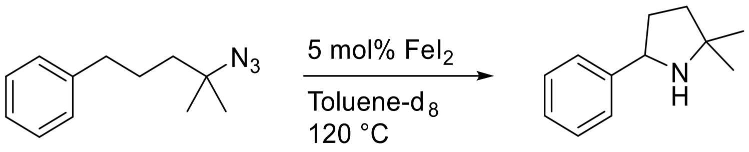

Spectral data were consistent with previously reported characterization of the product.[S14] ^{1}H NMR (300 MHz, $CDCl_3$) δ 7.36 – 7.06 (m, 5H), 4.25 (t, *J* = 7.6 Hz, 1H), 2.32 – 2.03 (m, 1H), 1.84 – 1.54 (m, 4H), 1.21 (s, 3H), 1.18 (s, 3H).

Product **3b**

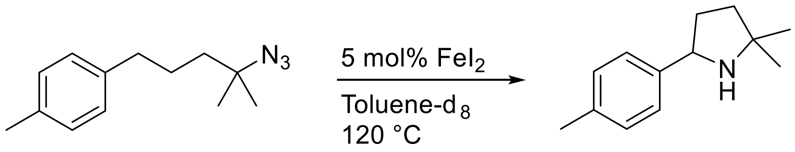

Spectral data were consistent with previously reported characterization of the product.[S14] ^{1}H NMR (300 MHz, $CDCl_3$) δ 7.26 – 7.14 (m, 2H), 7.09 – 6.95 (m, 2H), 4.21 (t, *J* = 7.6 Hz, 1H), 2.25 (s, 3H), 2.22 – 2.07 (m, 1H), 1.80 – 1.54 (m, 4H), 1.21 (s, 3H), 1.17 (s, 3H).

Product **4b**

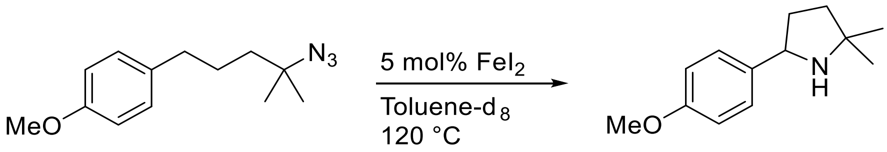

Spectral data were consistent with previously reported characterization of the product.[S14] ^{1}H NMR (300 MHz, $CDCl_3$) δ 7.31 – 7.17 (m, 2H), 6.81 – 6.72 (m, 2H), 4.19 (t, *J* = 7.5 Hz, 1H), 3.71 (s, 3H), 2.21 – 2.05 (m, 1H), 1.82 – 1.50 (m, 4H), 1.20 (s, 4H), 1.17 (s, 4H).

Product **5b**

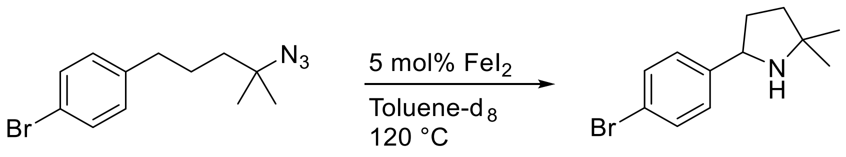

Spectral data were consistent with previously reported characterization of the product.[S14] ^{1}H NMR (300 MHz, $CDCl_3$) δ 7.38 – 7.27 (m, 2H), 7.23 – 7.13 (m, 2H), 4.21 (t, *J* = 7.3 Hz, 1H), 2.24 – 2.06 (m, 1H), 1.74 – 1.56 (m, 4H), 1.19 (s, 3H), 1.17 (s, 3H).

Product **6b**

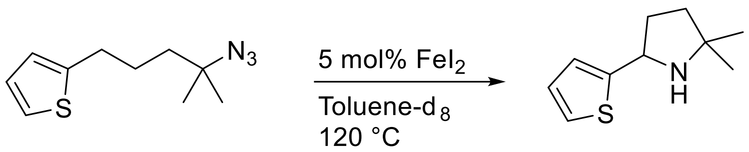

Spectral data were consistent with previously reported characterization of the product.[S14] ^{1}H NMR (300 MHz, $CDCl_3$) δ 7.11 – 7.03 (m, 1H), 6.90 – 6.78 (m, 2H), 4.50 (t, *J* = 7.5 Hz, 1H), 2.31 – 2.14 (m, 1H), 1.99 – 1.76 (m, 2H), 1.76 – 1.51 (m, 2H), 1.20 (s, 3H), 1.16 (s, 3H).

Product **8b**

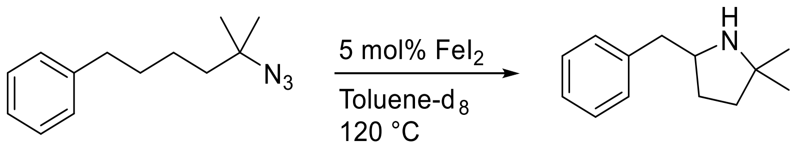

Due to low yields and overlapping signals with starting material and side products, not all signals of **8b** could be labelled. However, the formation of **8b** was confirmed by characteristic signals consistent with previously reported characterization of the product.[S14]

Product **12b**

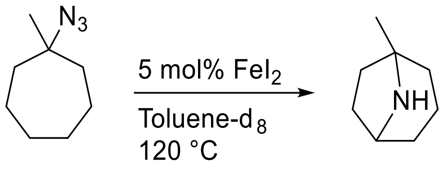

Due to low yields and overlapping signals with starting material and side products, not all signals of **12b** could be labelled. However, the formation of **12b** was confirmed by characteristic signals consistent with previously reported characterization of the product.[S14]

Product **13b**

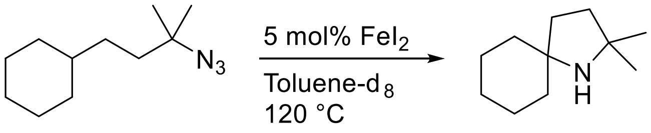

Due to low yields and overlapping signals with starting material and side products, not all signals of **13b** could be labelled. However, the formation of **13b** was confirmed by characteristic signals consistent with previously reported characterization of the product.[S14]

NMR spectra

All NMR spectra of the syntheses of substrates **1a**-**13a** have been reported previously,[S14] and included here for the sake of convenience and completion (Figure S40-S98).

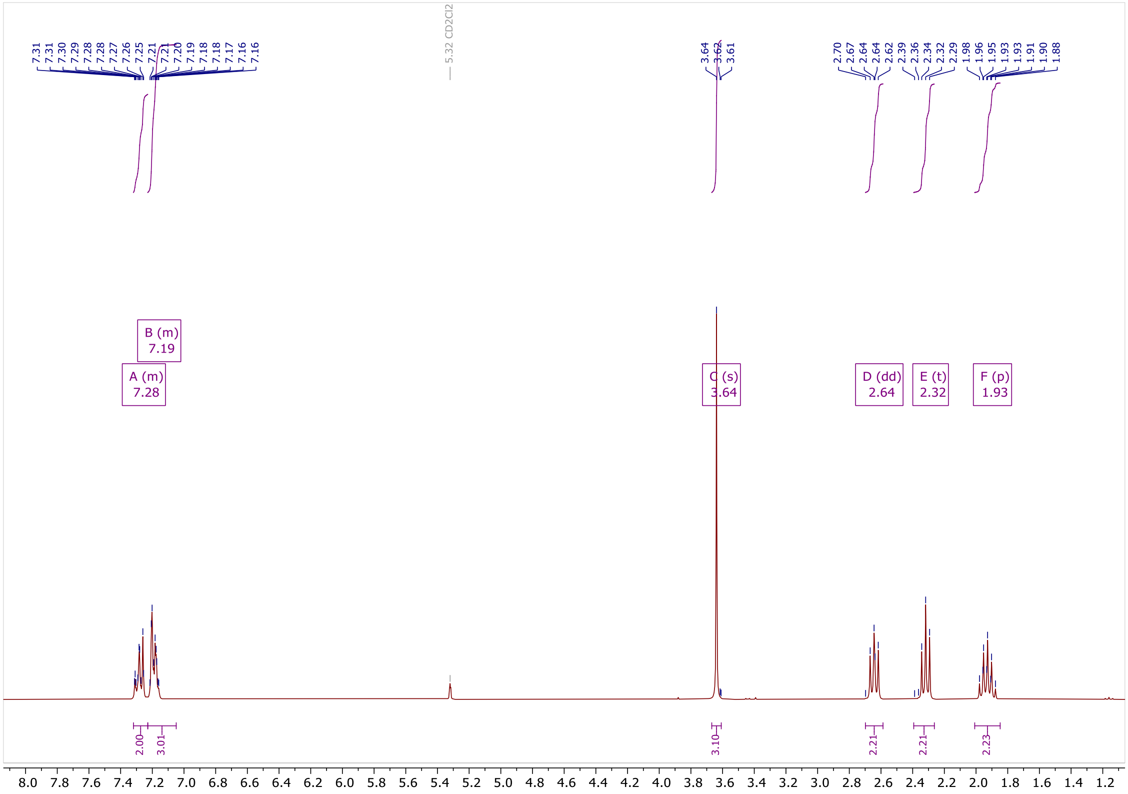

Figure S3: ^{1}H NMR spectrum of methyl 4-phenylbutanoate in CD_2Cl_2.

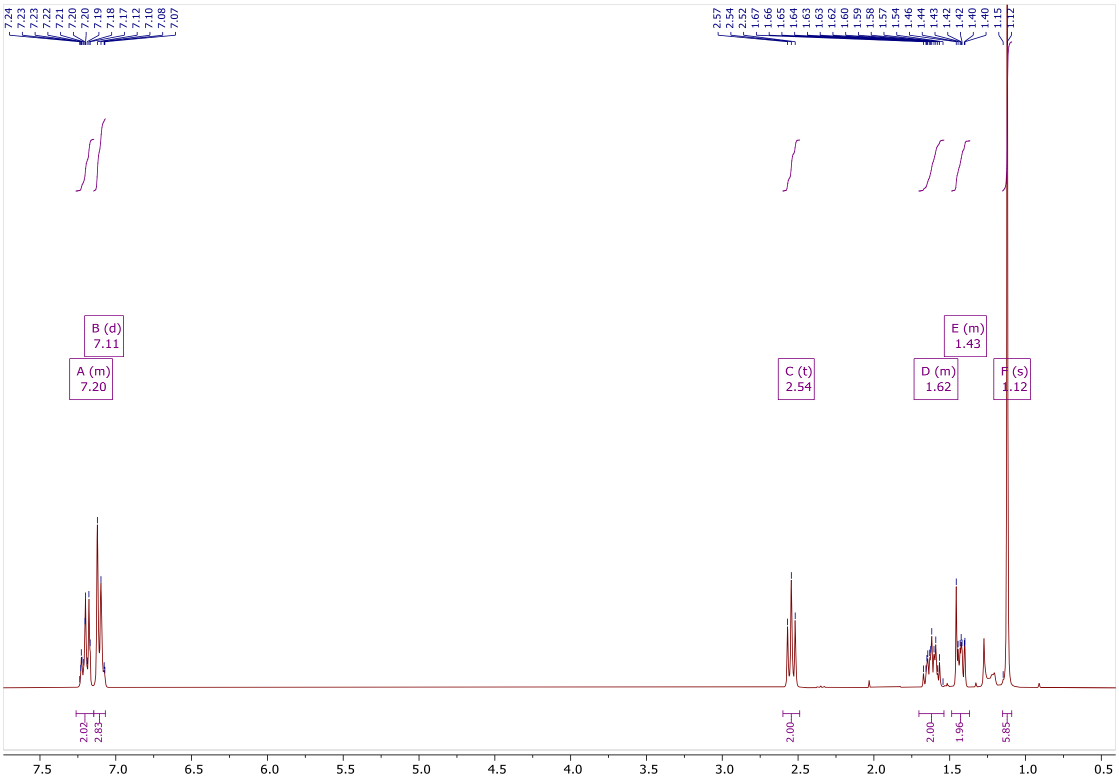

Figure S4: ^{1}H NMR spectrum of 2-methyl-5-phenylpentan-2-ol in $CDCl_3$.

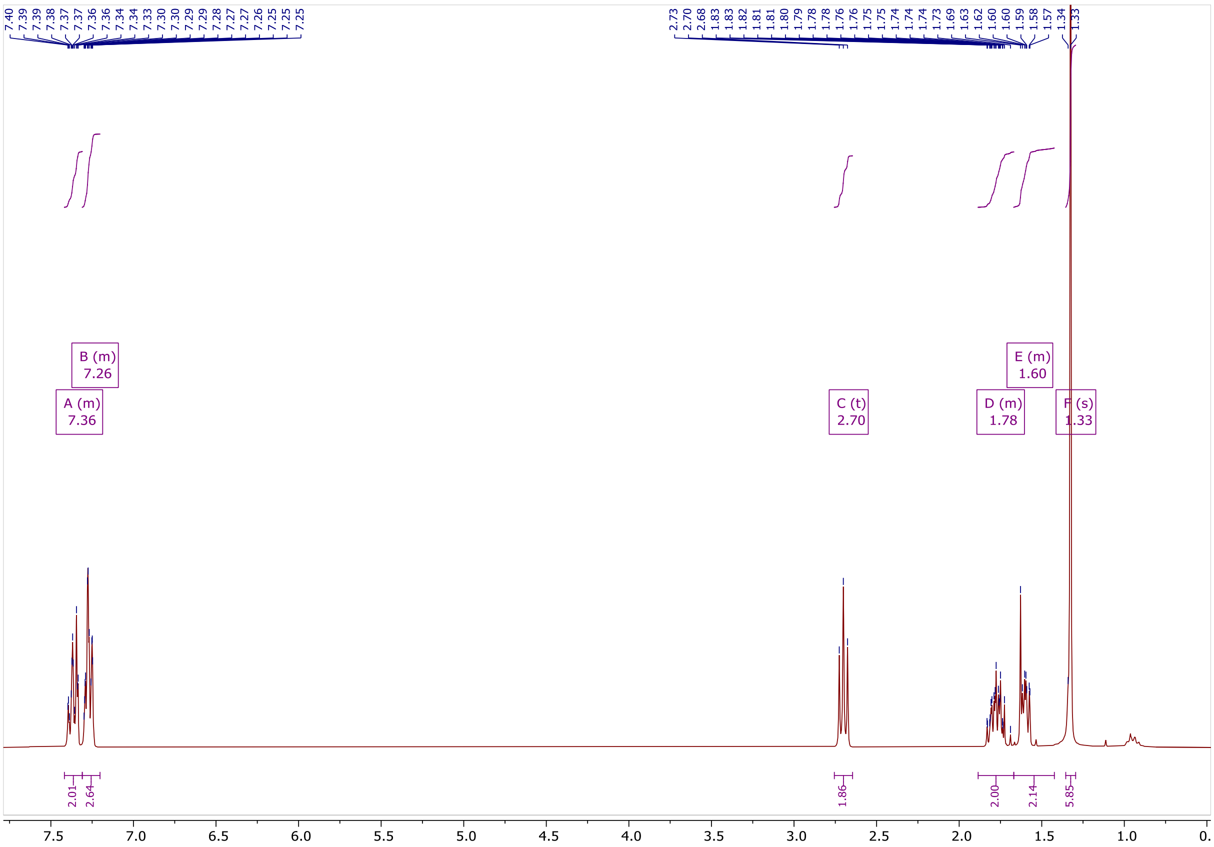

Figure S5: ^{1}H NMR spectrum of substrate **1a** in $CDCl_3$.

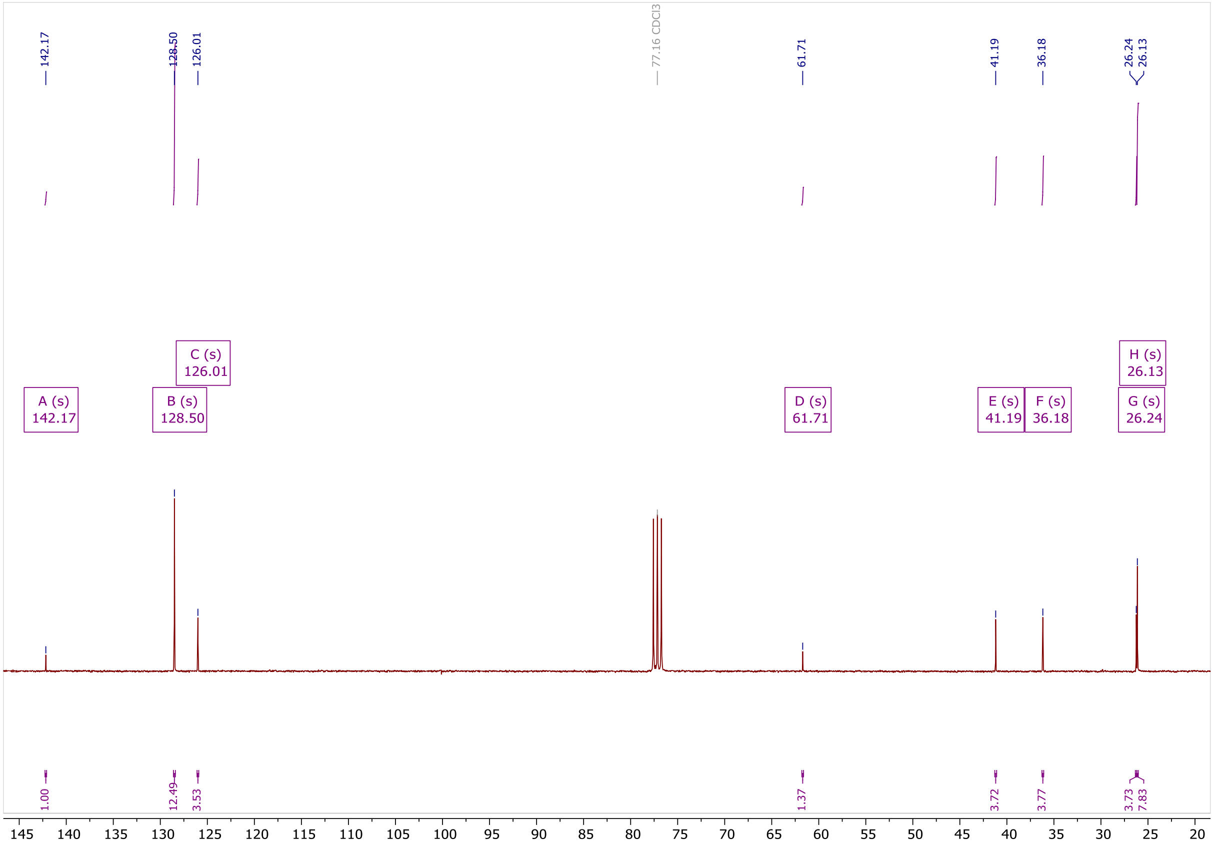

Figure S6: ^{13}C NMR spectrum of substrate **1a** in $CDCl_3$.

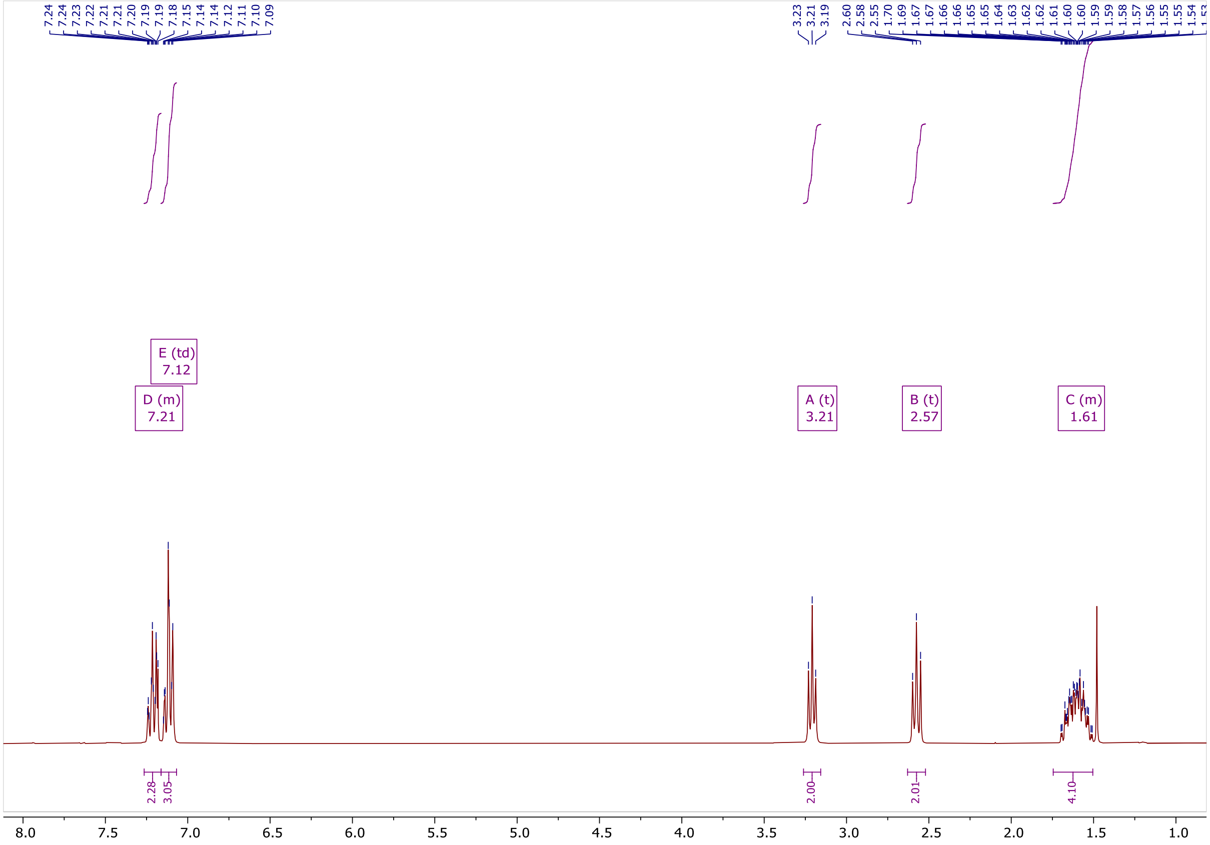

Figure S7: ^{1}H NMR spectrum of **S2** $CDCl_3$.

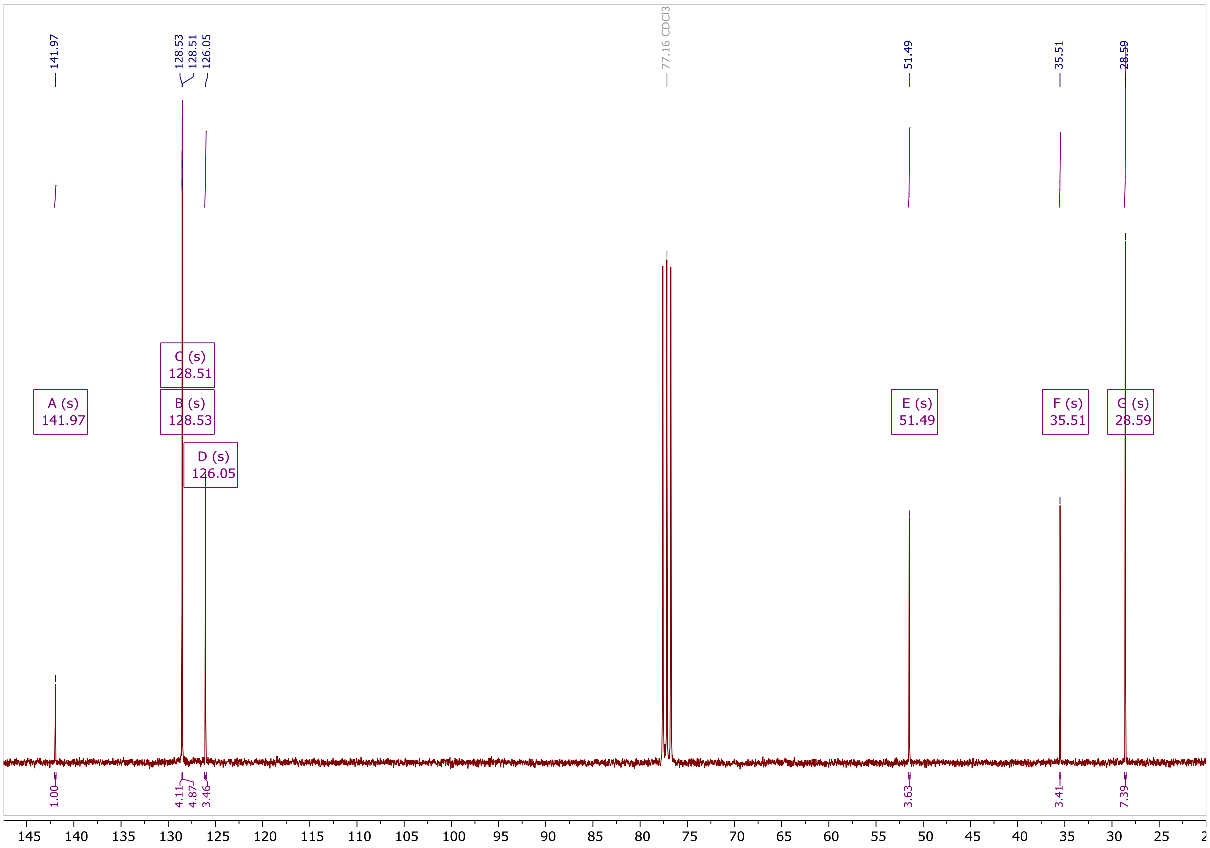

Figure S8: ^{13}C NMR spectrum of **S2** $CDCl_3$.

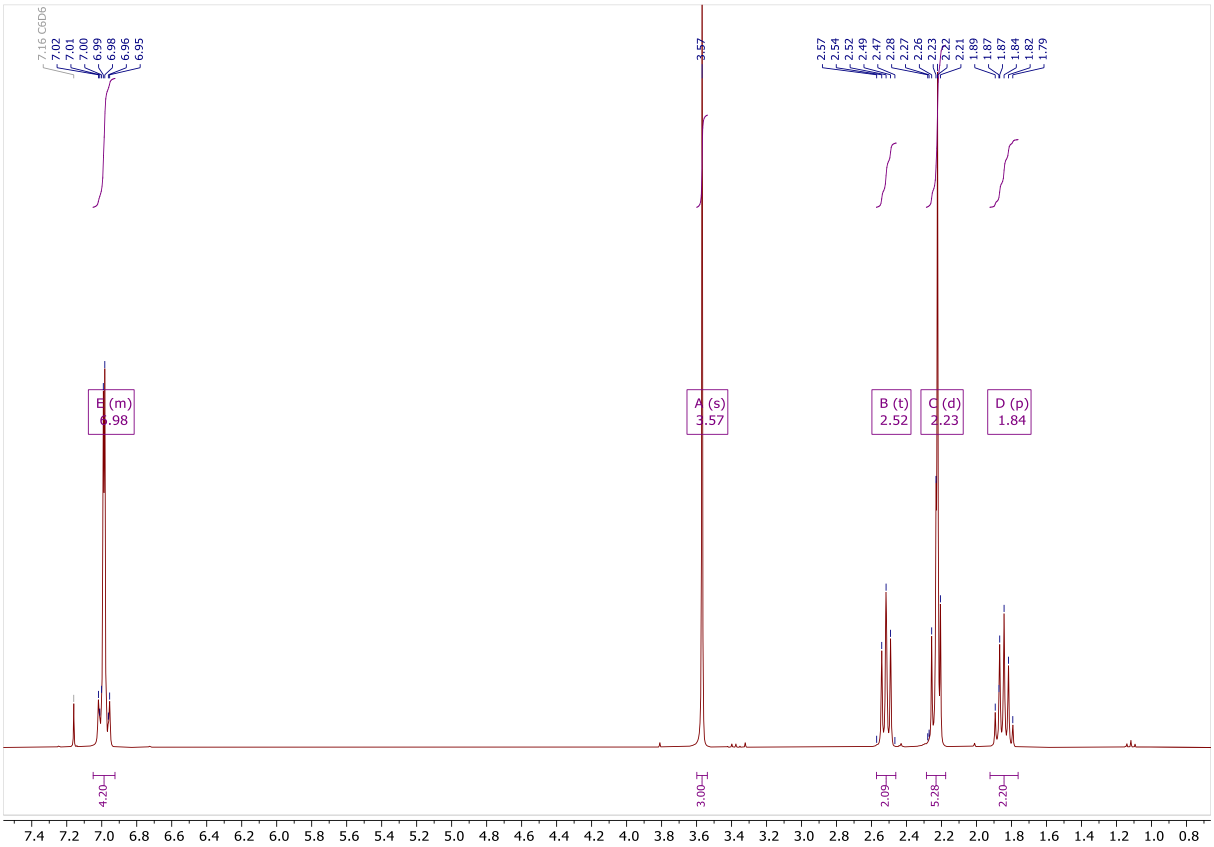

Figure S9: ^{1}H NMR spectrum of 4-(p-tolyl)butanoate in $CDCl_3$.

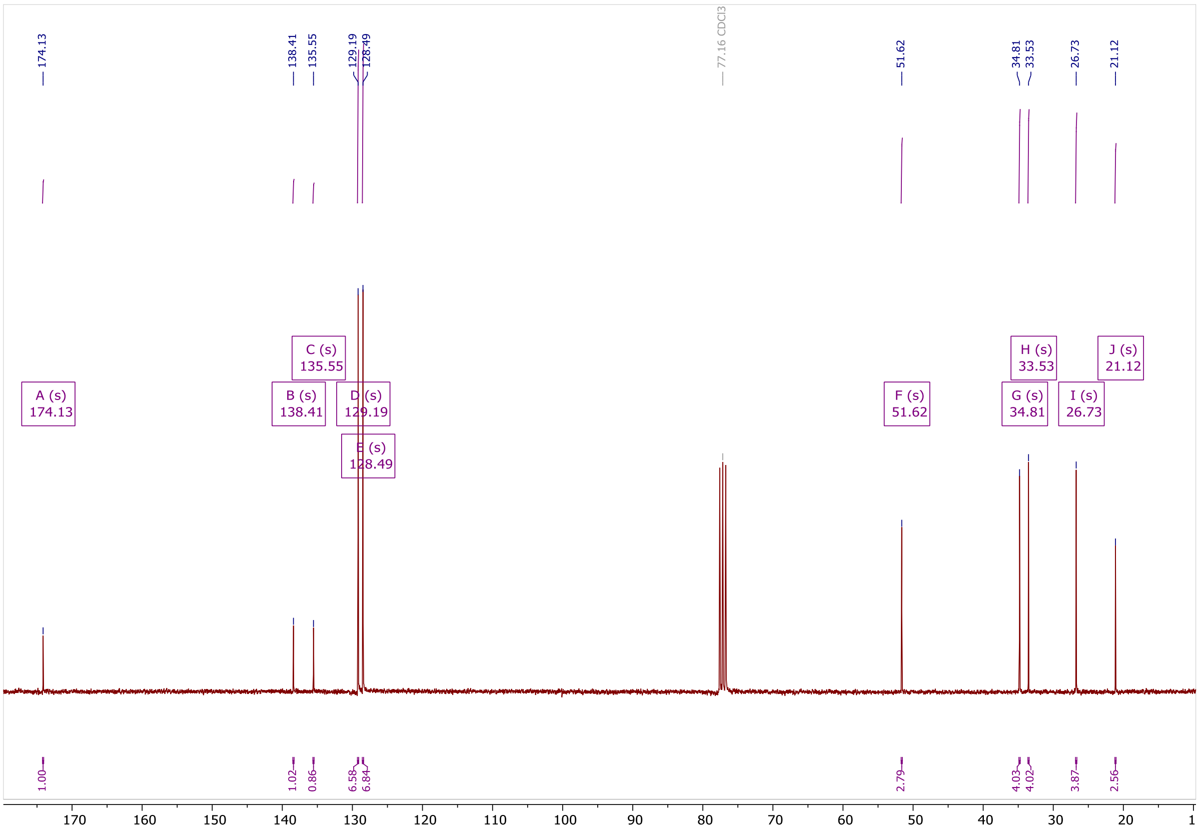

Figure S10: ^{13}C NMR spectrum of 4-(p-tolyl)butanoate in $CDCl_3$.

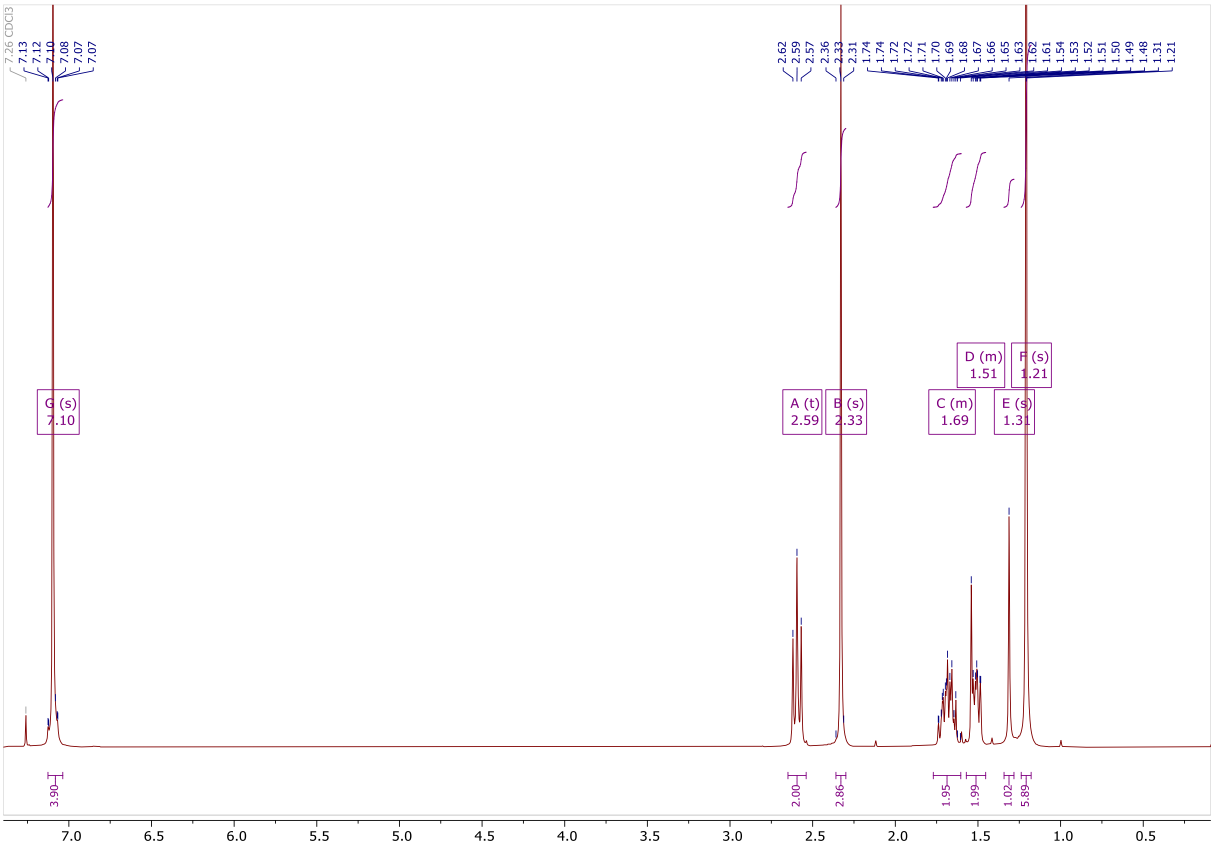

Figure S11: ^{1}H NMR spectrum of 2-methyl-5-(p-tolyl)pentan-2-ol in $CDCl_3$.

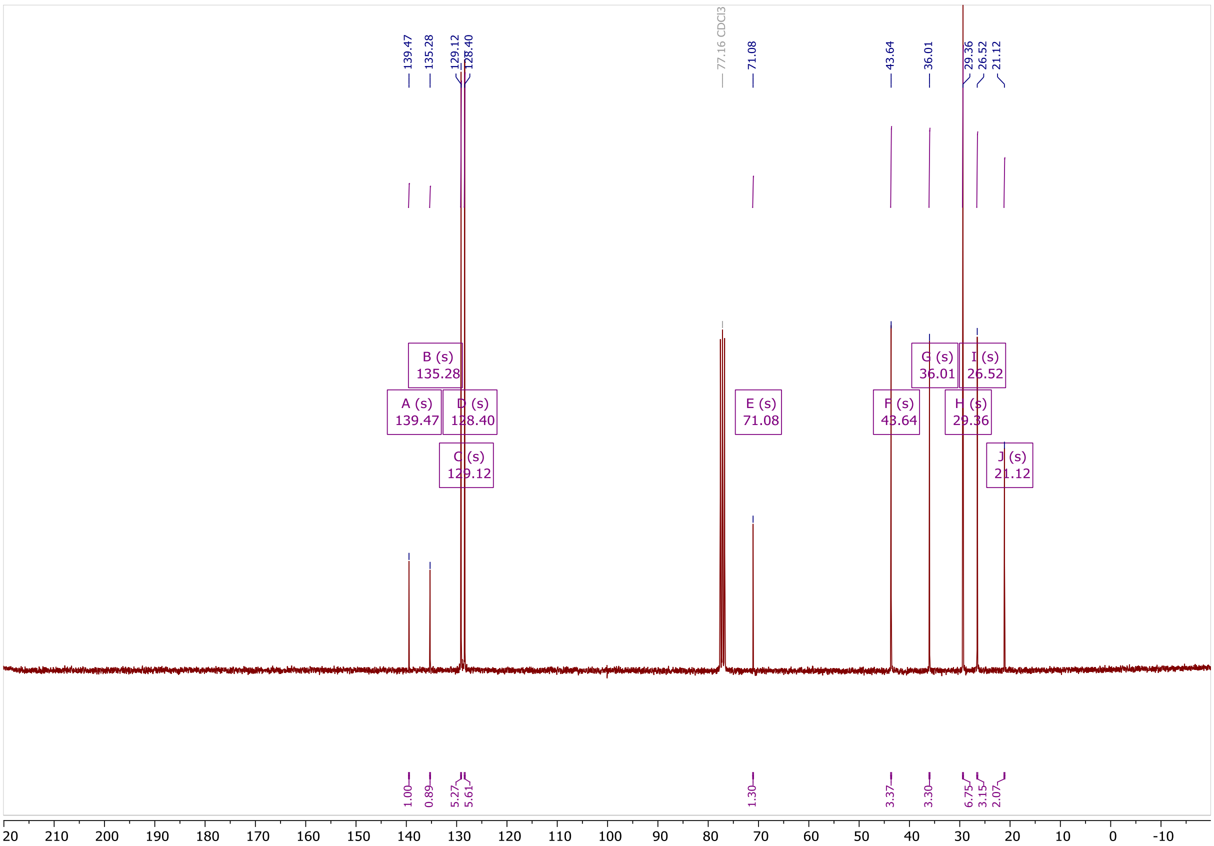

Figure S12: ^{13}C NMR spectrum of 2-methyl-5-(p-tolyl)pentan-2-ol in $CDCl_3$.

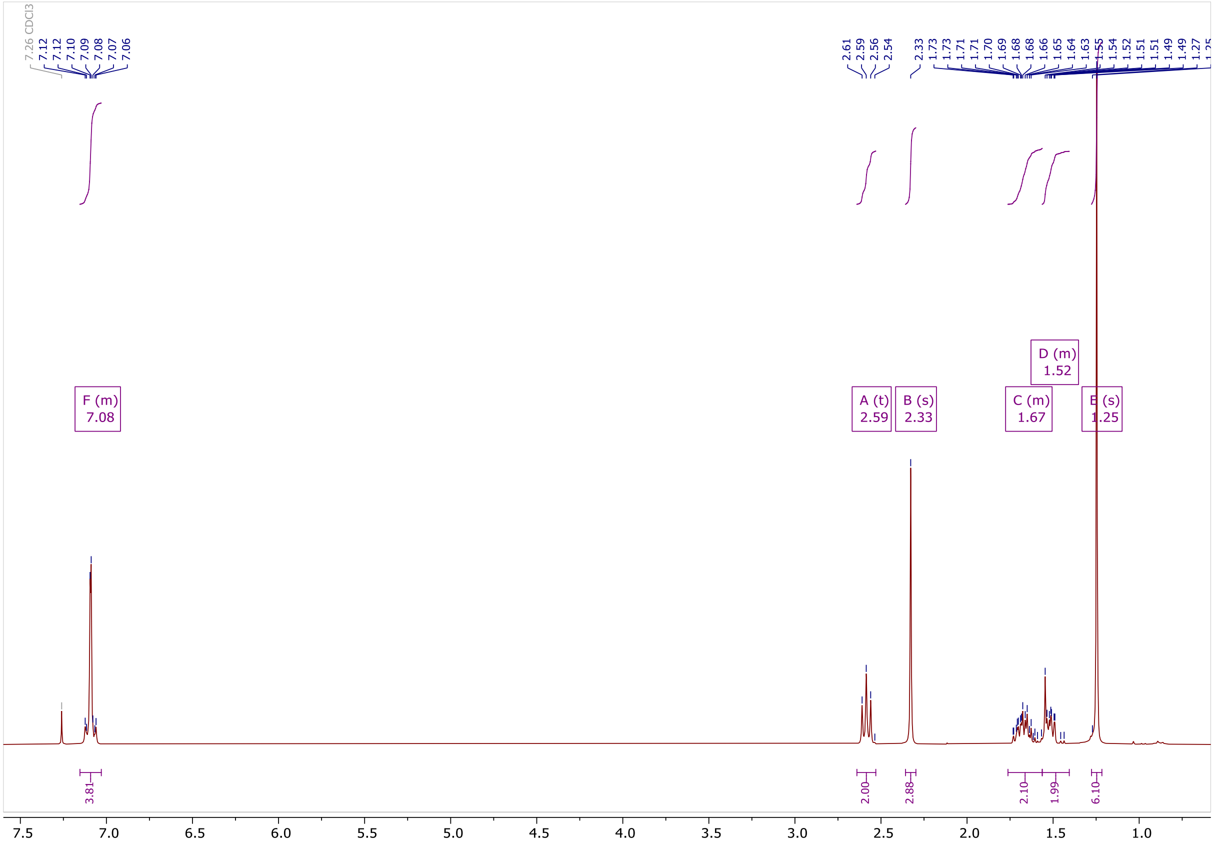

Figure S13: ^{1}H NMR spectrum of substrate **3a** in $CDCl_3$.

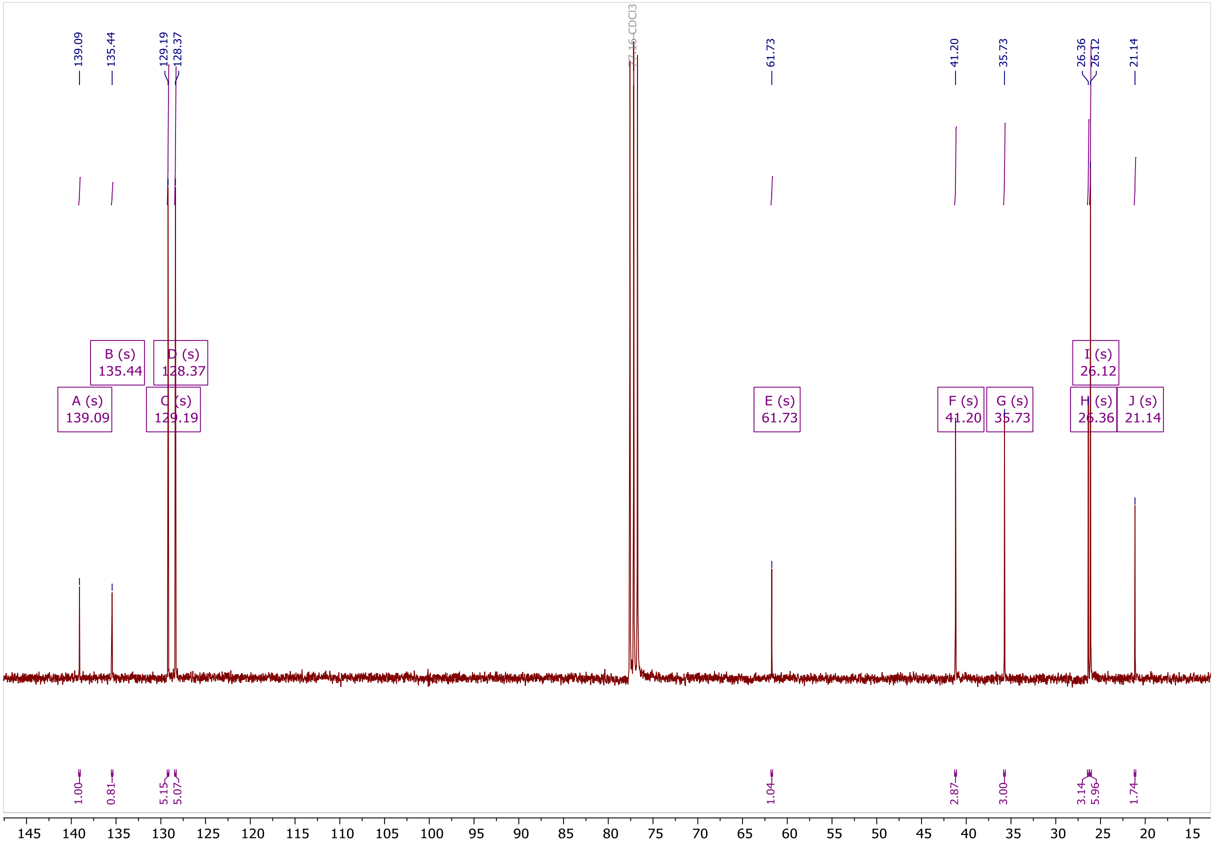

Figure S14: ^{13}C NMR spectrum of substrate **3a** in $CDCl_3$.

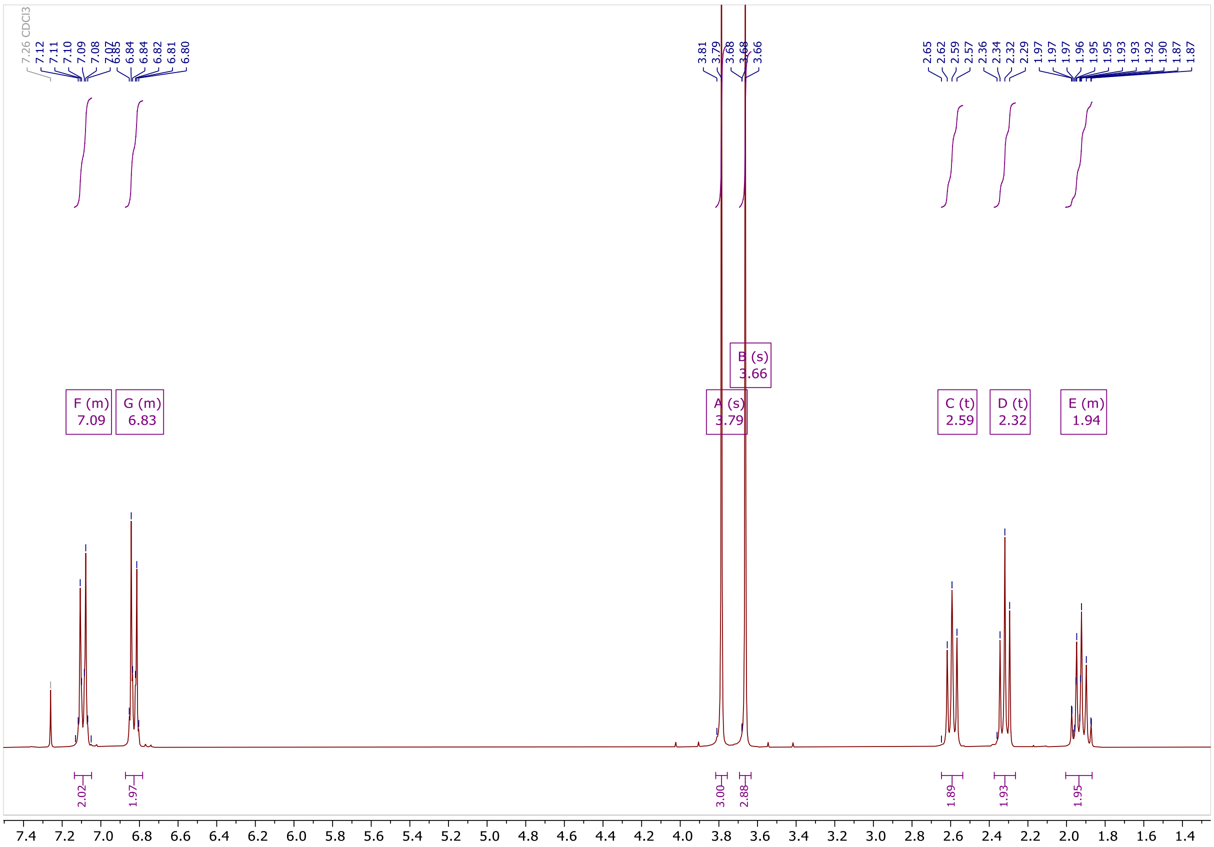

Figure S15: ^{1}H NMR spectrum of methyl 4-(4-methoxyphenyl)butanoate in $CDCl_3$.

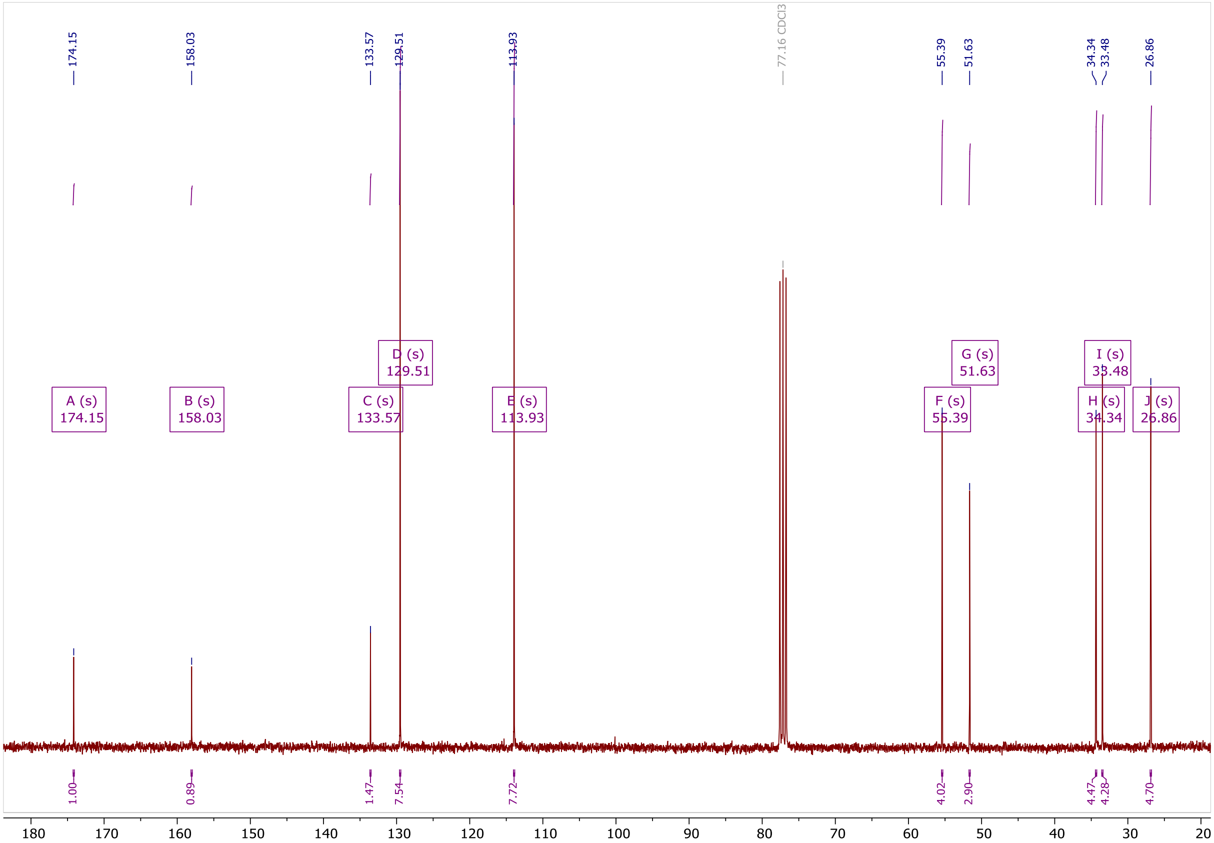

Figure S16: ^{13}C NMR spectrum of methyl 4-(4-methoxyphenyl)butanoate in $CDCl_3$.

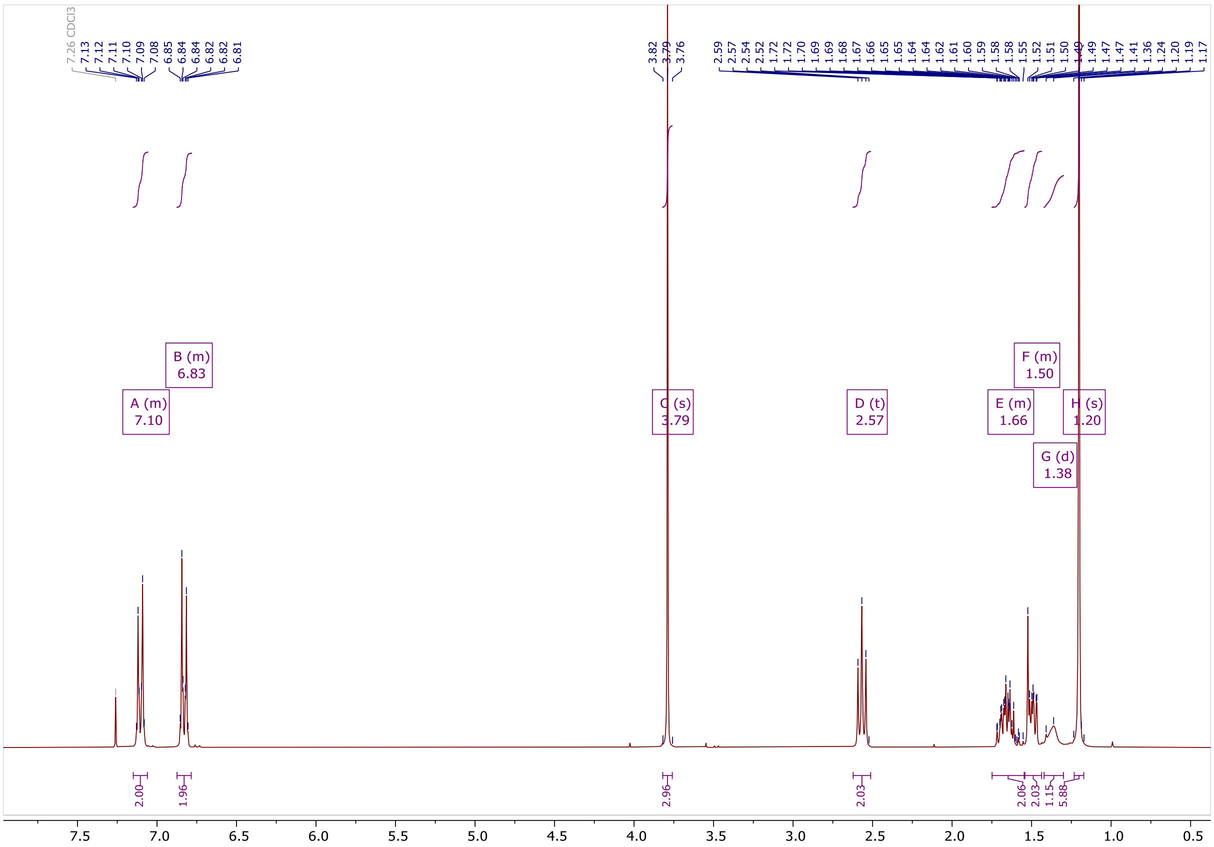

Figure S17: ^{1}H NMR spectrum of 5-(4-methoxyphenyl)-2-methylpentan-2-ol in $CDCl_3$.

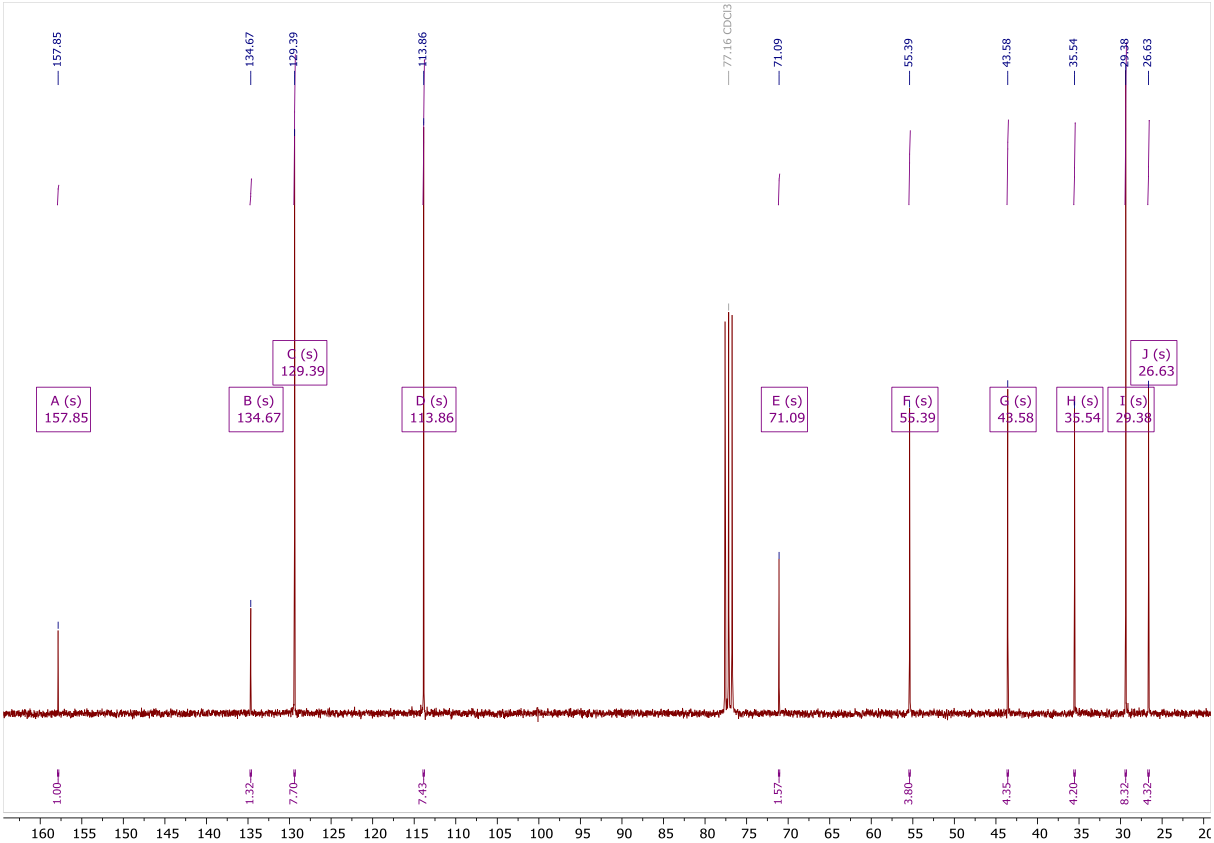

Figure S18: ^{13}C NMR spectrum of 5-(4-methoxyphenyl)-2-methylpentan-2-ol in $CDCl_3$.

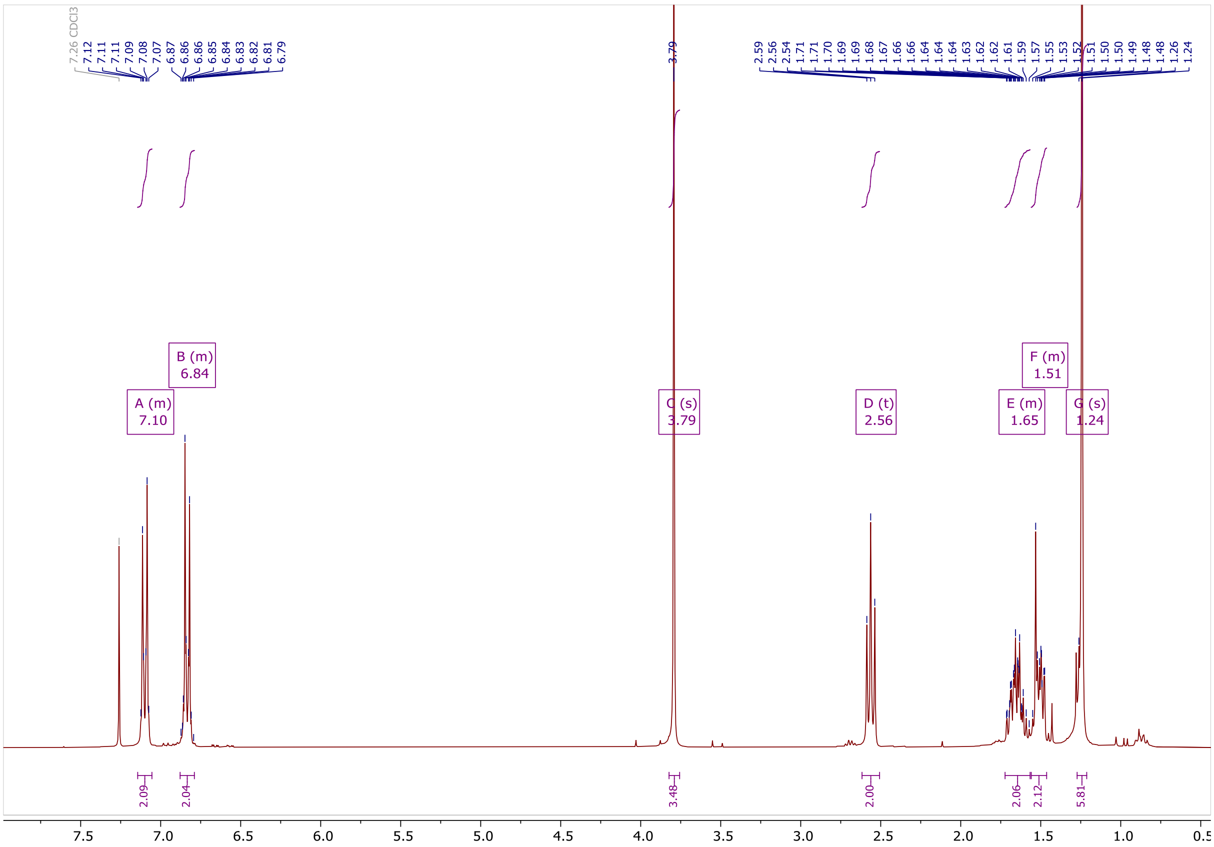

Figure S19: ^{1}H NMR spectrum of substrate **4a** in $CDCl_3$.

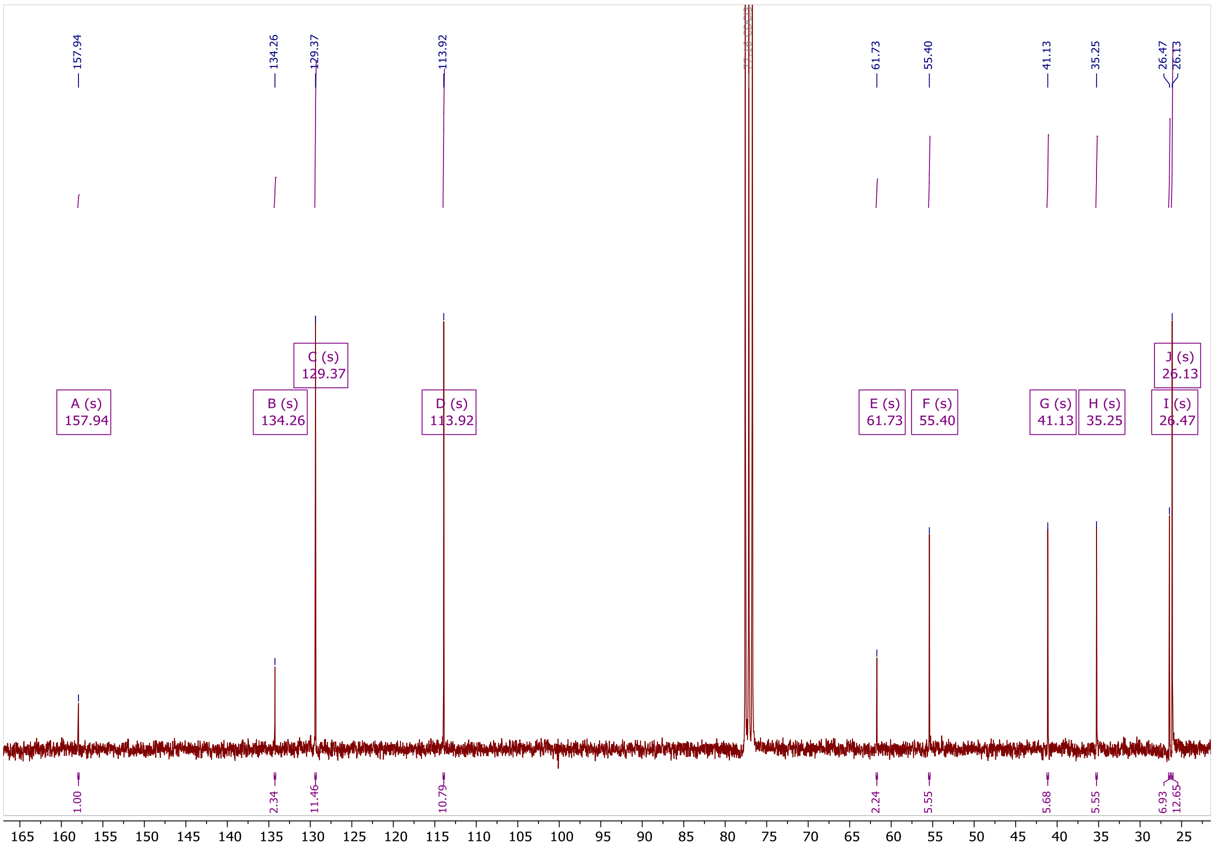

Figure S20: ^{13}C NMR spectrum of substrate **4a** in $CDCl_3$.

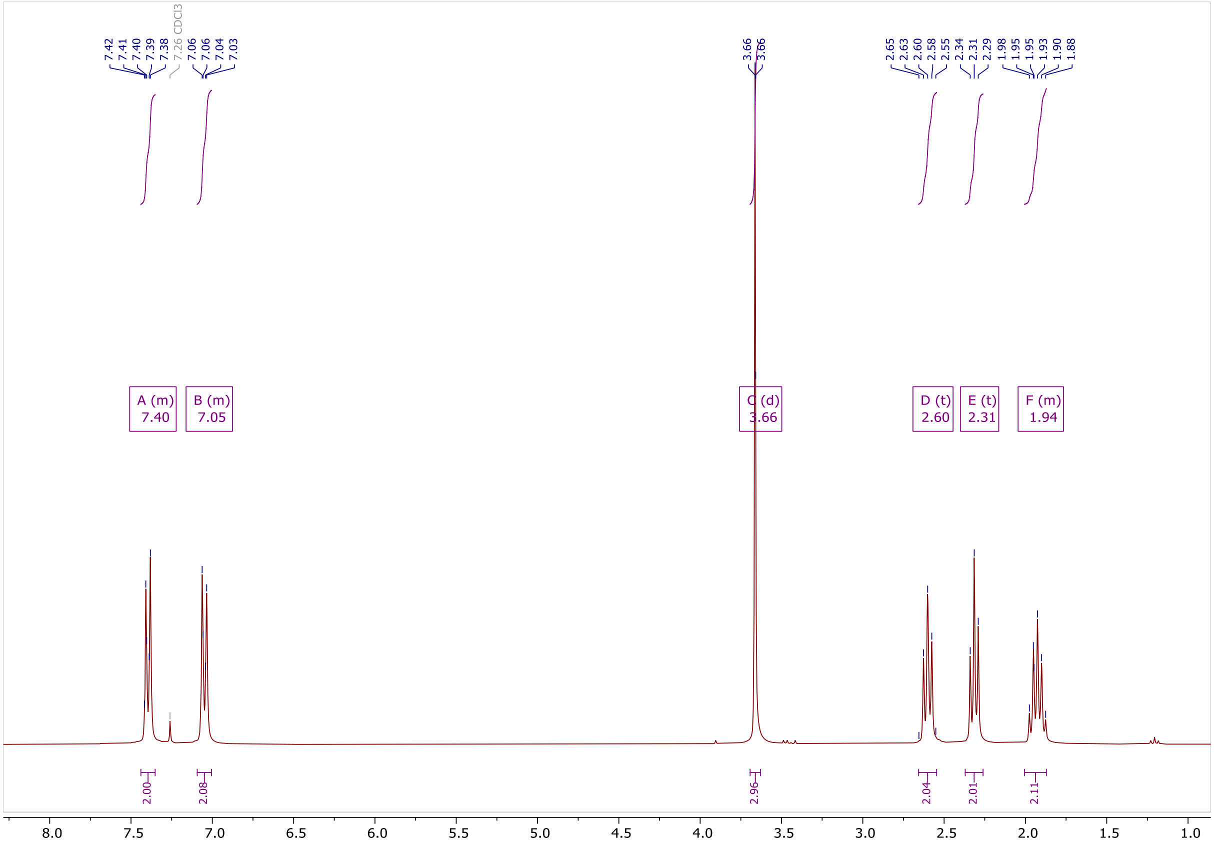

Figure S21: ^{1}H NMR spectrum of methyl 4-(4-bromophenyl)butanoate in $CDCl_3$.

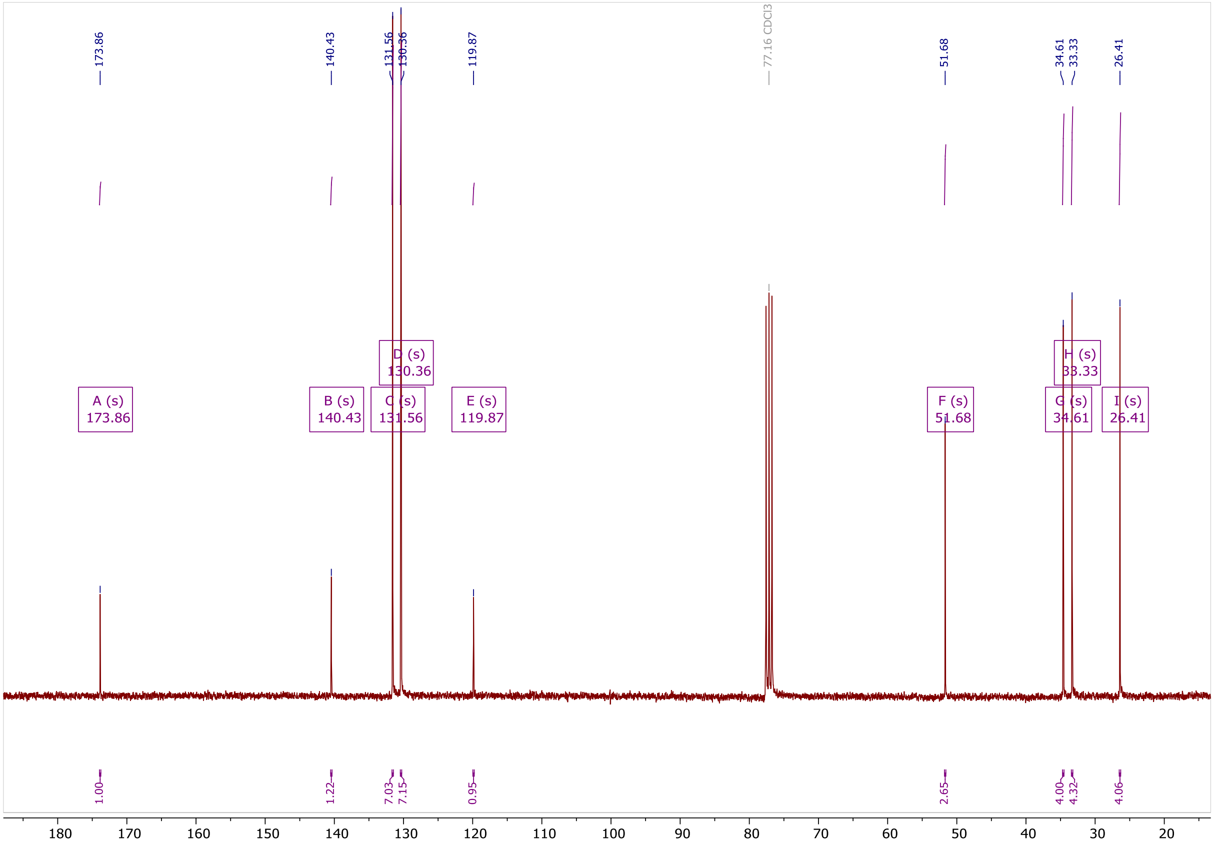

Figure S22: ^{13}C NMR spectrum of methyl 4-(4-bromophenyl)butanoate in $CDCl_3$.

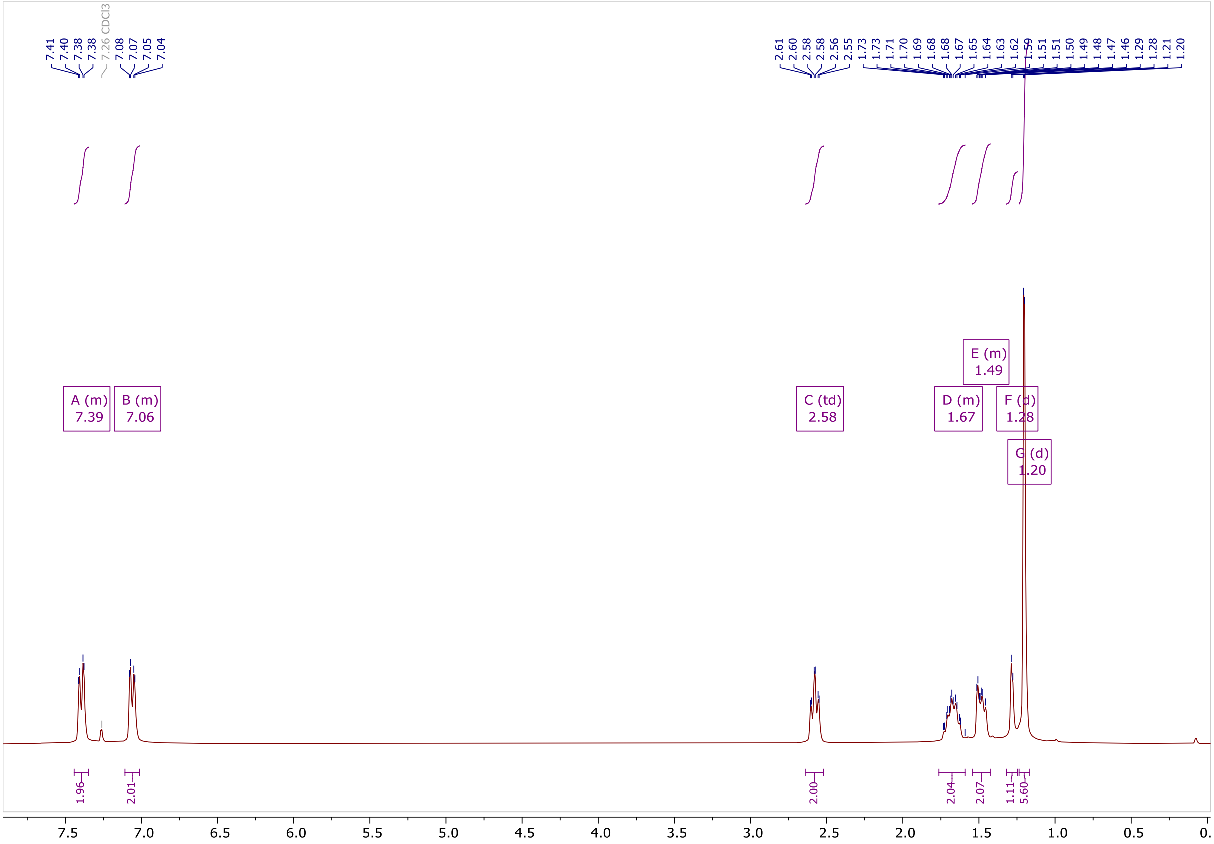

Figure S23: ^{1}H NMR spectrum of 5-(4-bromophenyl)-2-methylpentan-2-ol in $CDCl_3$.

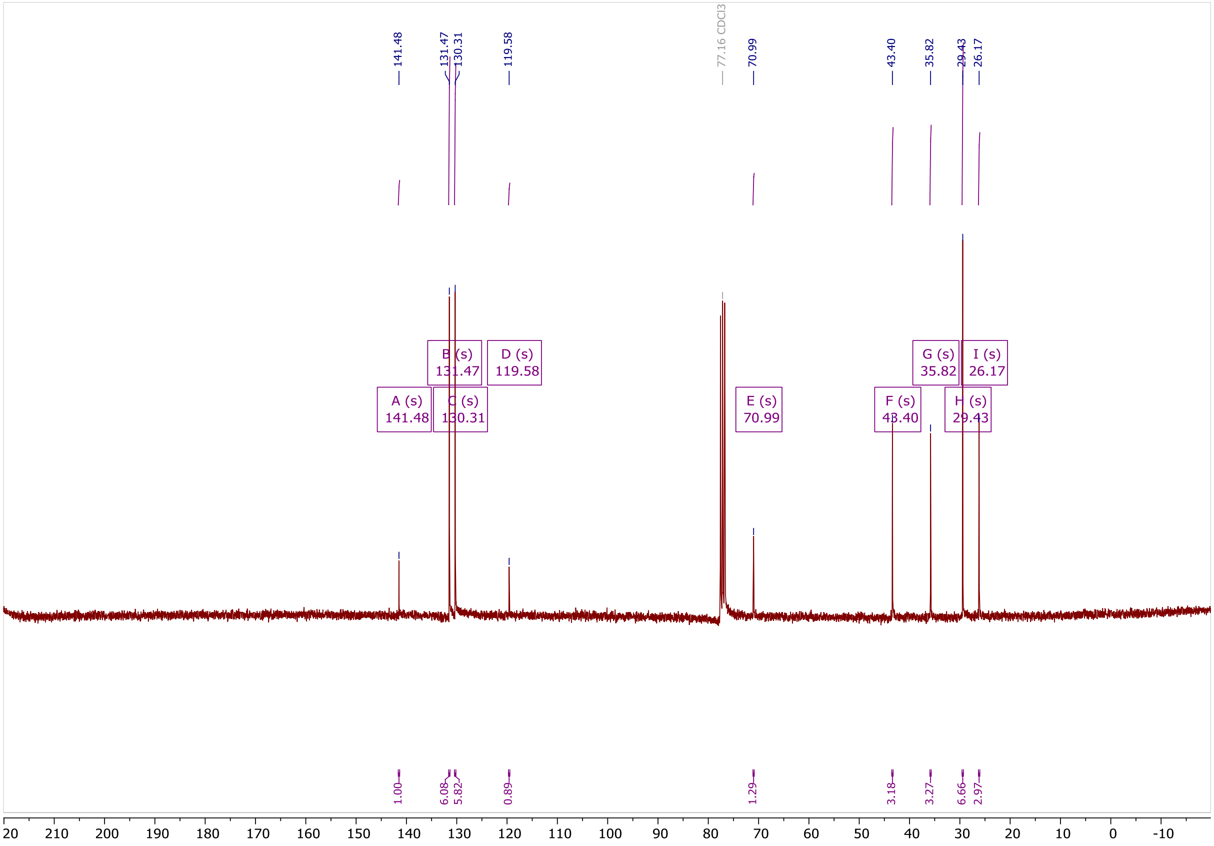

Figure S24: ^{13}C NMR spectrum of 5-(4-bromophenyl)-2-methylpentan-2-ol in $CDCl_3$.

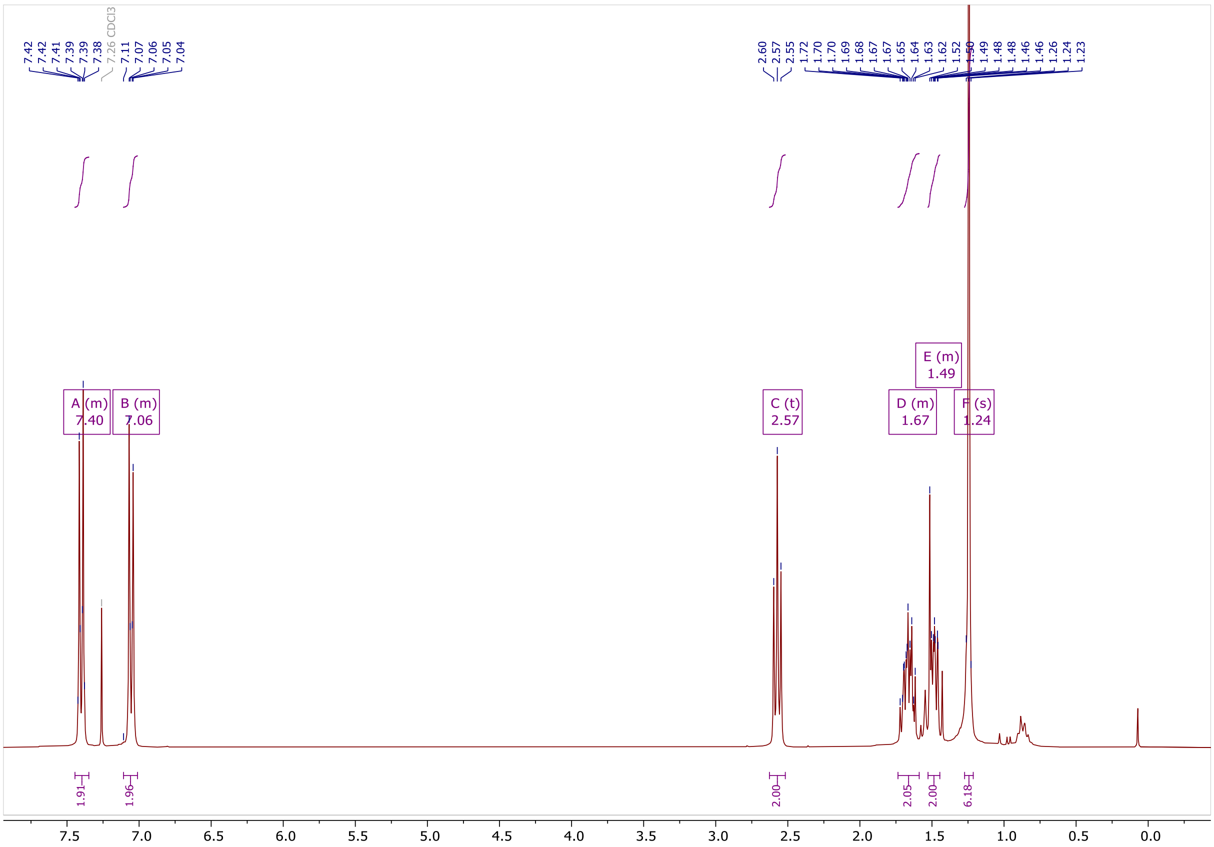

Figure S25: ^{1}H NMR spectrum of substrate **5a** in $CDCl_3$.

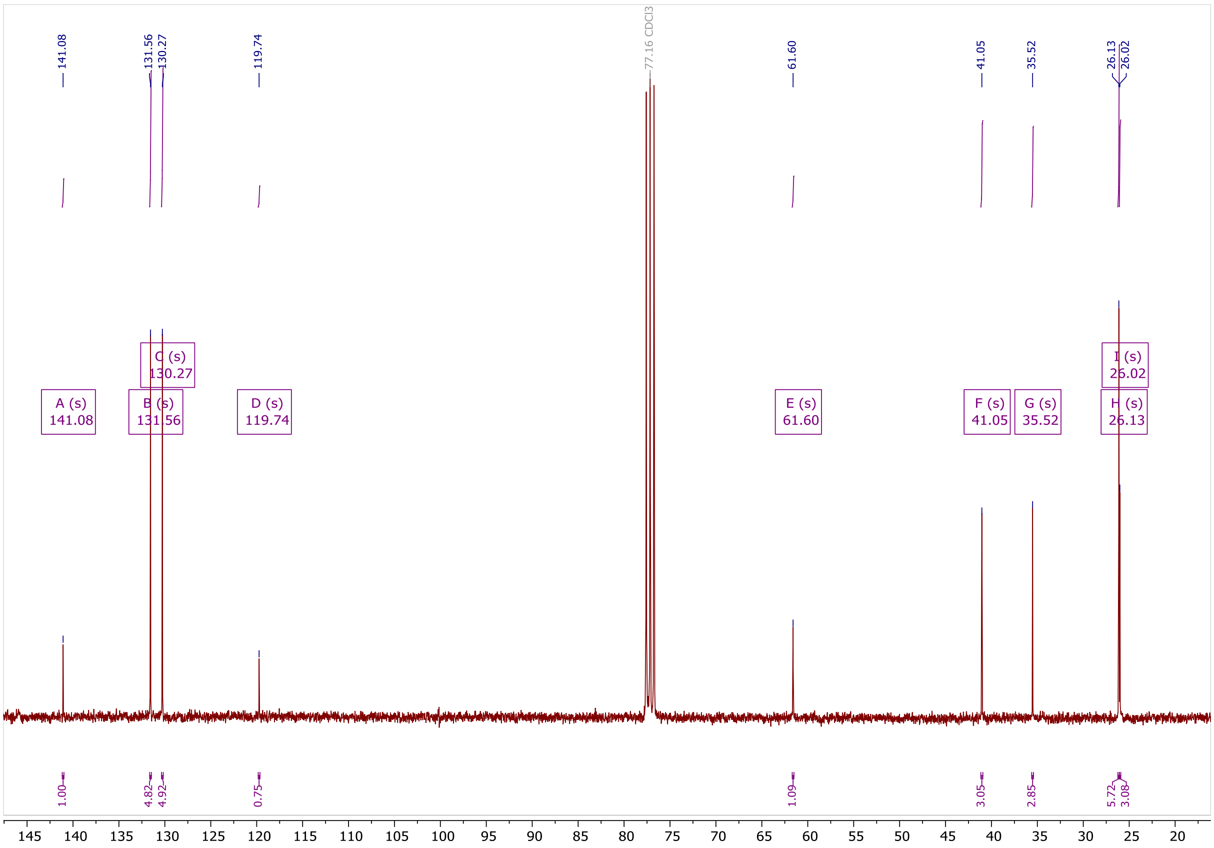

Figure S26: ^{13}C NMR spectrum of substrate **5a** in $CDCl_3$.

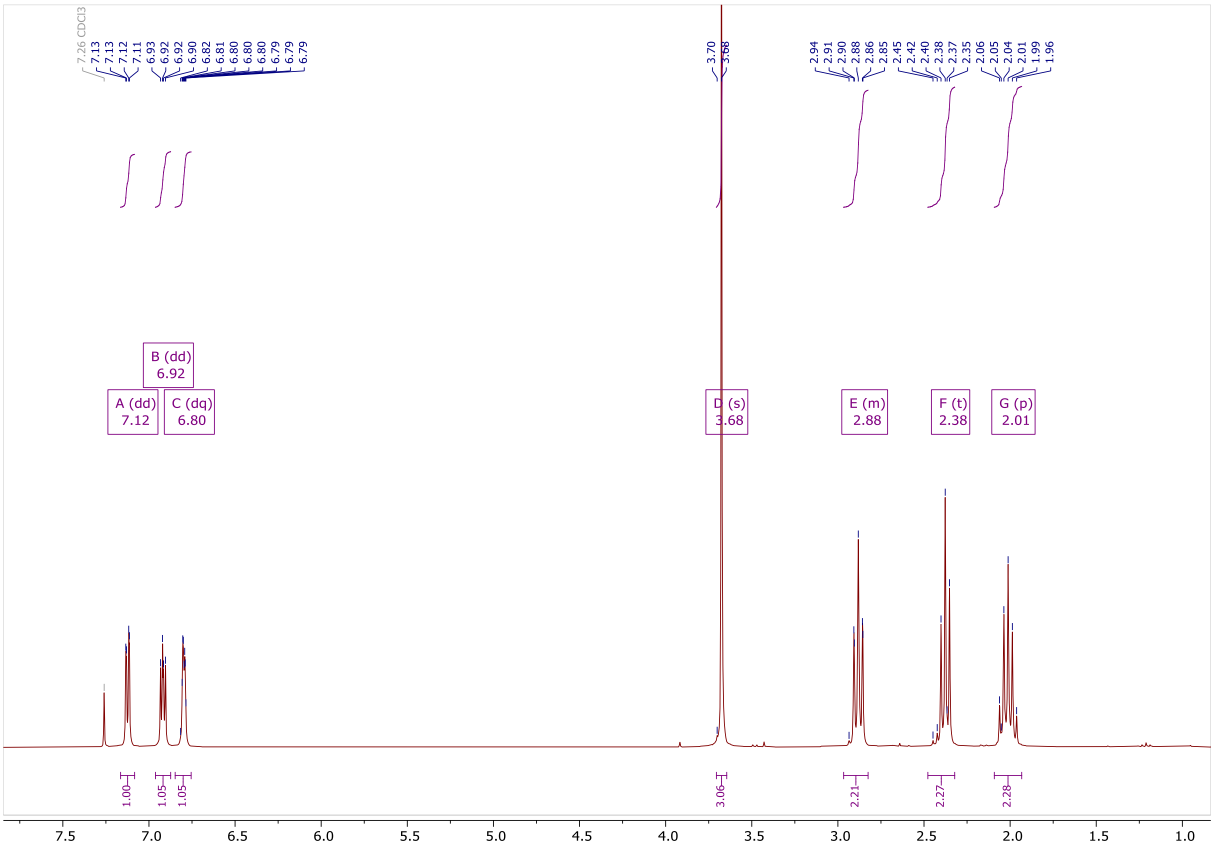

Figure S27: ^1H NMR spectrum of methyl 4-(thiophen-2-yl)butanoate in $CDCl_3$.

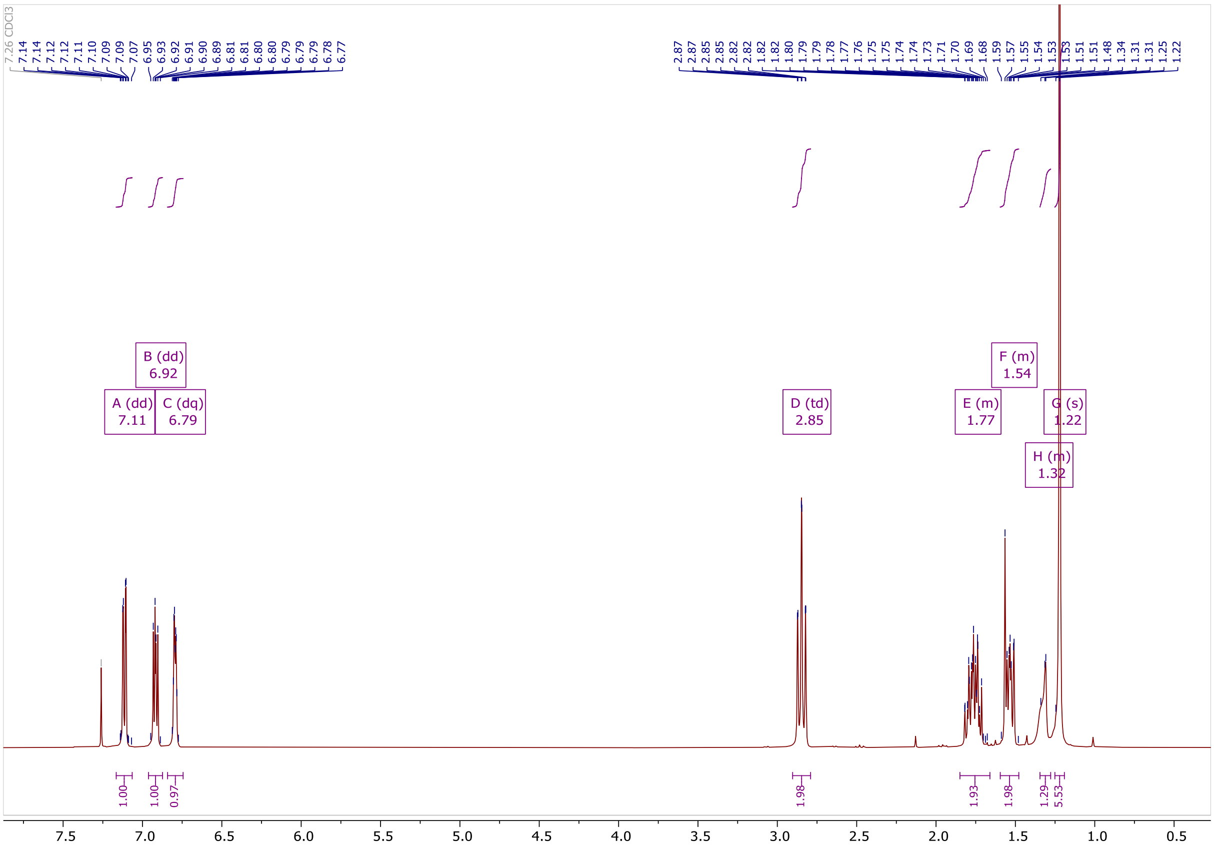

Figure S28: ^1H NMR spectrum of 2-methyl-5-(thiophen-2-yl)pentan-2-ol in $CDCl_3$.

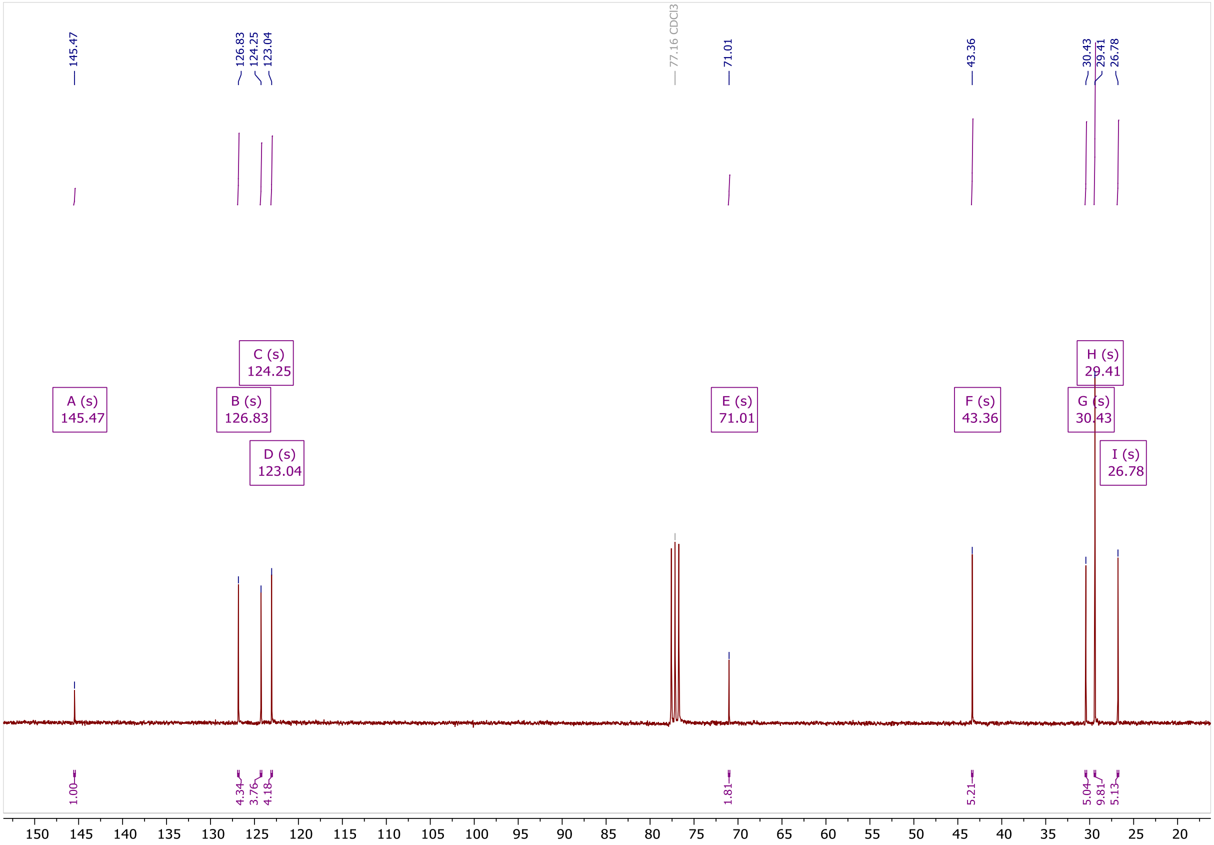

Figure S29: ^{13}C NMR spectrum of 2-methyl-5-(thiophen-2-yl)pentan-2-ol in $CDCl_3$.

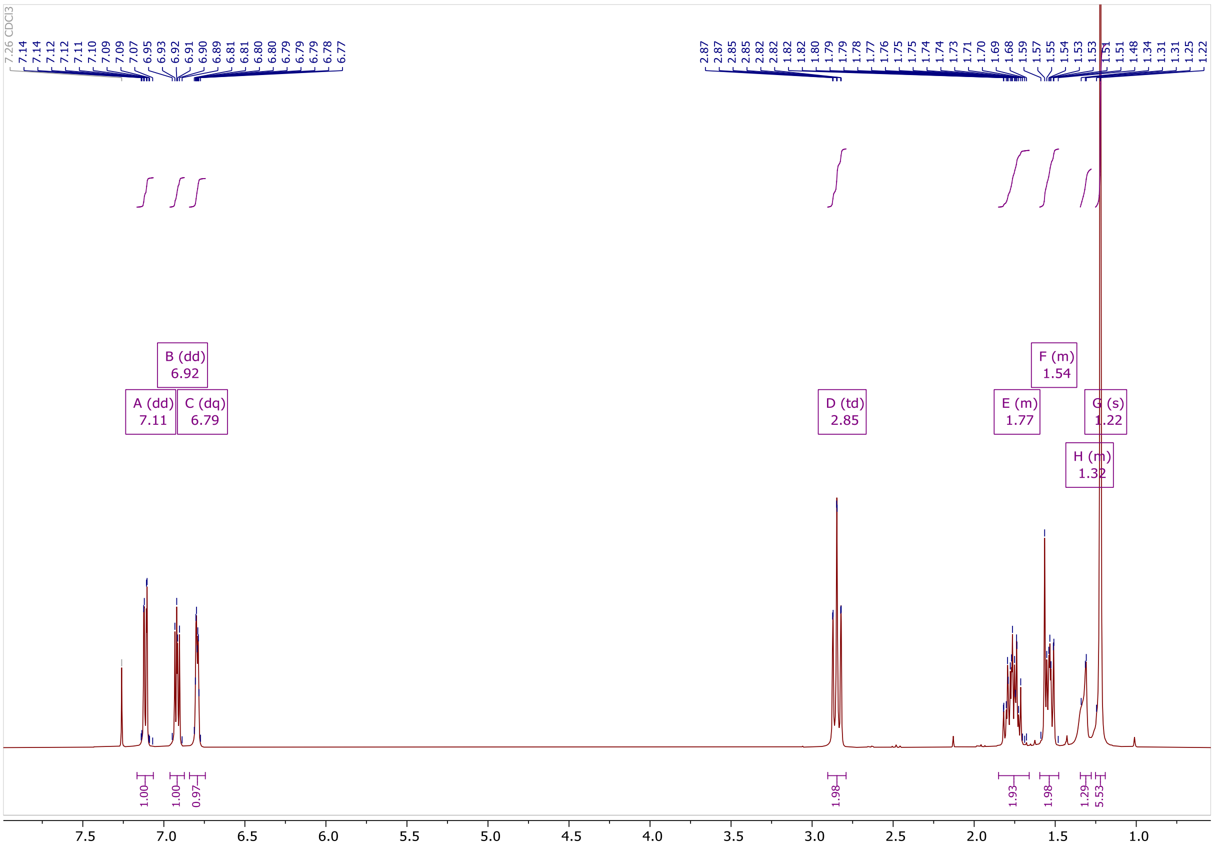

Figure S30: ^{1}H NMR spectrum of substrate **6a** in $CDCl_3$.

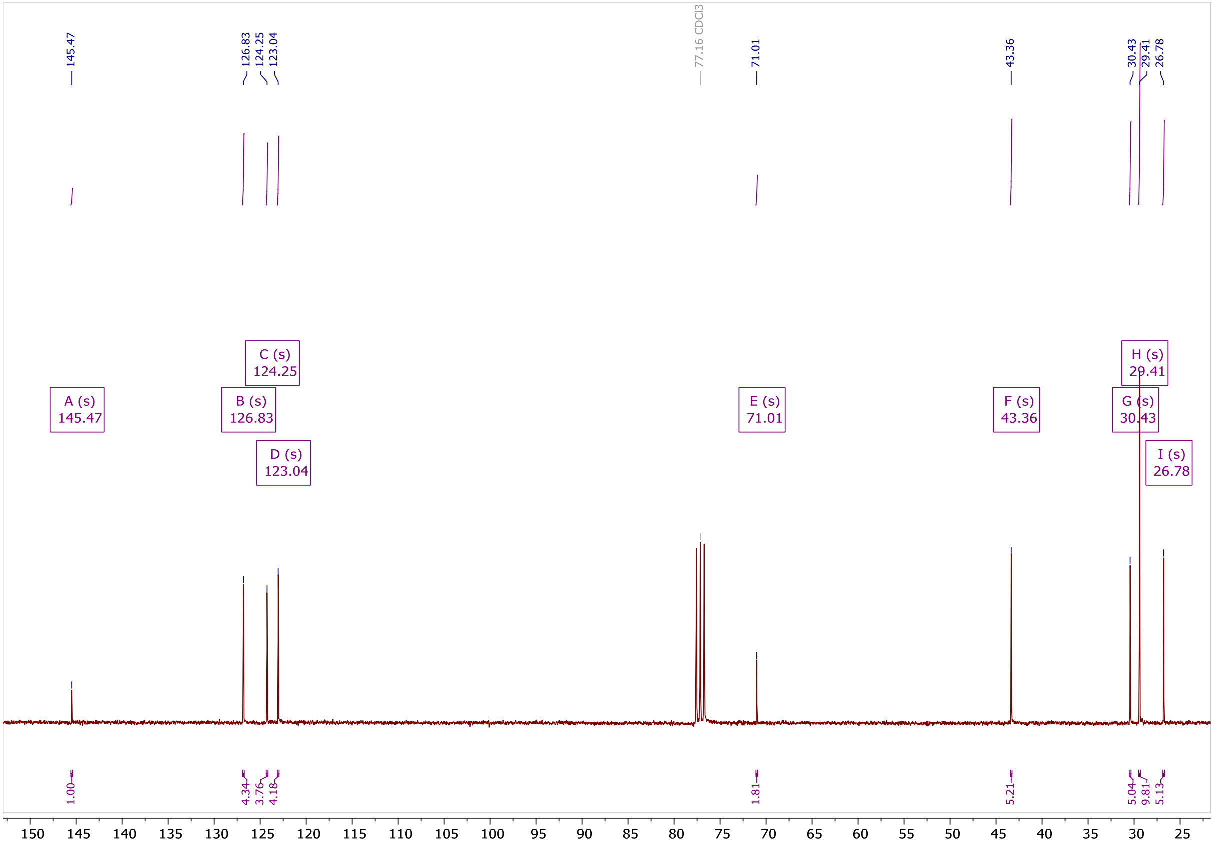

Figure S31: ^{13}C NMR spectrum of substrate **6a** in $CDCl_3$.

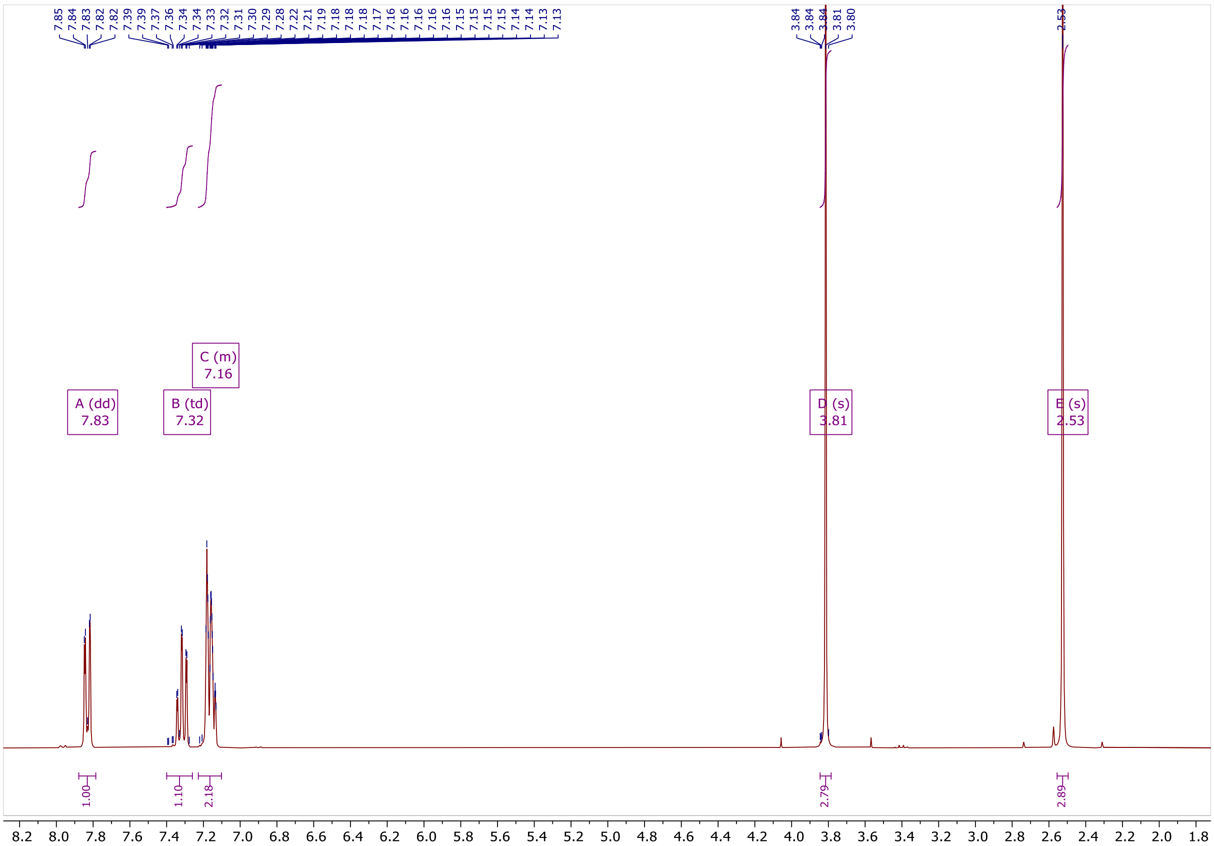

Figure S32: ^{1}H NMR spectrum of methyl 2-methylbenzoate in $CDCl_3$.

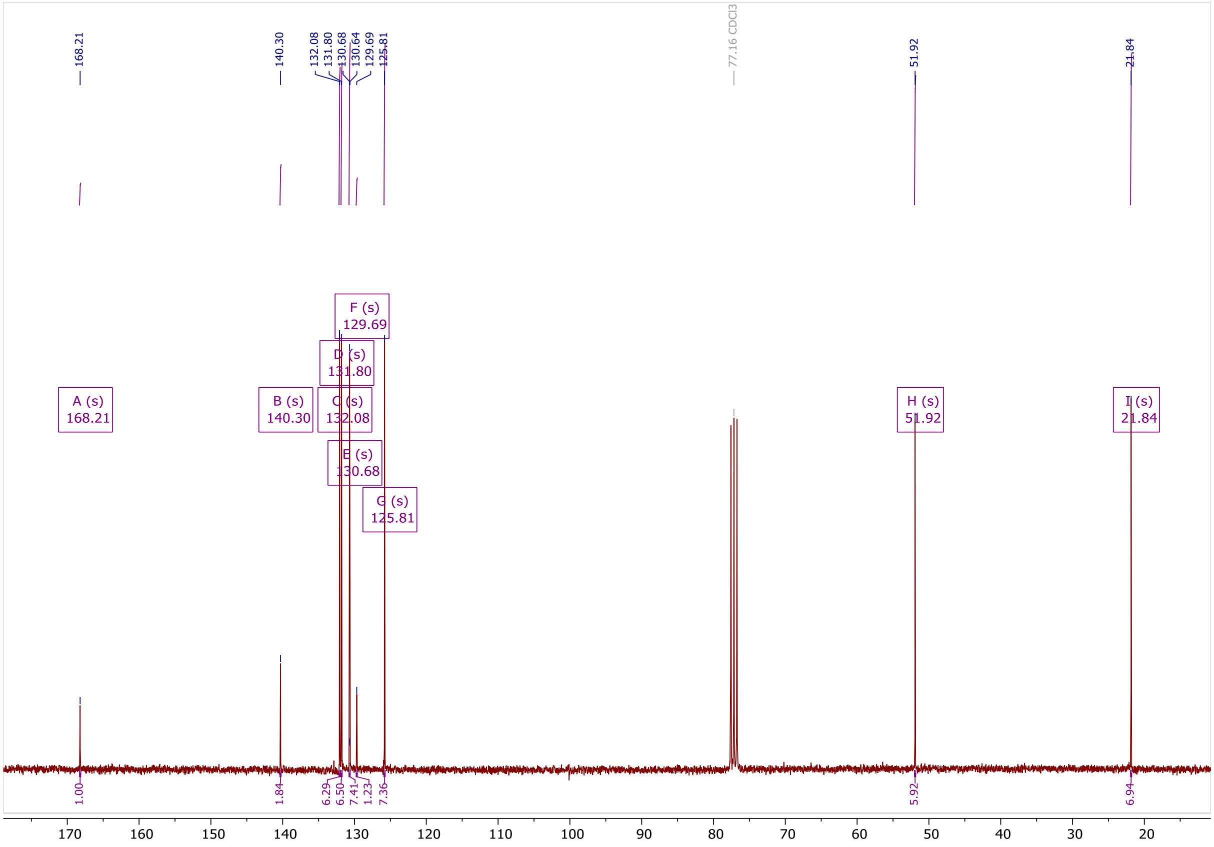

Figure S33: ^{13}C NMR spectrum of methyl 2-methylbenzoate in $CDCl_3$.

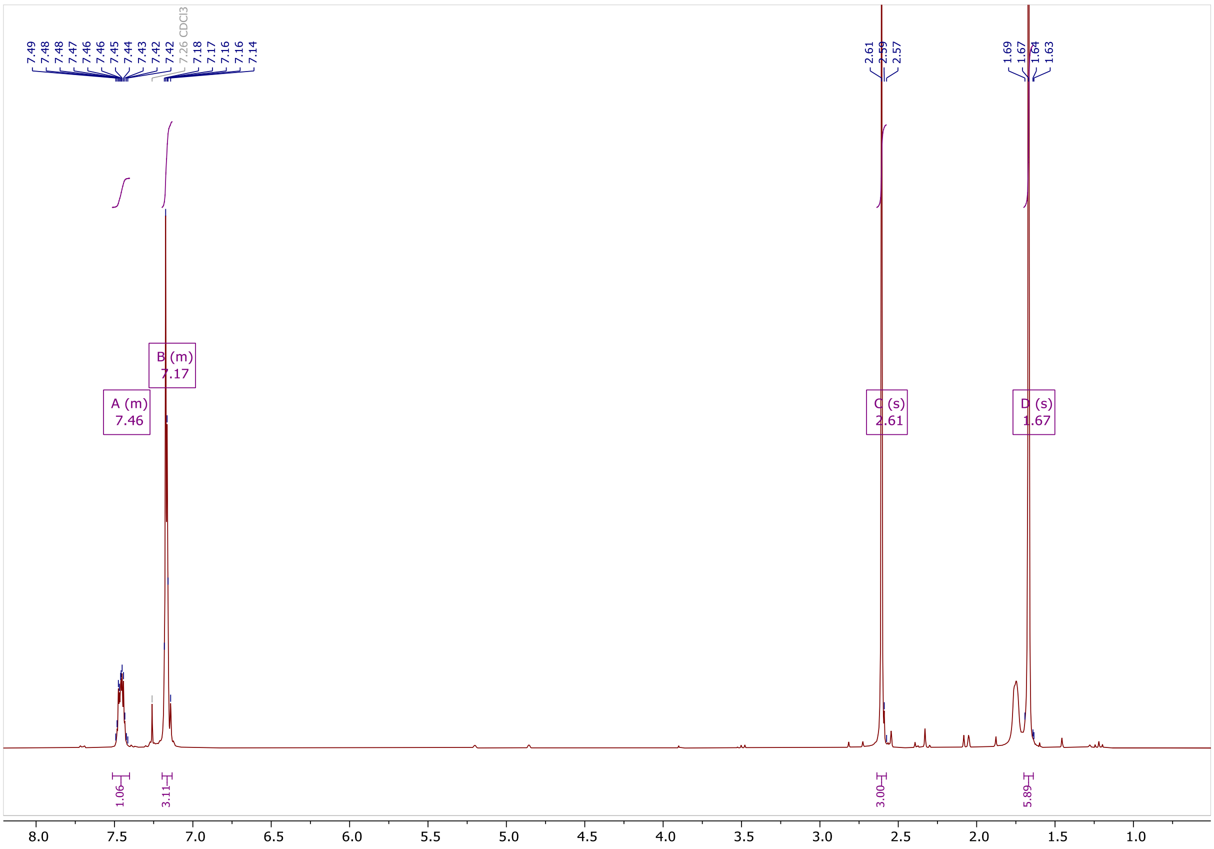

Figure S34: ^{1}H NMR spectrum of 2-(o-tolyl)propan-2-ol in $CDCl_3$.

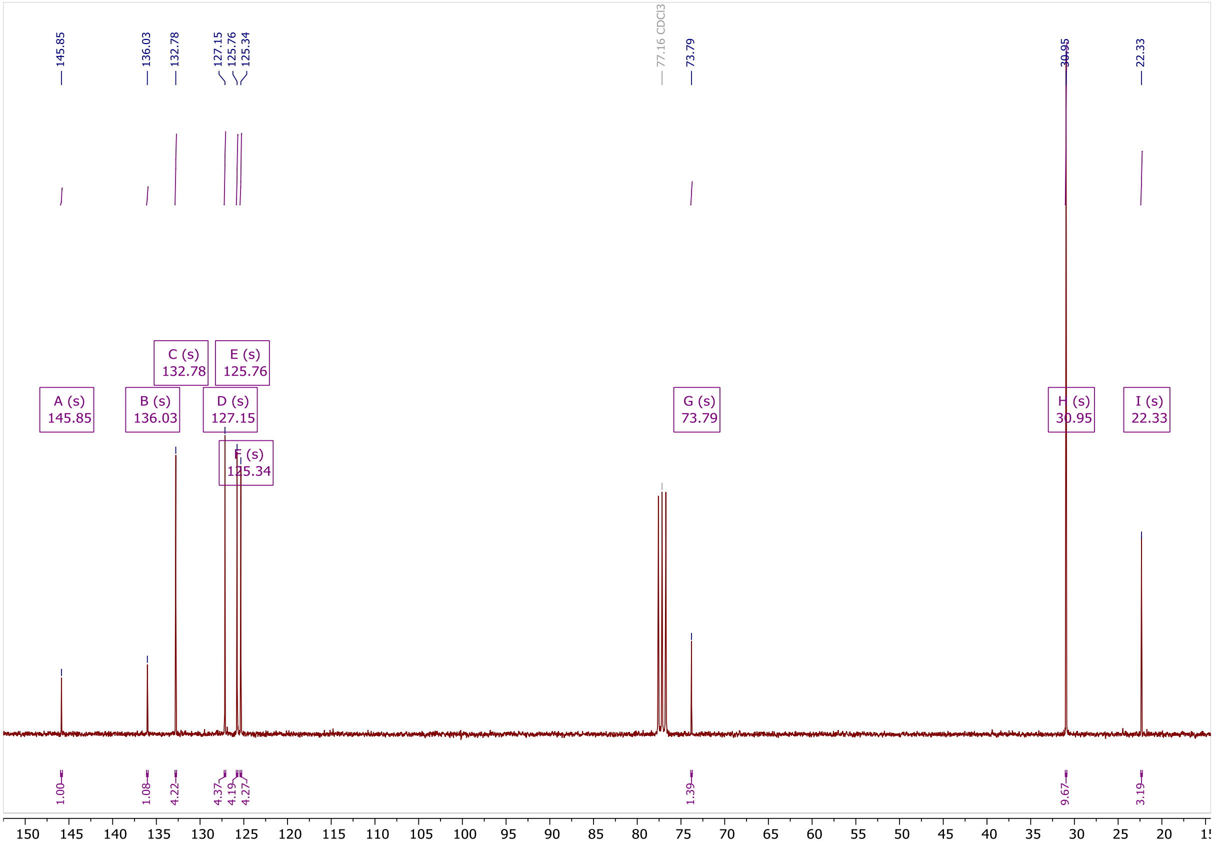

Figure S35: ^{13}C NMR spectrum of 2-(o-tolyl)propan-2-ol in $CDCl_3$.

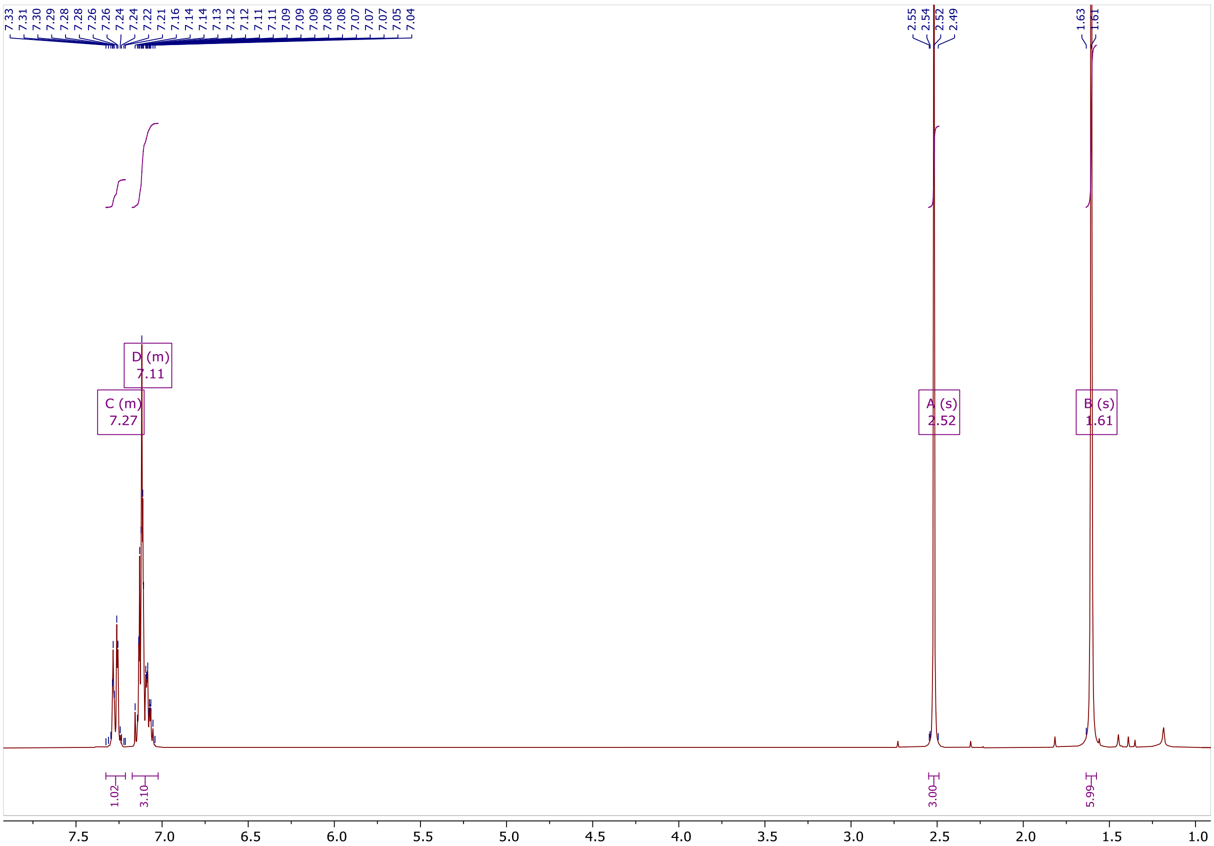

Figure S36: ^{1}H NMR spectrum of substrate **7a** in $CDCl_3$.

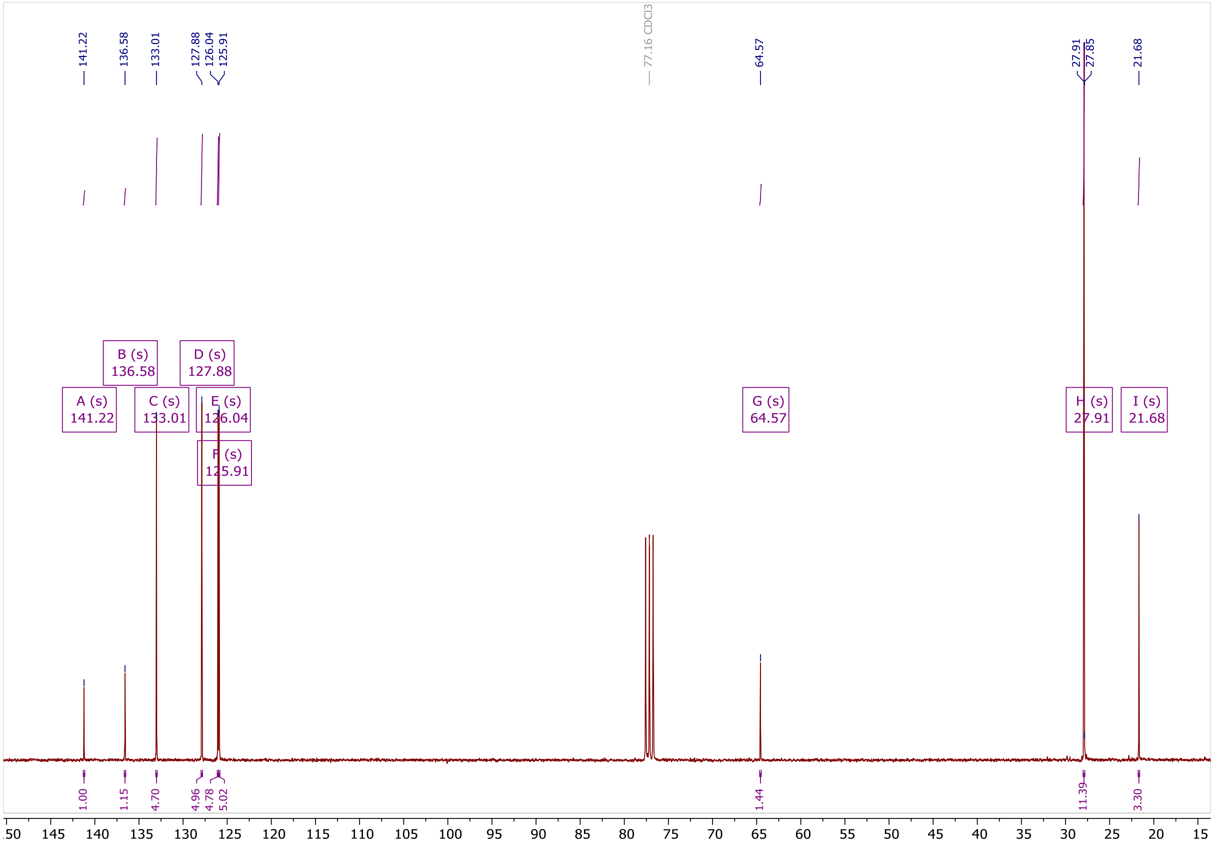

Figure S37: ^{13}C NMR spectrum of substrate **7a** in $CDCl_3$.

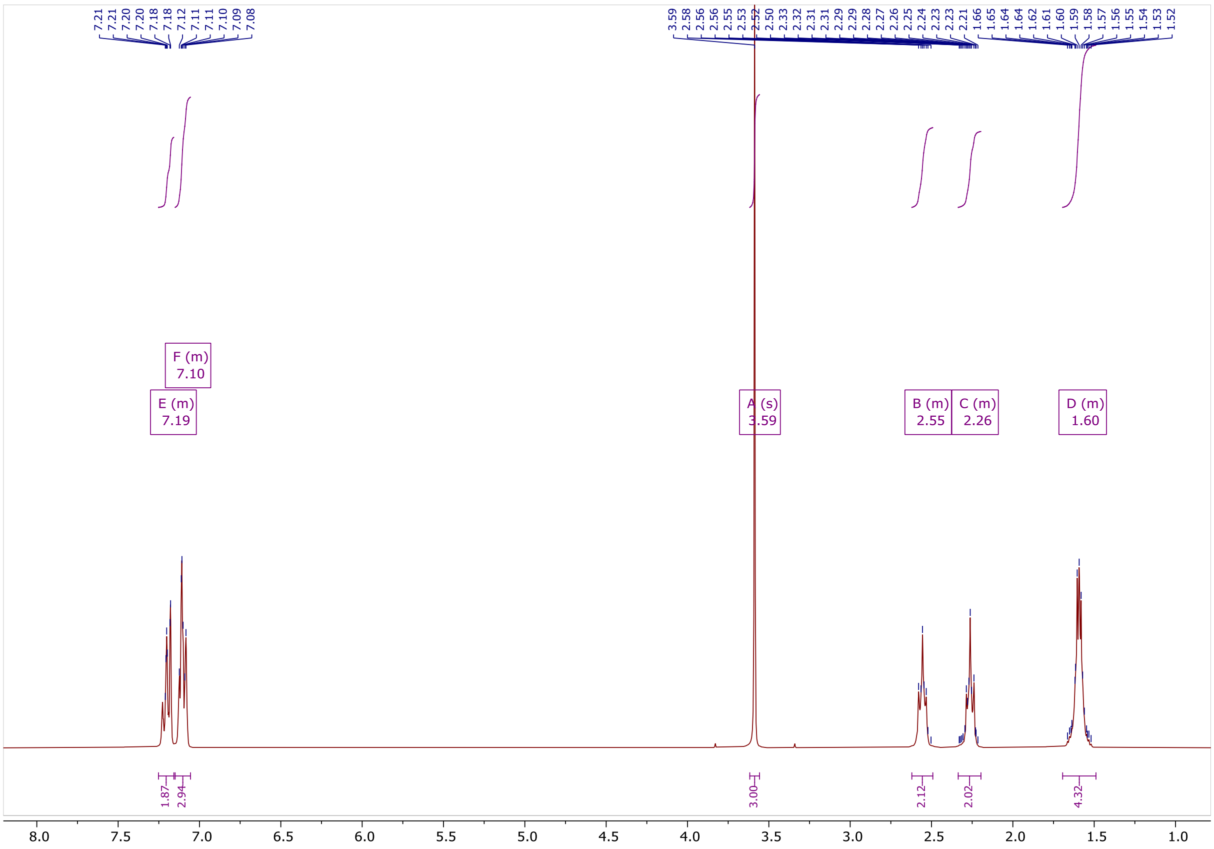

Figure S38: ^{1}H NMR spectrum of methyl 5-phenylpentanoate in $CDCl_3$.

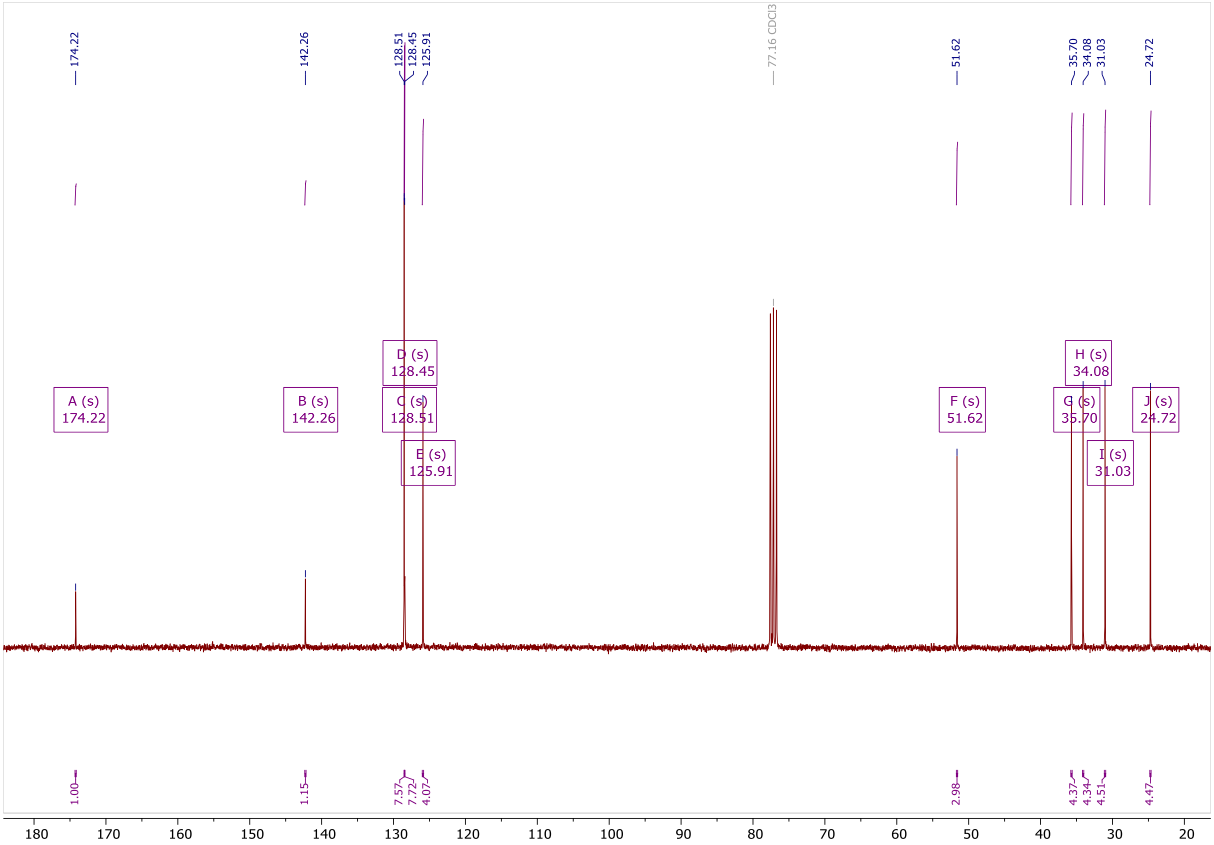

Figure S39: ^{13}C NMR spectrum of methyl 5-phenylpentanoate in $CDCl_3$.

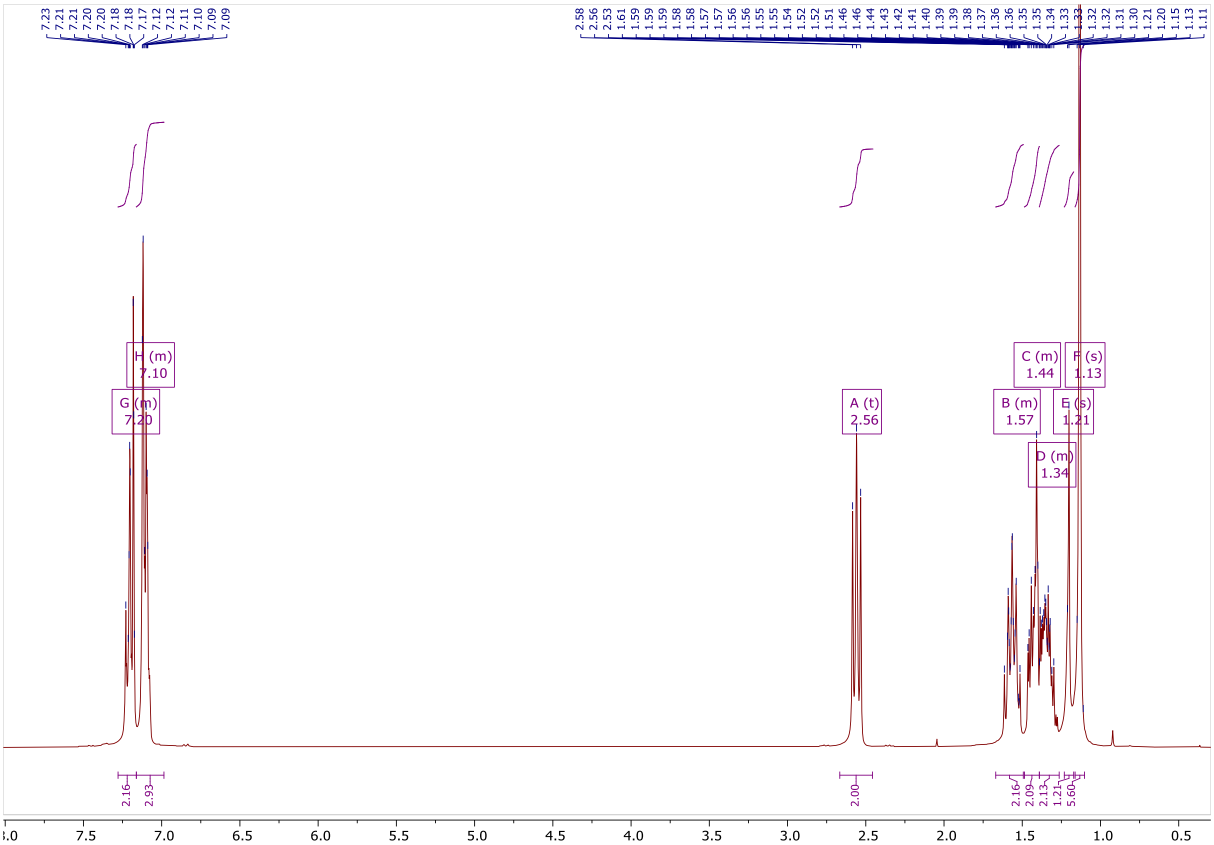

Figure S40: ^{1}H NMR spectrum of 2-methyl-6-phenylhexan-2-ol in $CDCl_3$.

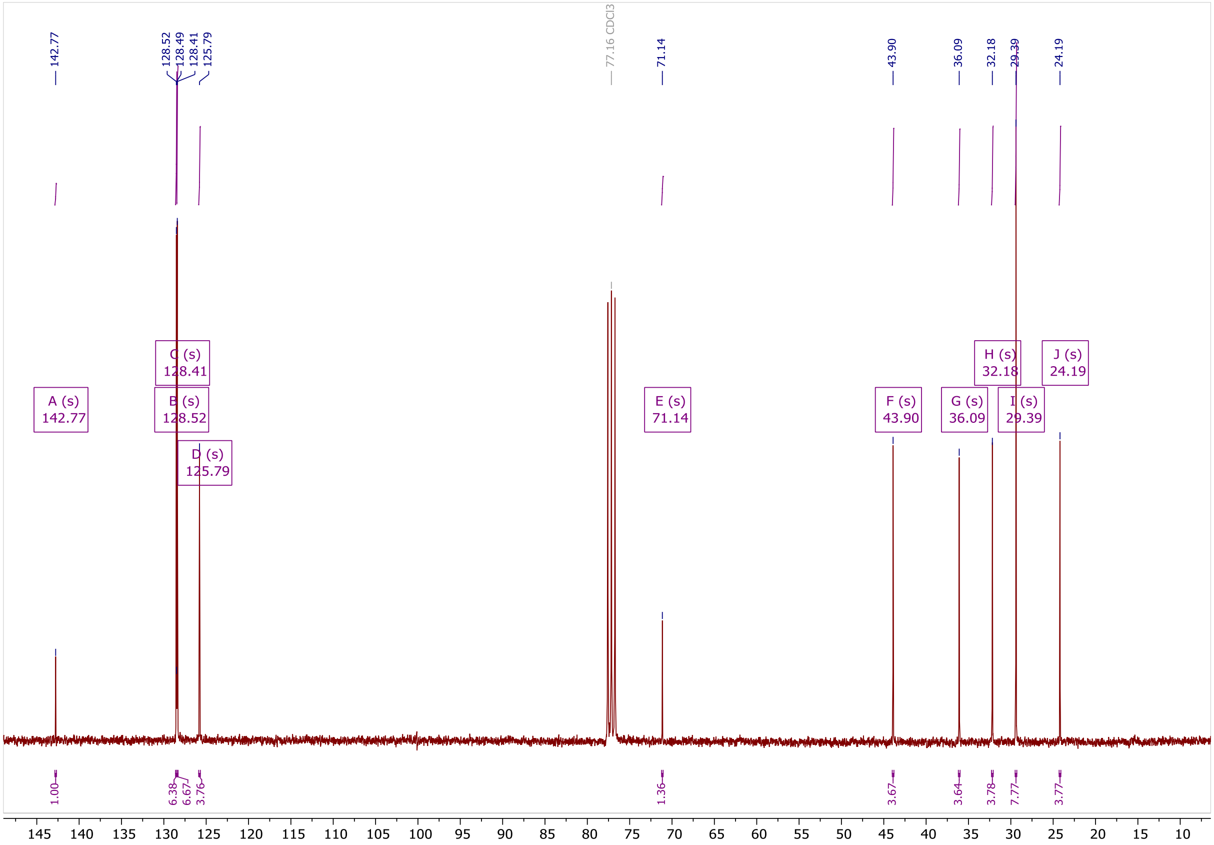

Figure S41: ^{13}C NMR spectrum of 2-methyl-6-phenylhexan-2-ol in $CDCl_3$.

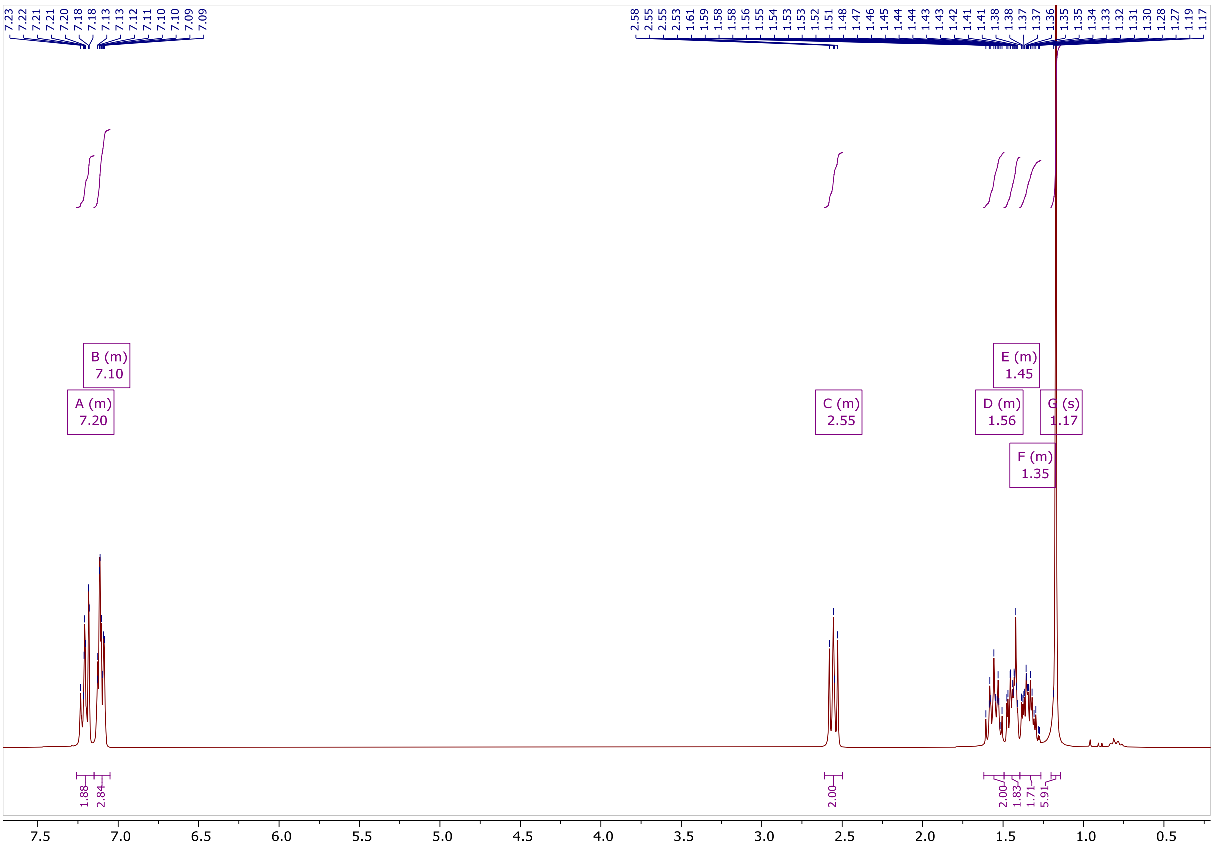

Figure S42: ^{1}H NMR spectrum of substrate **8a** in $CDCl_3$.

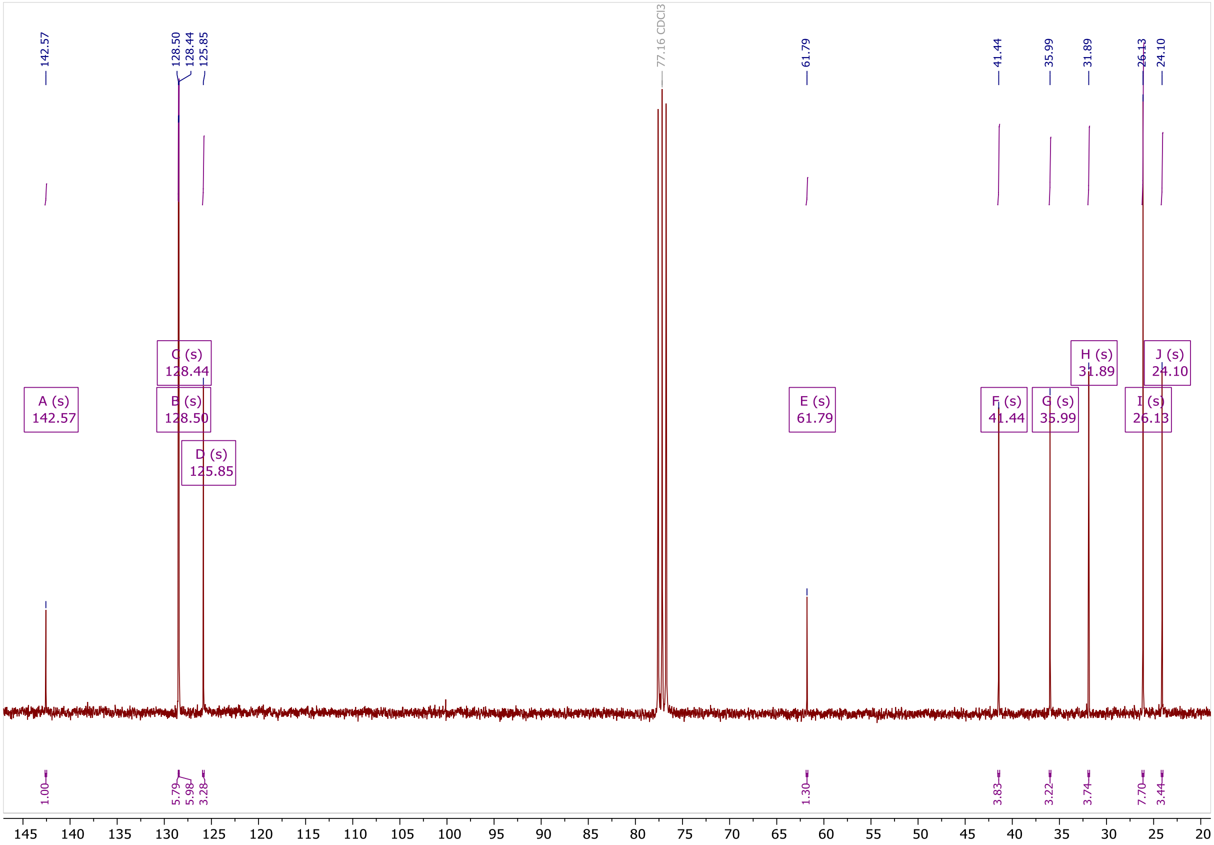

Figure S43: ^{13}C NMR spectrum of **8a** in $CDCl_3$.

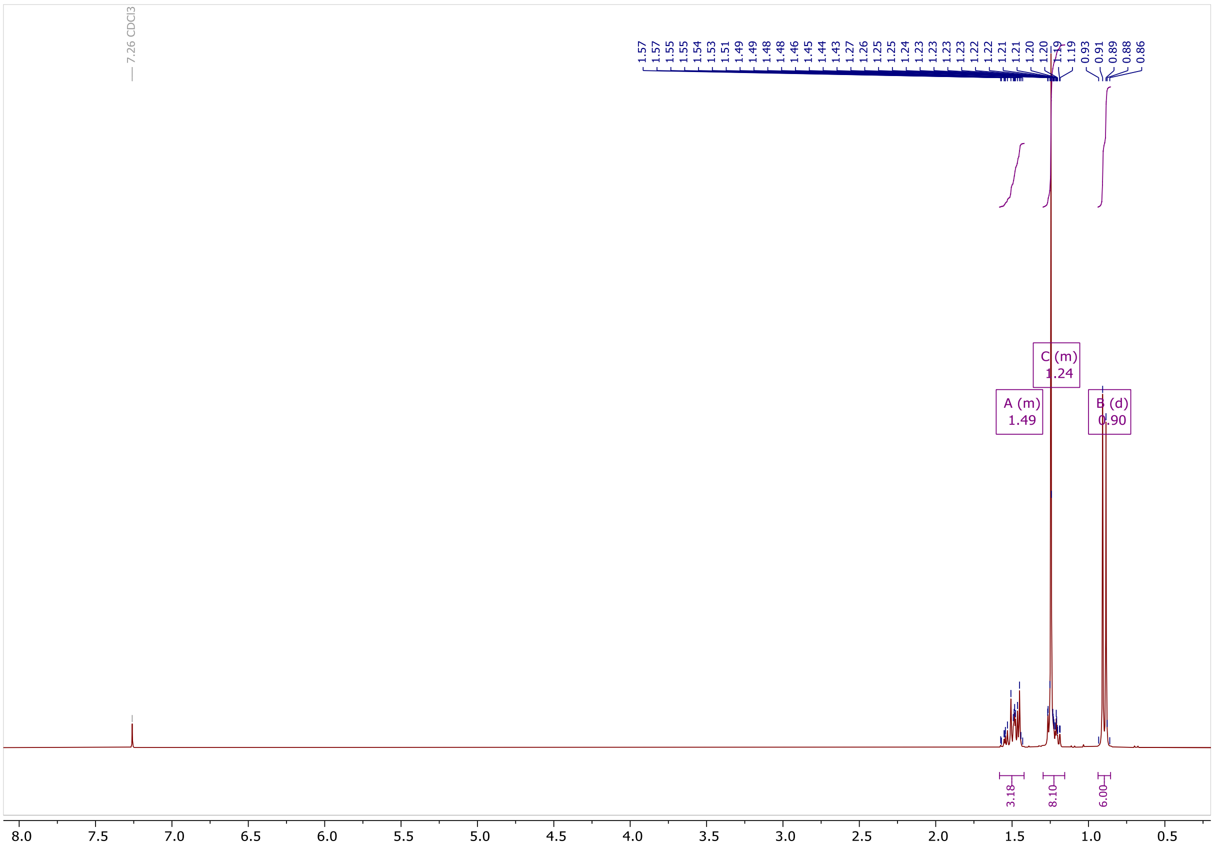

Figure S44: ^{1}H NMR spectrum of substrate **9a** in $CDCl_3$.

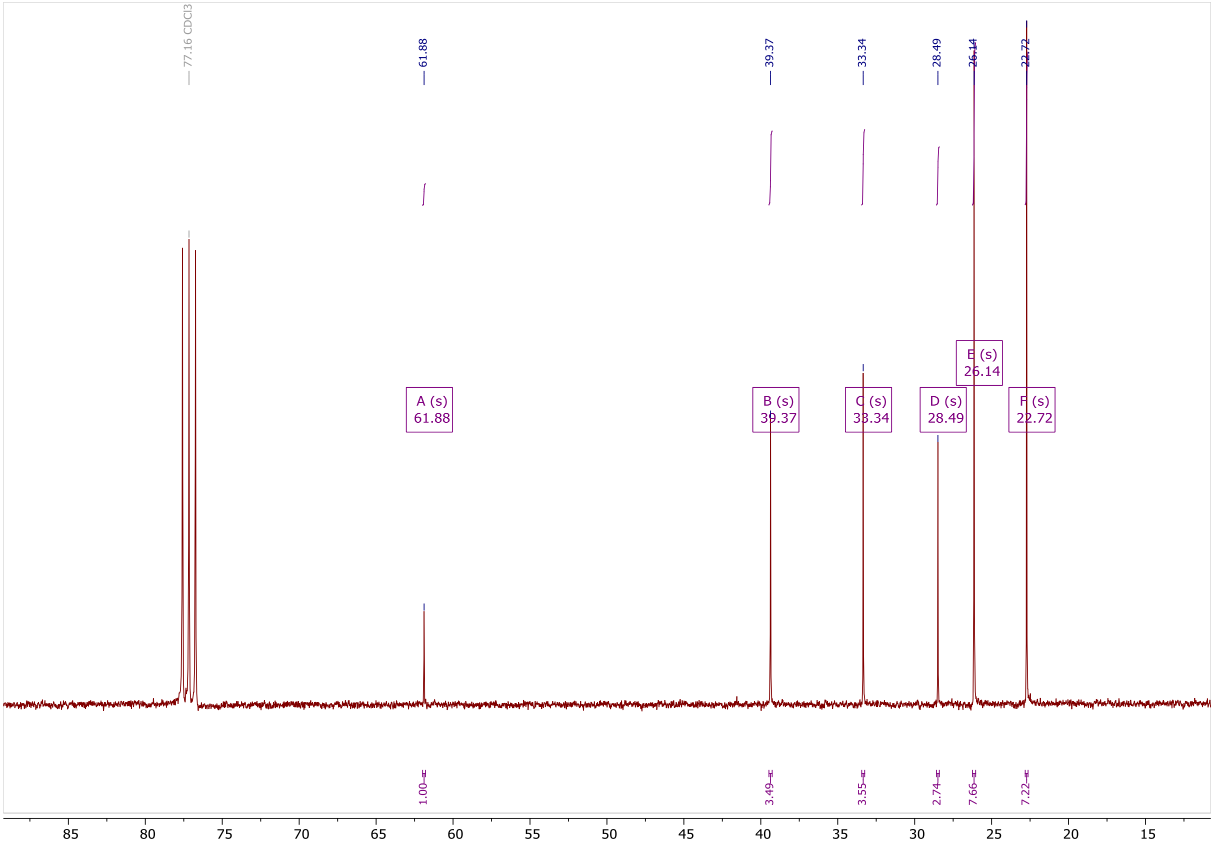

Figure S45: ^{13}C NMR spectrum of substrate **9a** in $CDCl_3$.

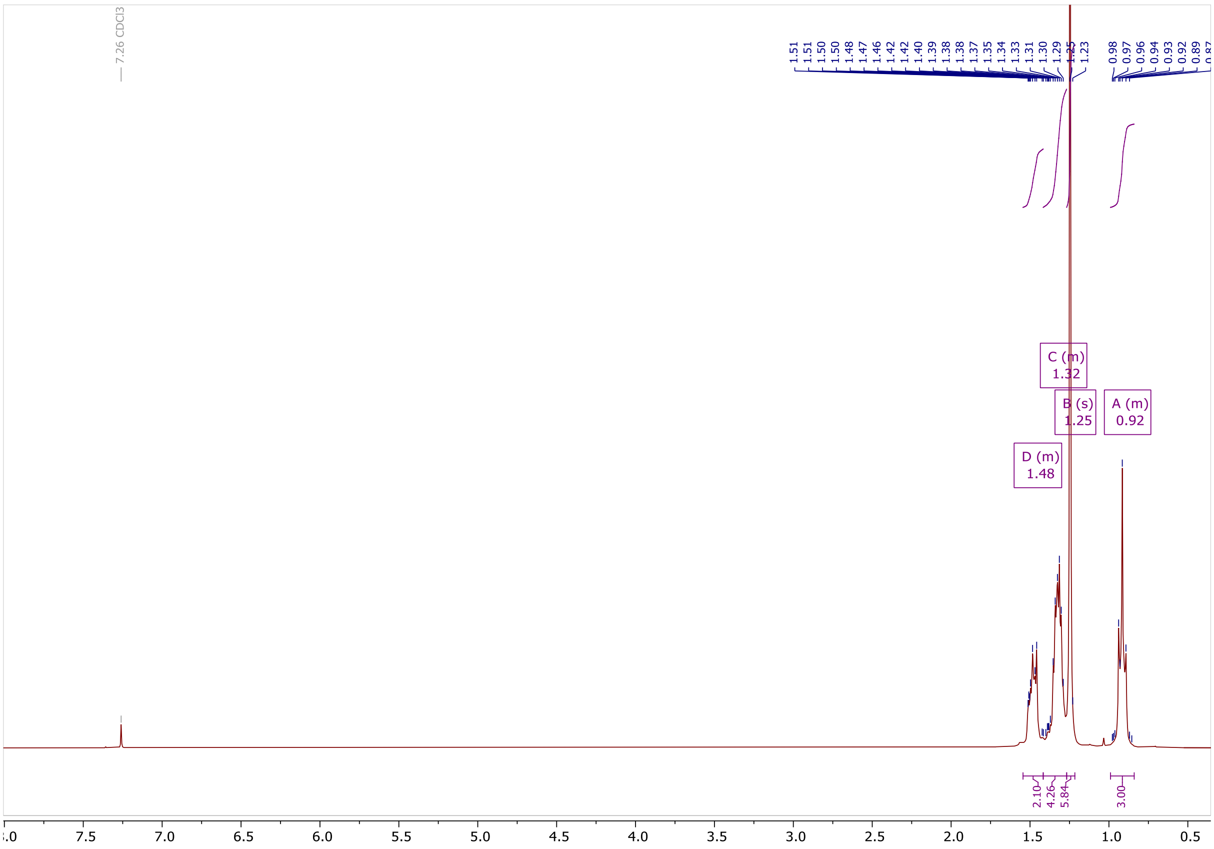

Figure S46: ^{1}H NMR spectrum of substrate **10a** in $CDCl_3$.

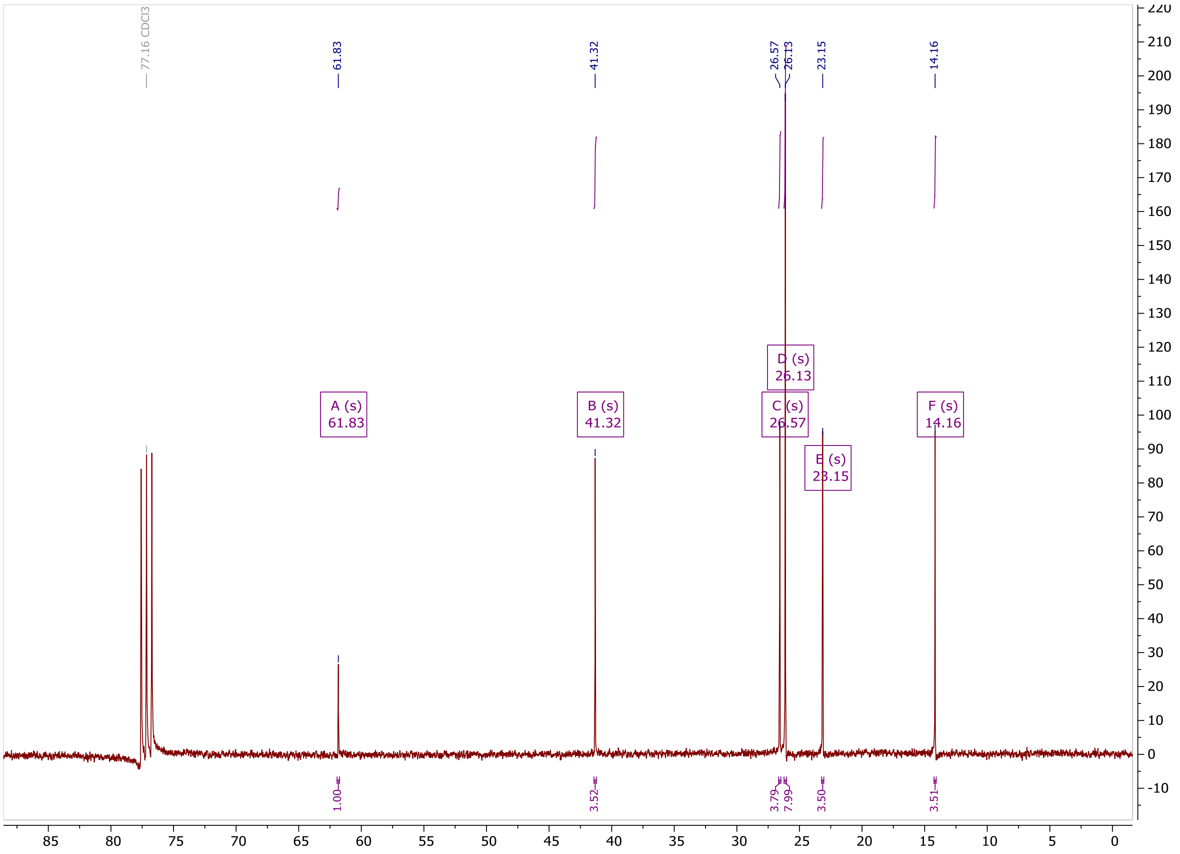

Figure S47: ^{13}C NMR spectrum of substrate **10a** in $CDCl_3$.

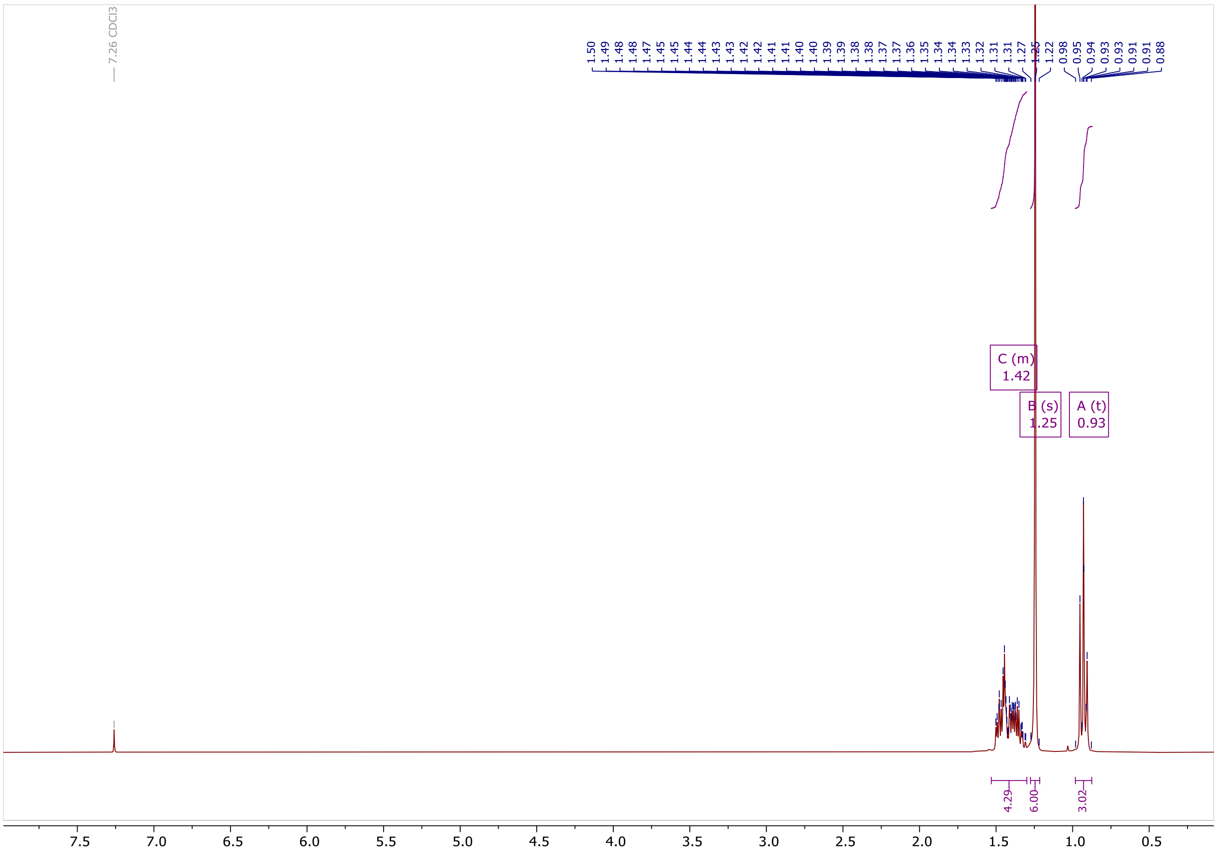

Figure S48: ^{1}H NMR spectrum of substrate **11a** in $CDCl_3$.

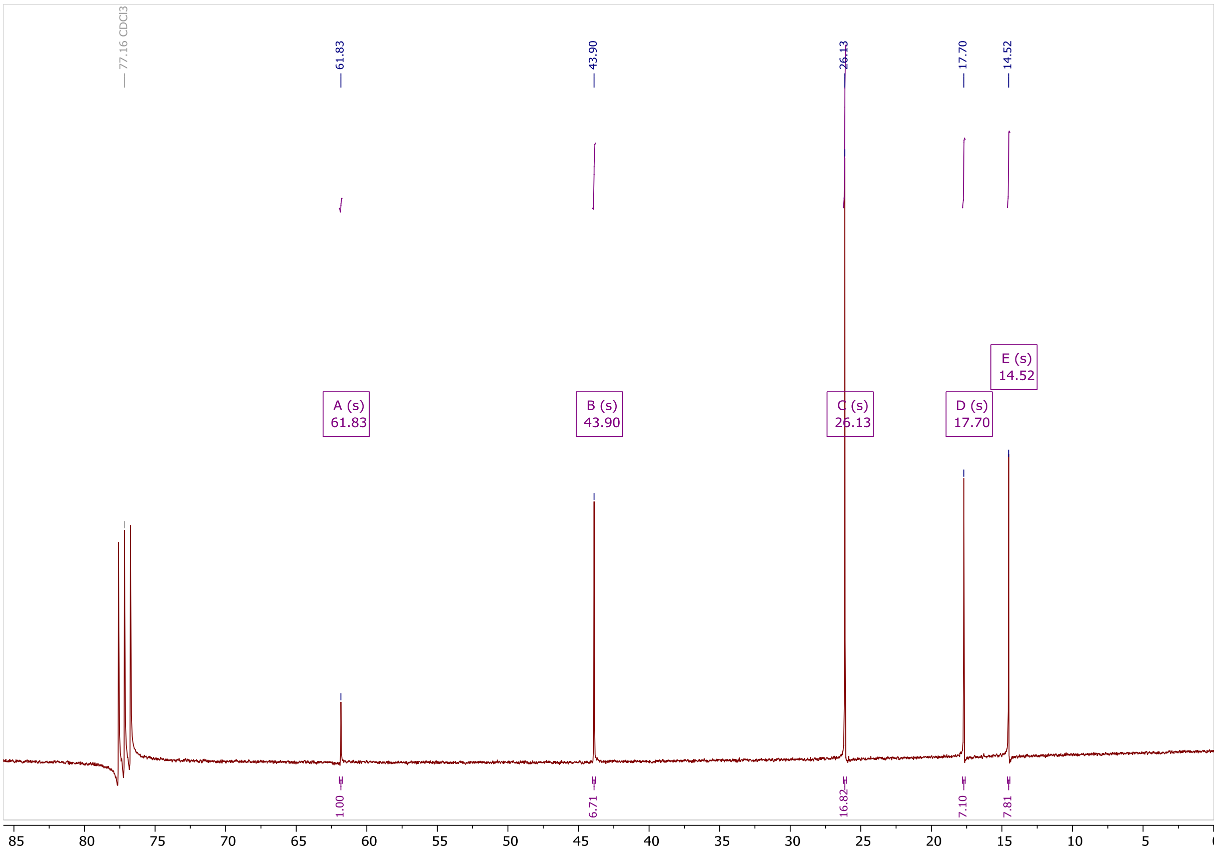

Figure S49: ^{13}C NMR spectrum of substrate **11a** in $CDCl_3$.

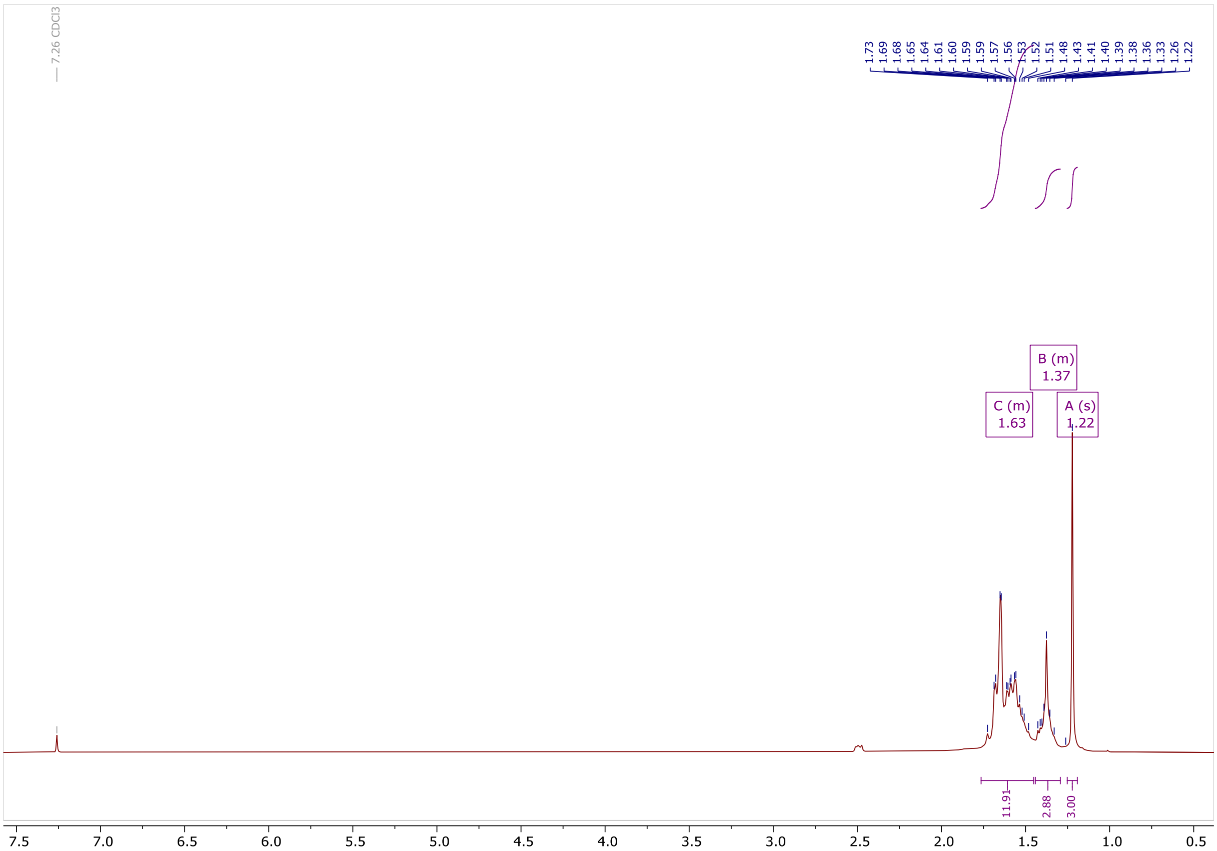

Figure S50: ^{1}H NMR spectrum of 1-methylcycloheptan-1-ol in $CDCl_3$.

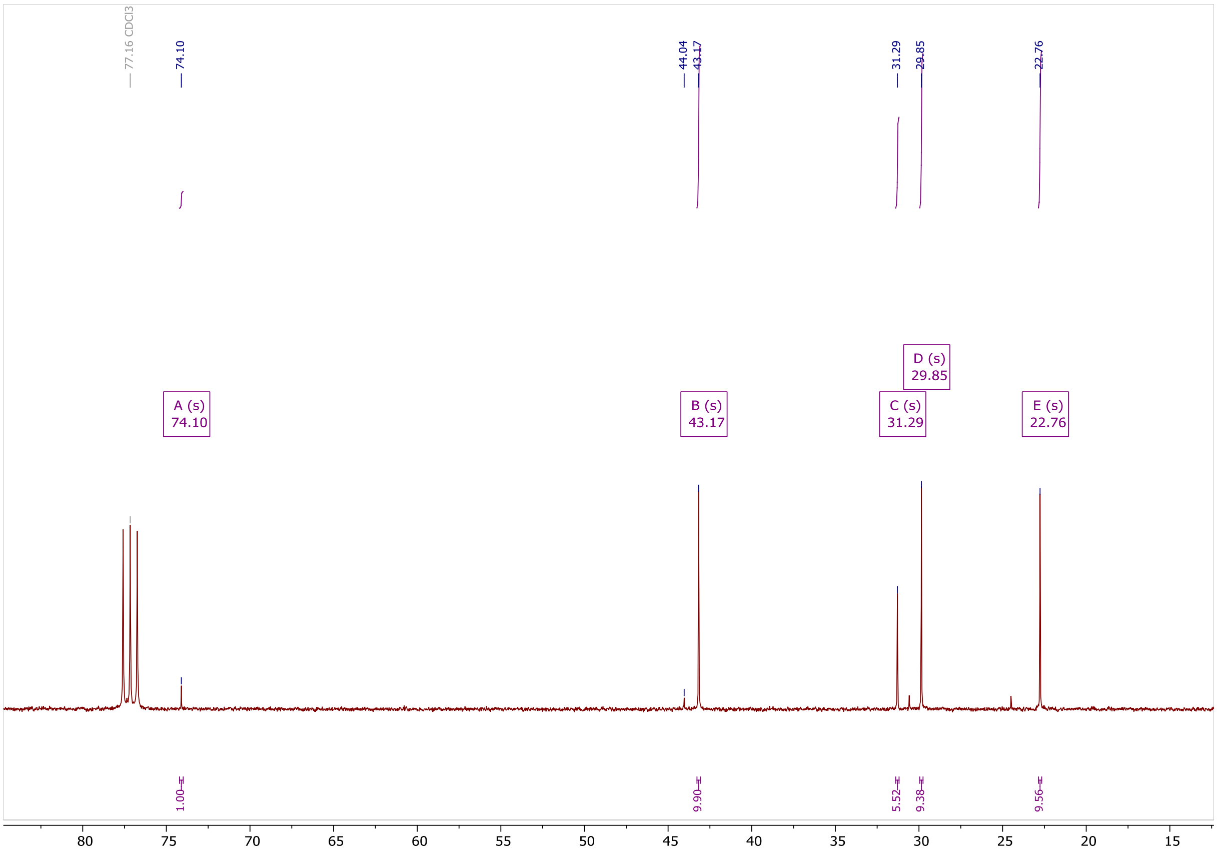

Figure S51: ^{13}C NMR spectrum of 1-methylcycloheptan-1-ol in $CDCl_3$.

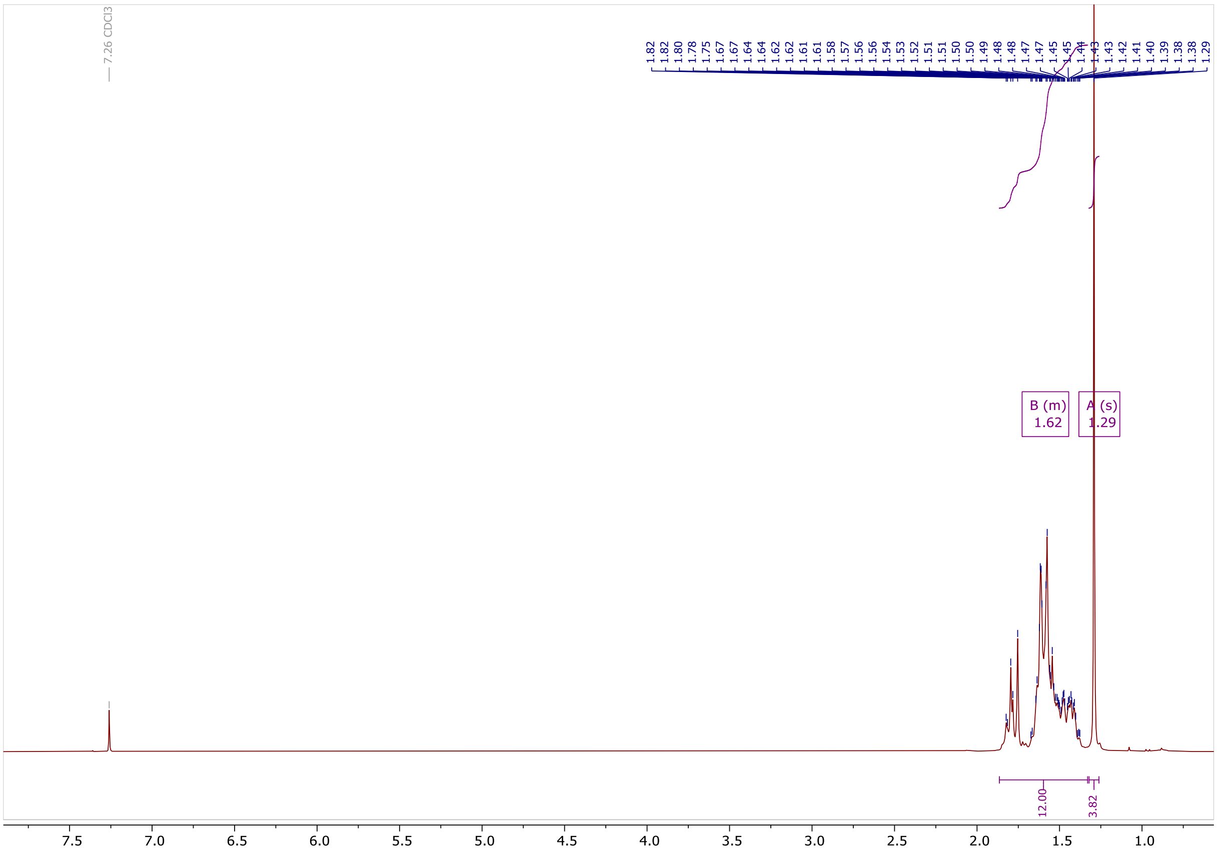

Figure S52: ^{1}H NMR spectrum of substrate **12a** in $CDCl_3$.

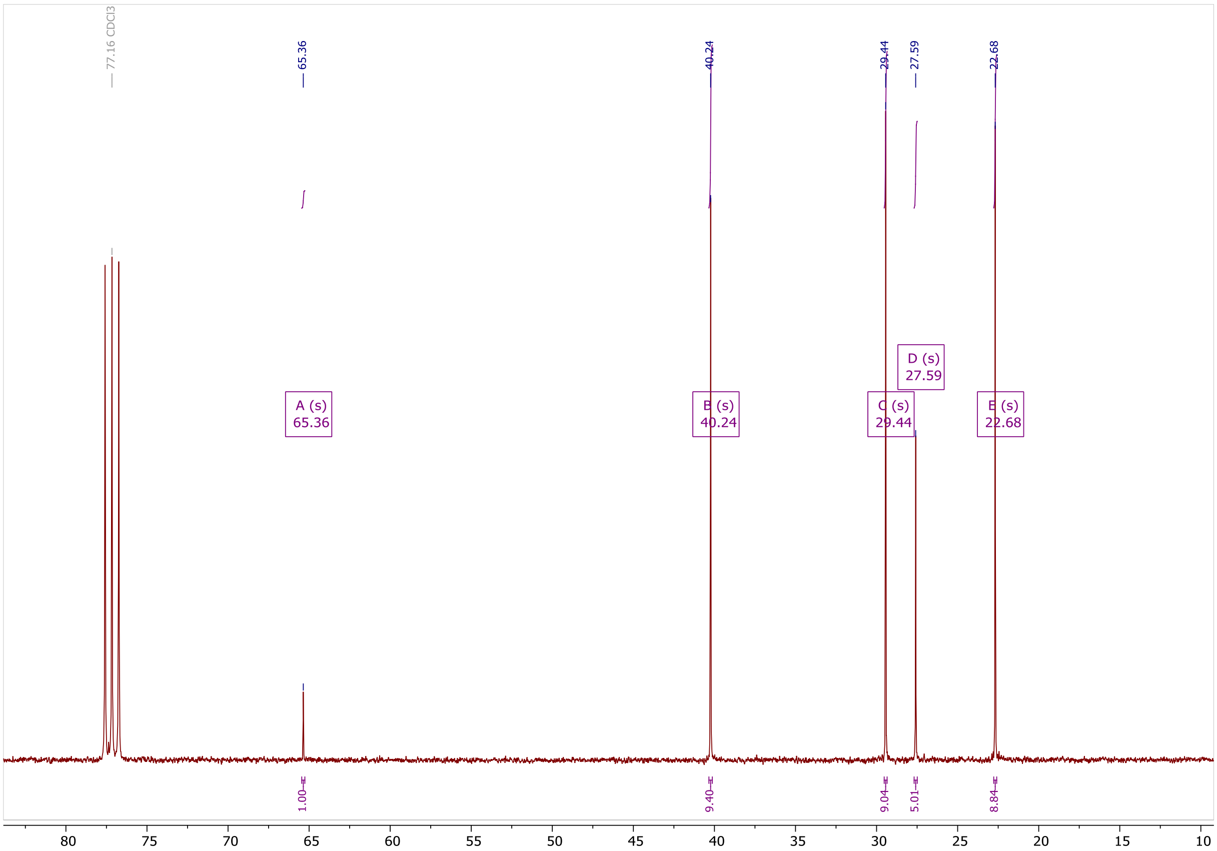

Figure S53: ^{13}C NMR spectrum of substrate **12a** in $CDCl_3$.

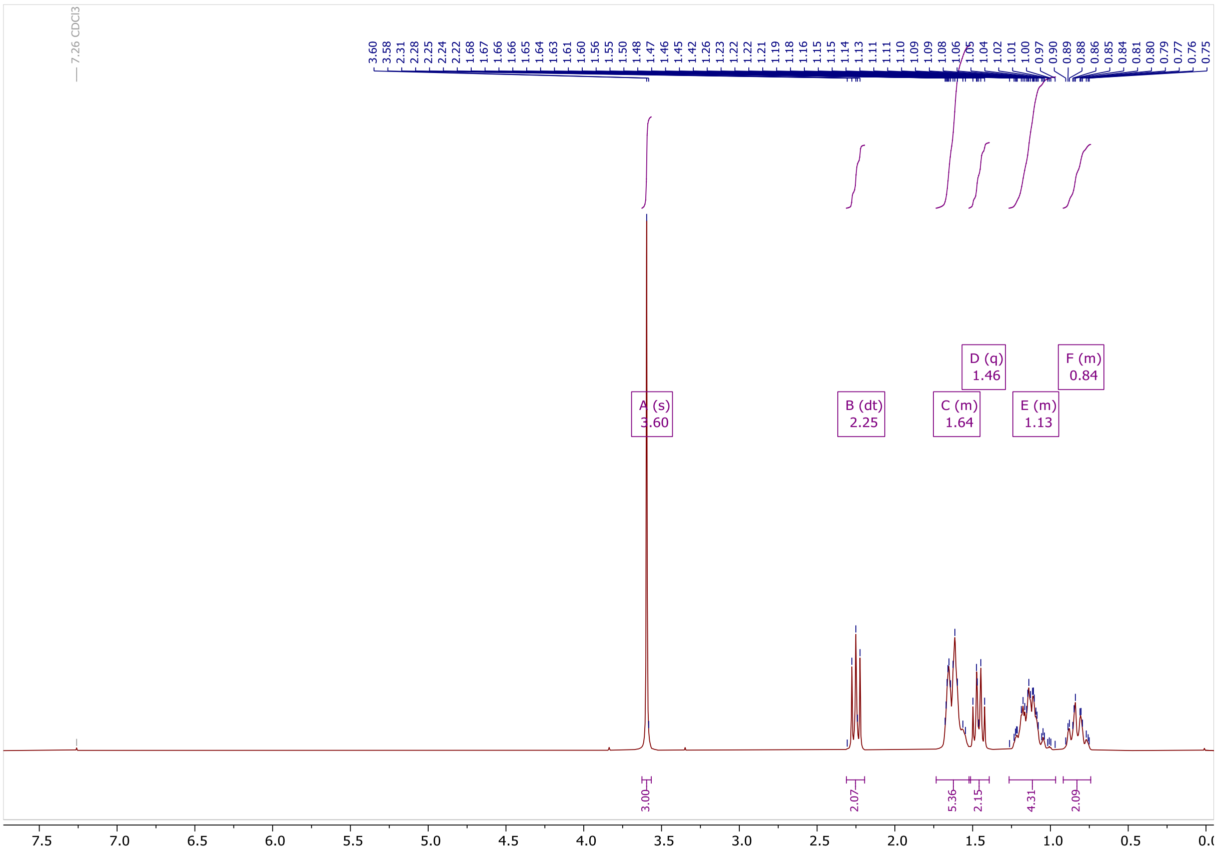

Figure S54: ^{1}H NMR spectrum of methyl 3-cyclohexylpropanoate in $CDCl_3$.

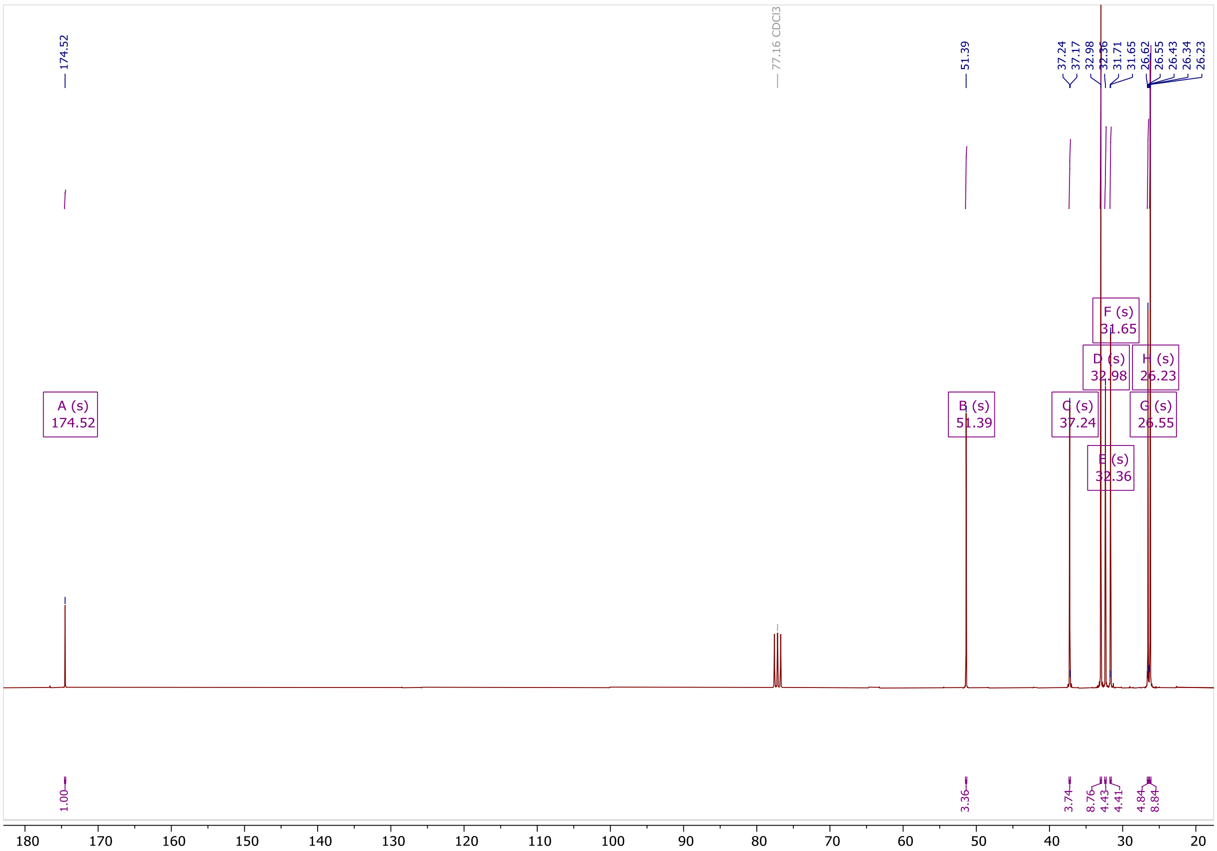

Figure S55: ^{13}C NMR spectrum of methyl 3-cyclohexylpropanoate in $CDCl_3$.

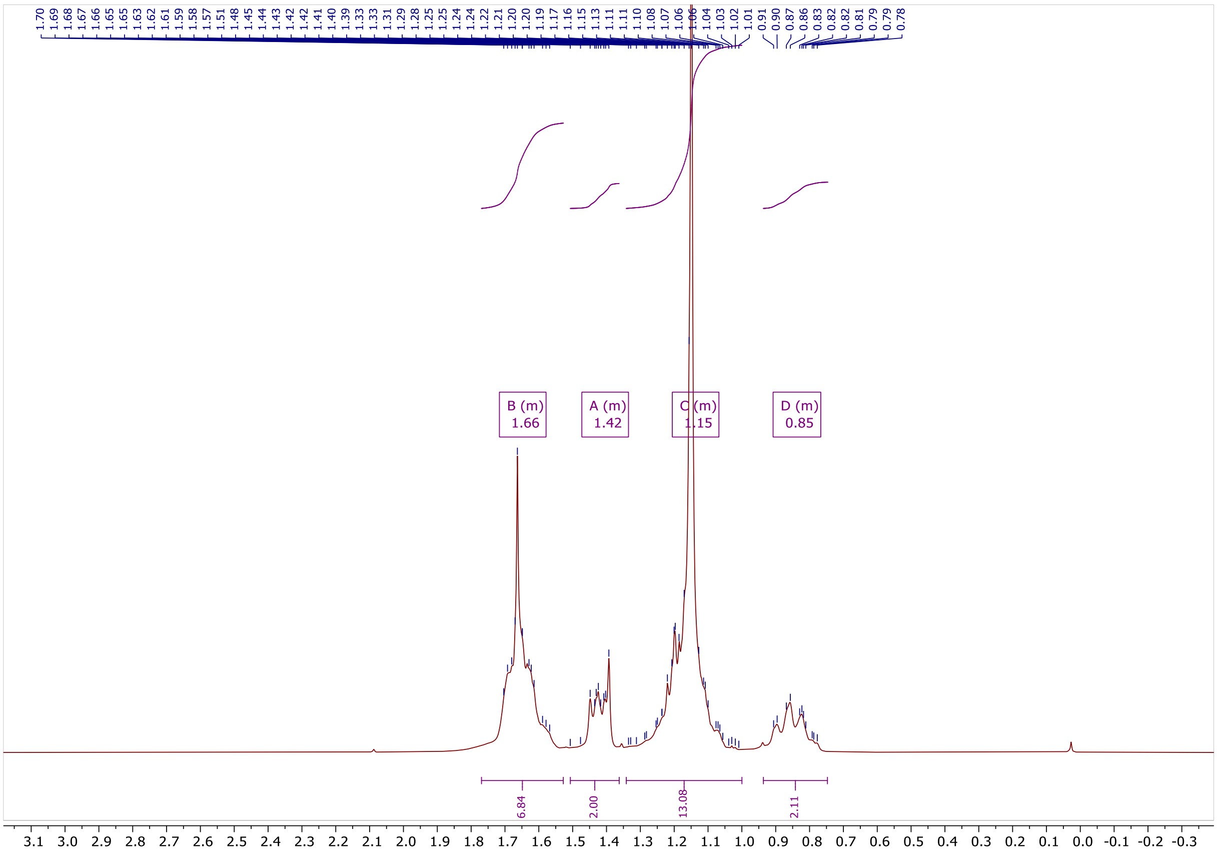

Figure S56: ^{1}H NMR spectrum of 4-cyclohexyl-2-methylbutan-2-ol in $CDCl_3$.

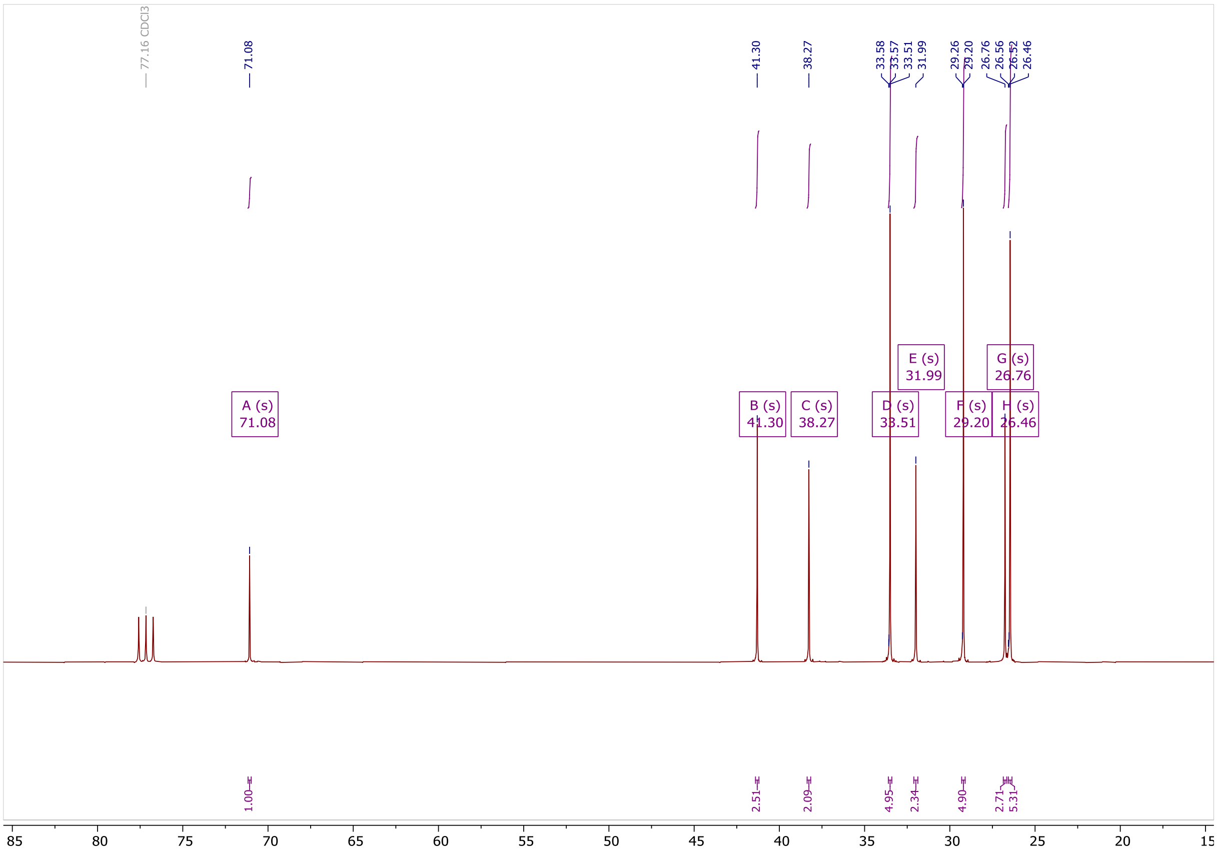

Figure S57: ^{13}C NMR spectrum of 4-cyclohexyl-2-methylbutan-2-ol in $CDCl_3$.

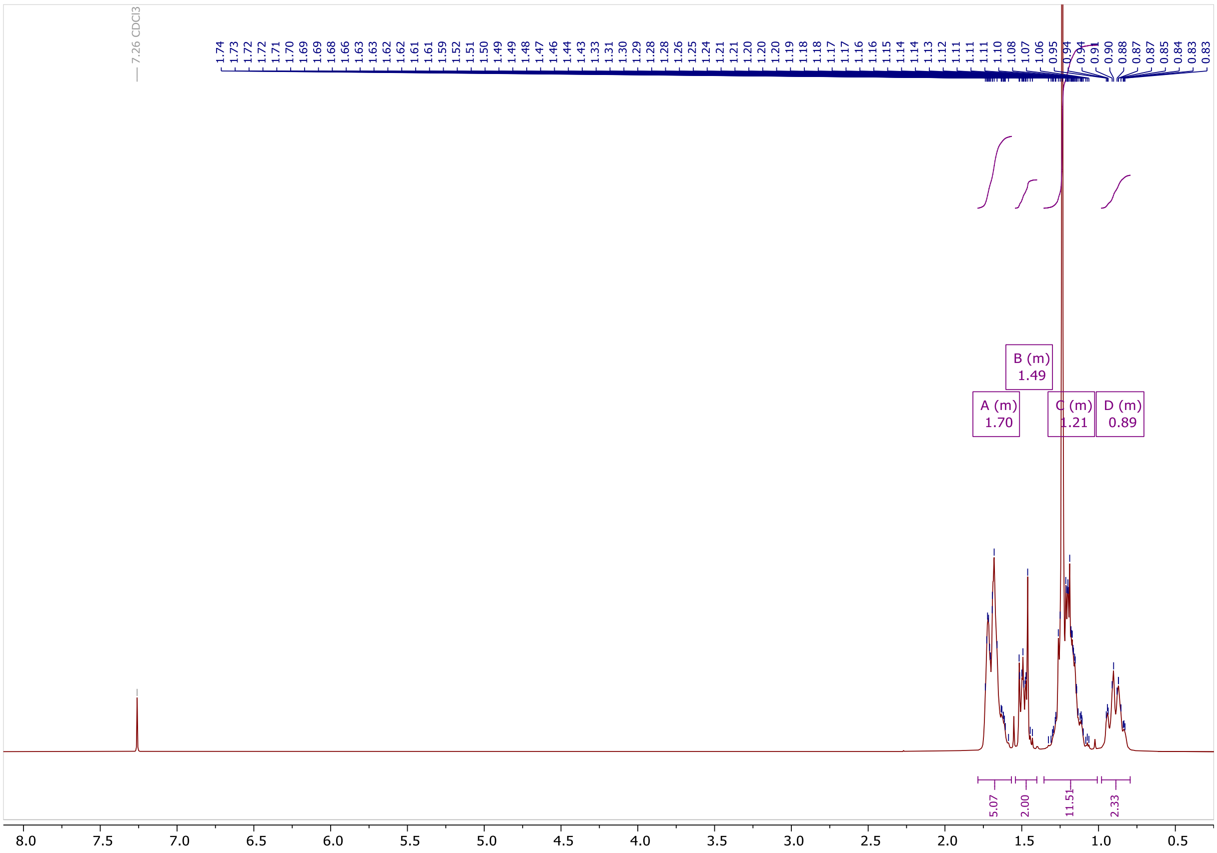

Figure S58: ^{1}H NMR spectrum of substrate **13a** in $CDCl_3$.

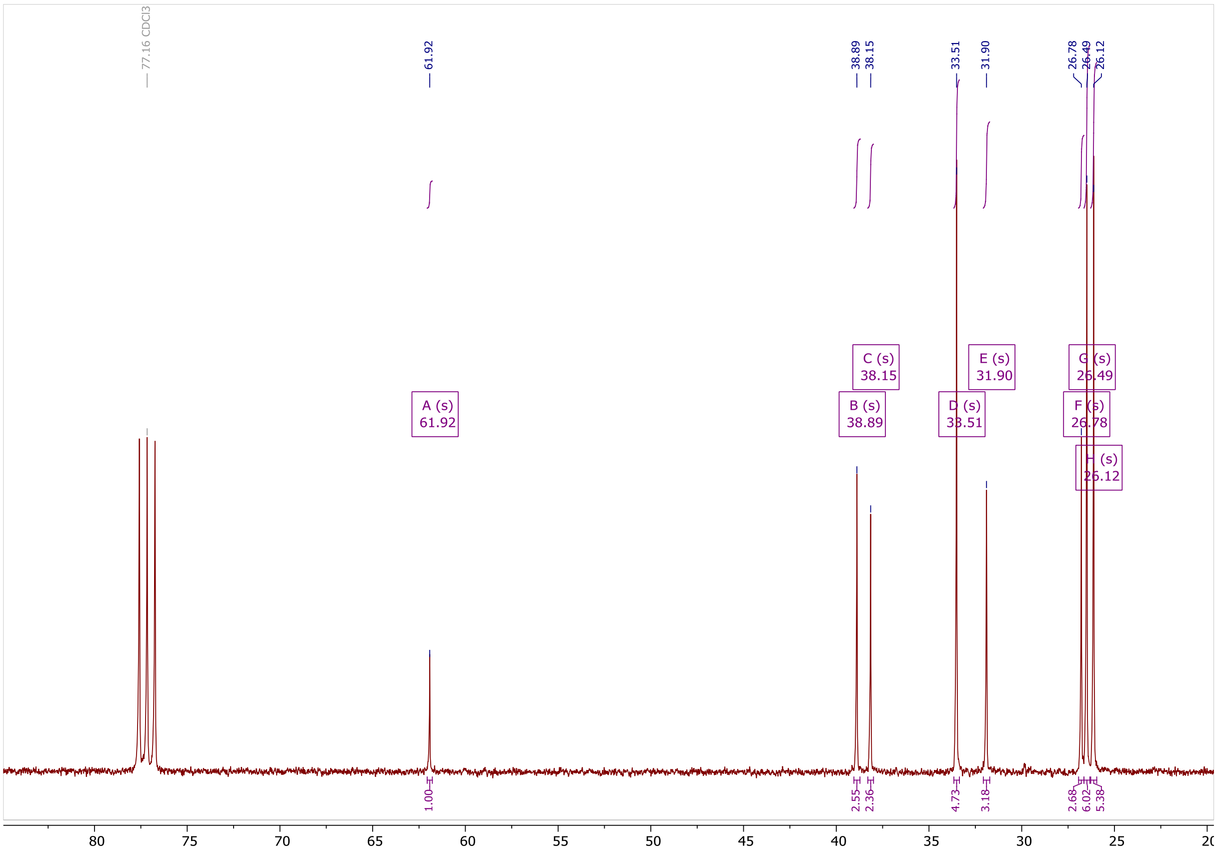

Figure S59: ^{13}C NMR spectrum of substrate **13a** in $CDCl_3$.

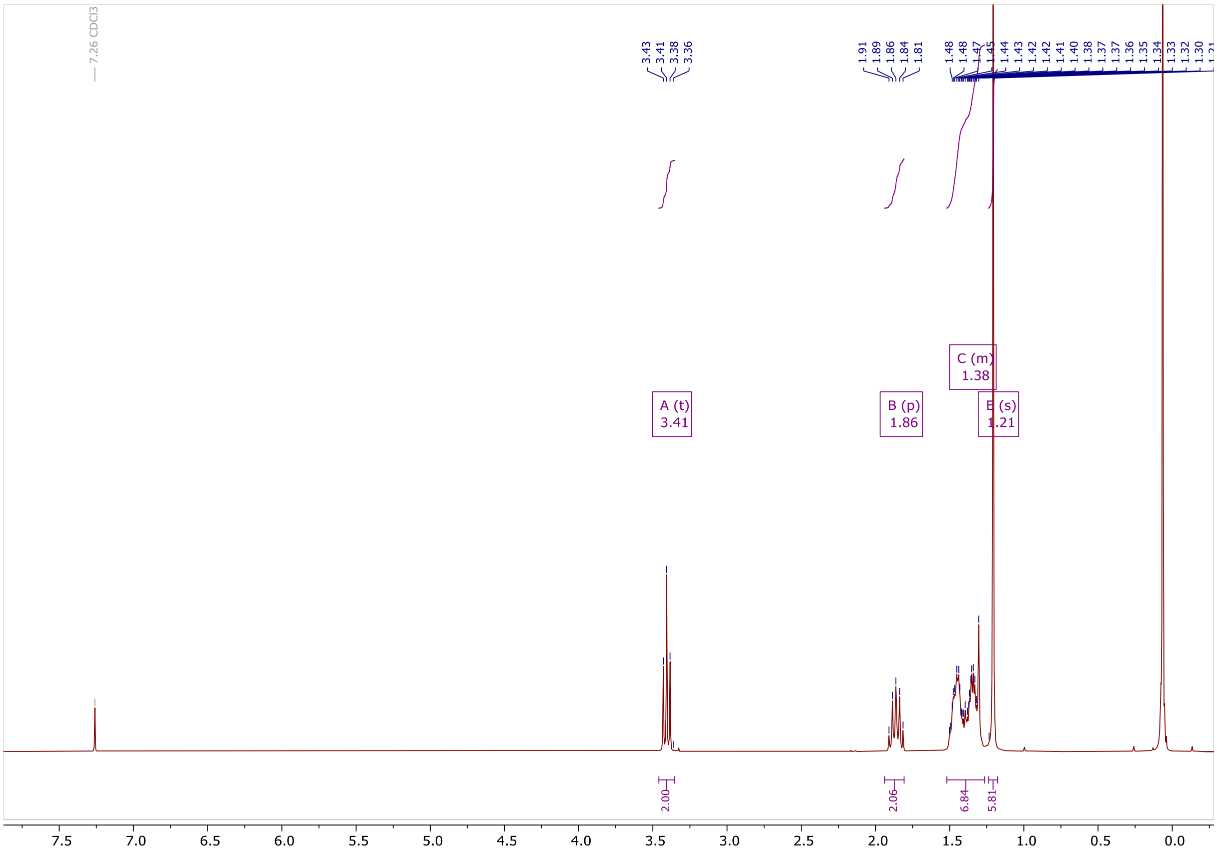

Figure S60: ^{1}H NMR spectrum of 8-bromo-2-methyloctan-2-ol in $CDCl_3$.

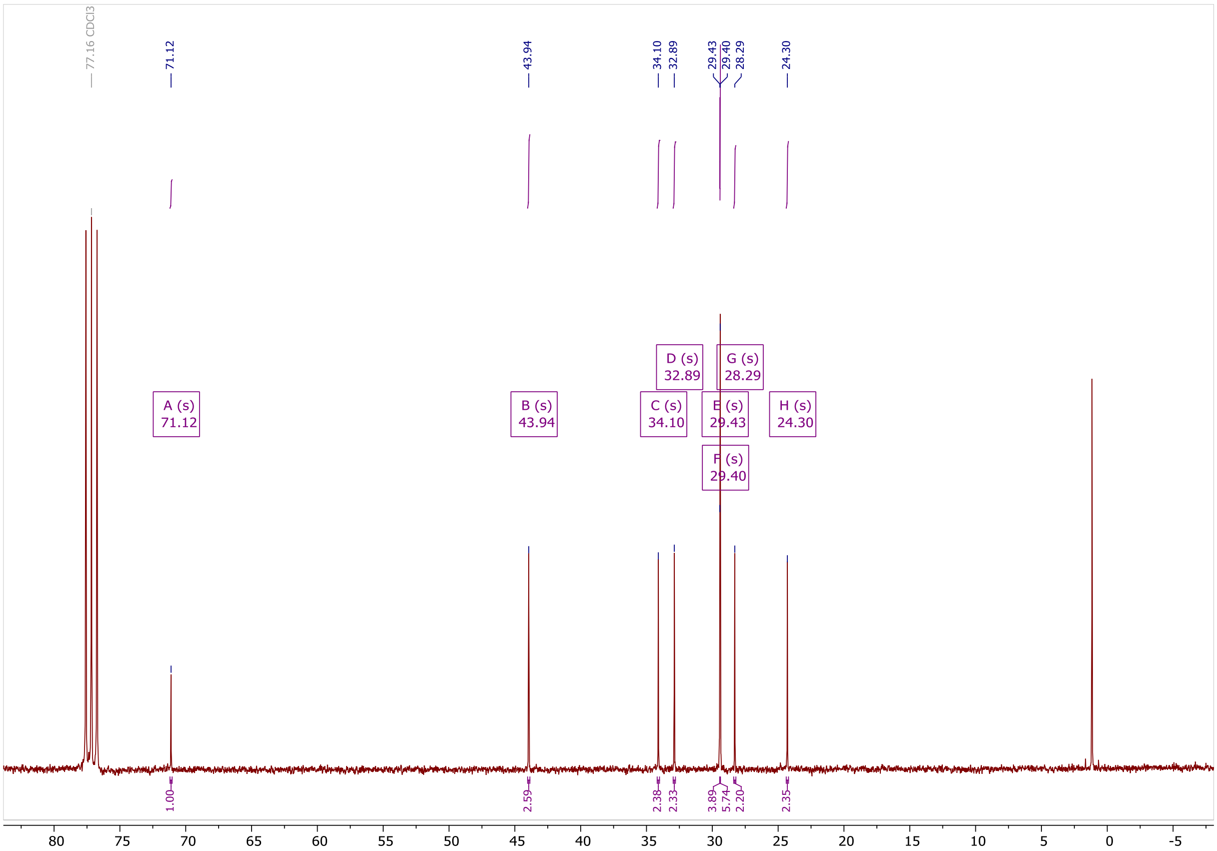

Figure S61: ^{13}C NMR spectrum of 8-bromo-2-methyloctan-2-ol in $CDCl_3$.

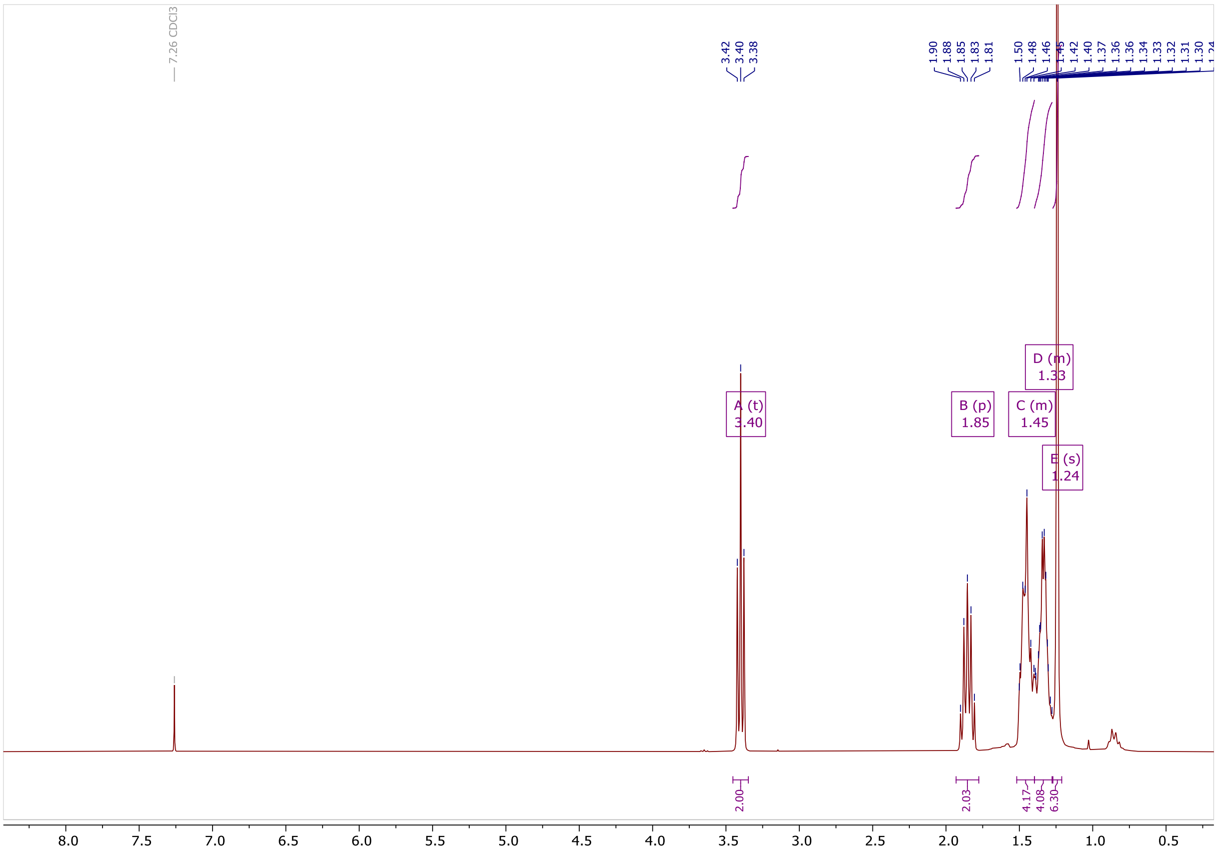

Figure S62: ^{13}C NMR spectrum of substrate **13a** in $CDCl_3$.

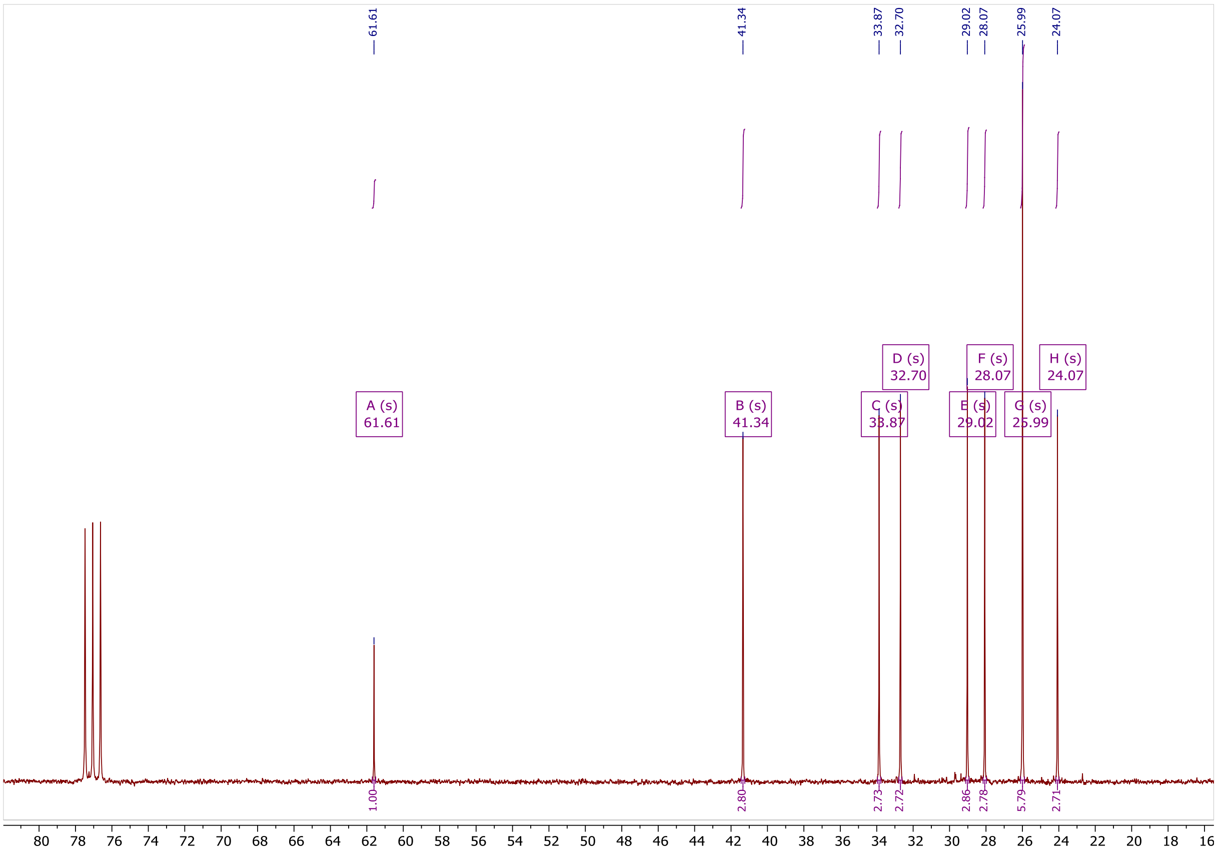

Figure S63: ^{13}C NMR spectrum of substrate **13a** in $CDCl_3$.

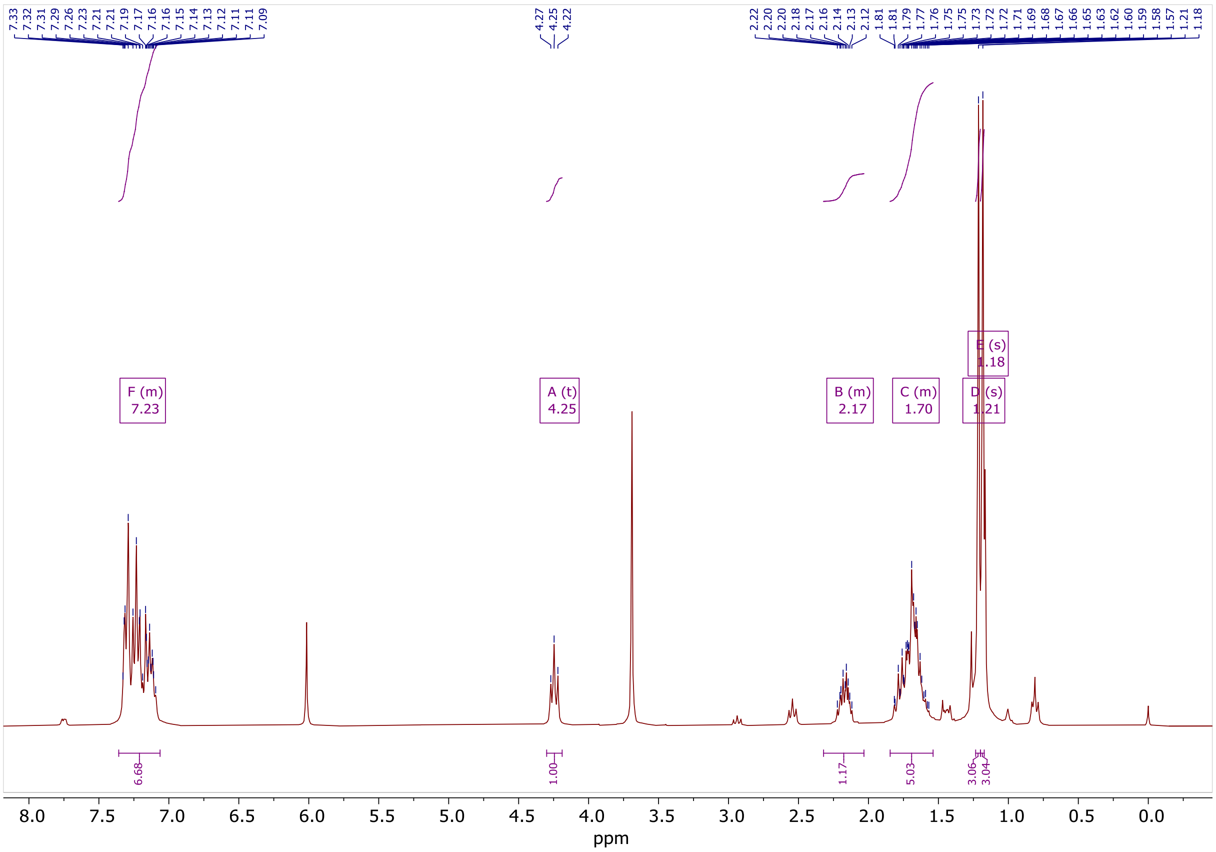

Figure S64: ^{1}H NMR spectrum of crude **1b** (t = 30 min) in $CDCl_3$ with 1,3,5-Trimethoxybenzene as internal standard.

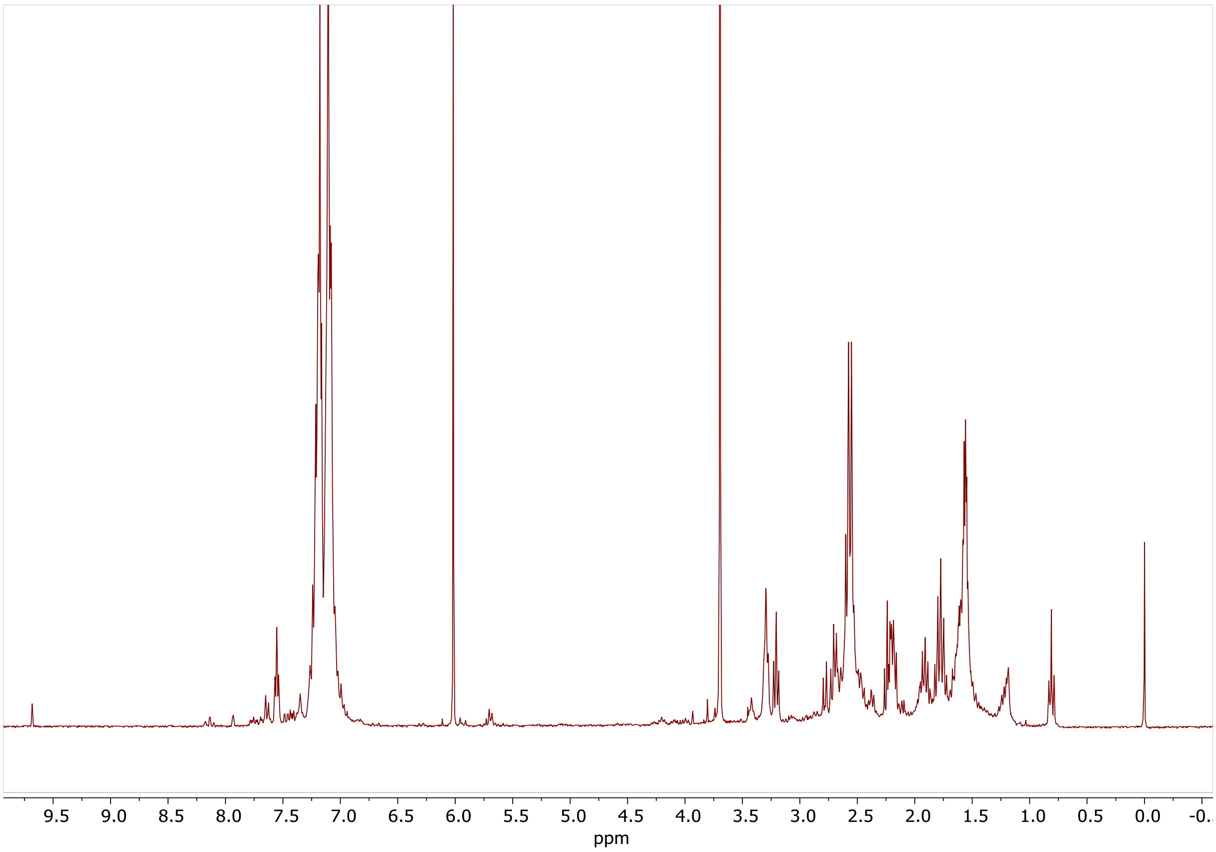

Figure S65: ^{1}H NMR spectrum of crude reaction mixture with **2a** (t = 30 min) in $CDCl_3$ with 1,3,5-Trimethoxybenzene as internal standard.

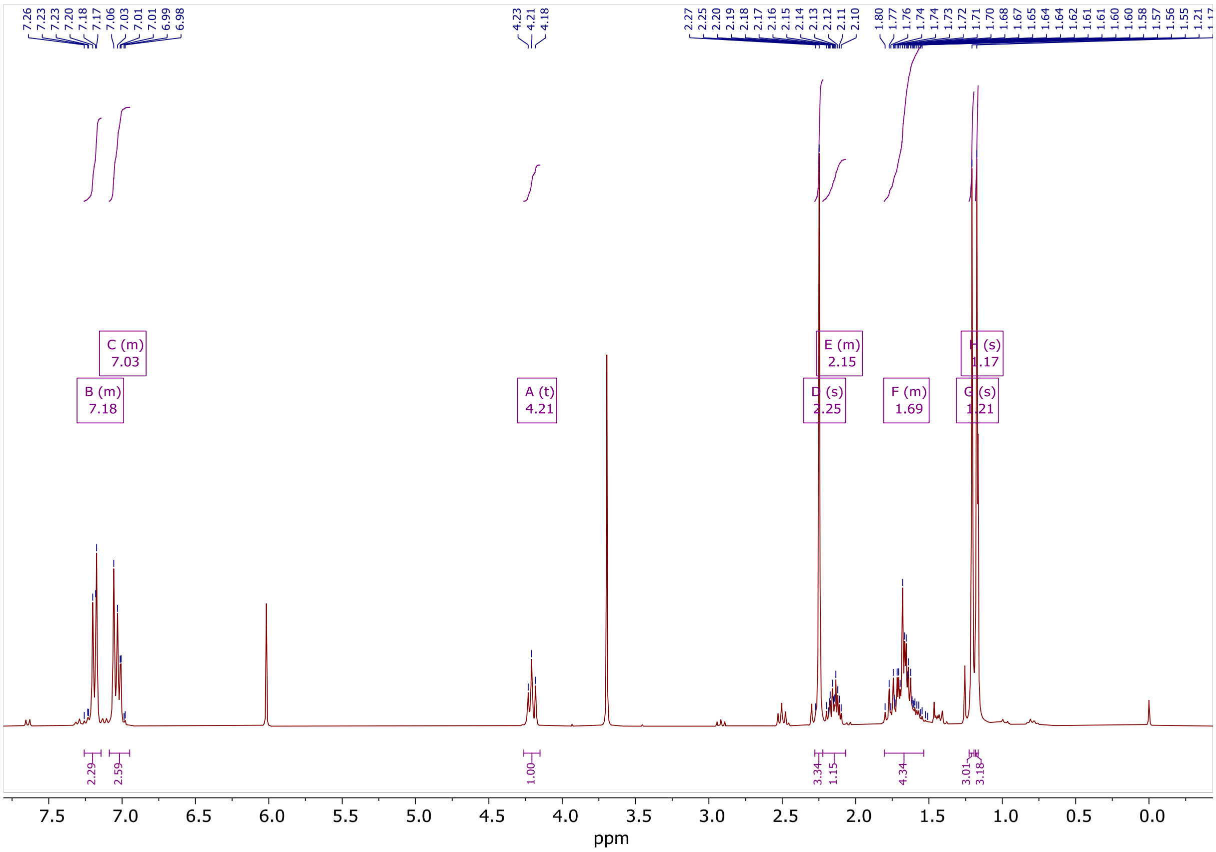

Figure 66: ^{1}H NMR spectrum of crude **3b** (t = 30 min) in $CDCl_3$ with 1,3,5-Trimethoxybenzene as internal standard.

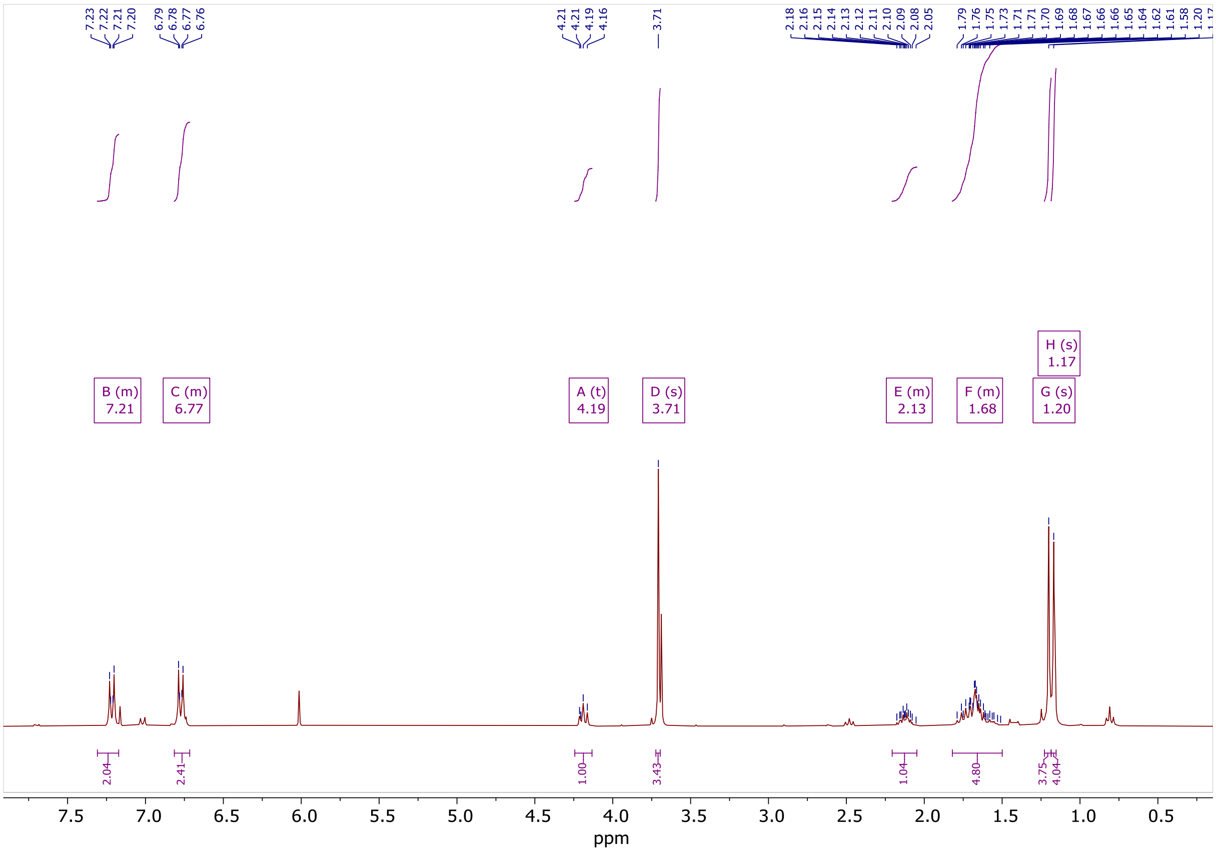

Figure S67: ^{1}H NMR spectrum of crude **4b** (t = 30 min) in $CDCl_3$ with 1,3,5-Trimethoxybenzene as internal standard.

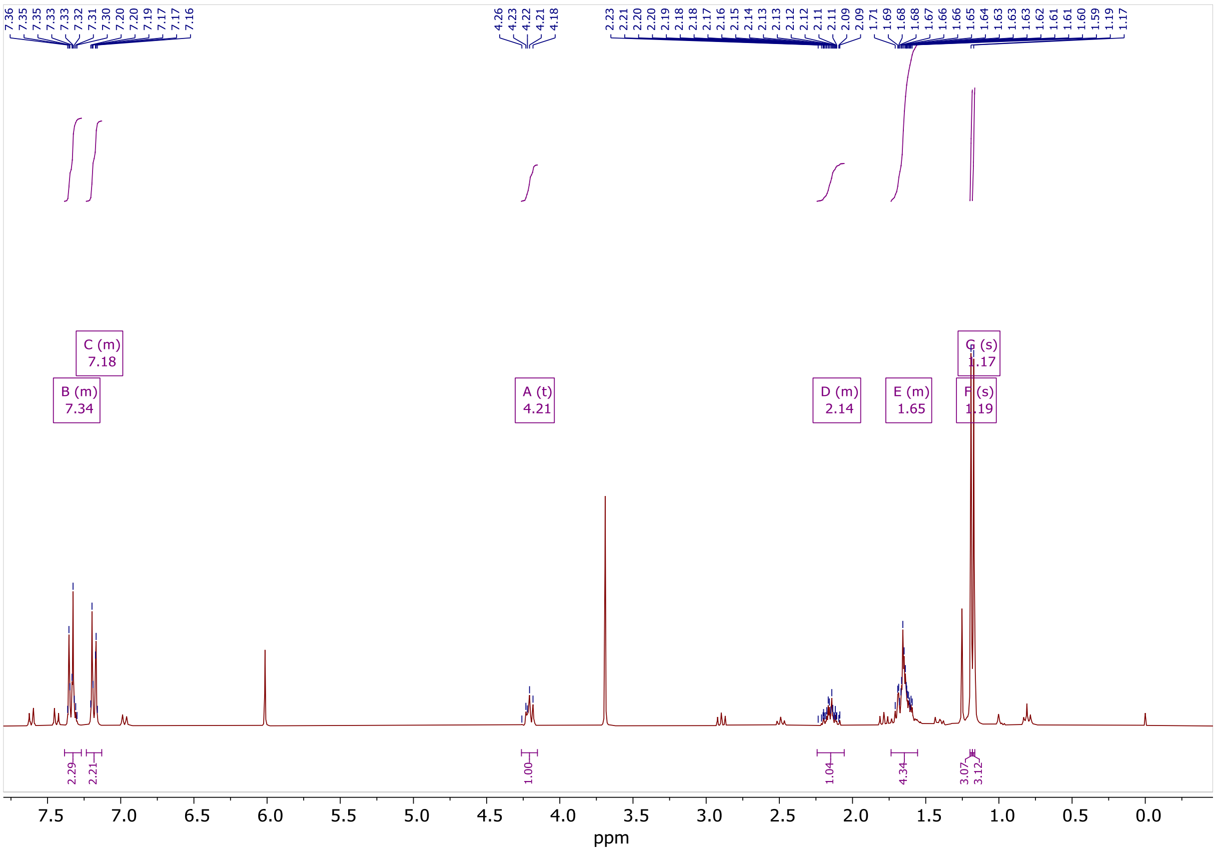

Figure S68: ^{1}H NMR spectrum of crude **5b** (t = 30 min) in $CDCl_3$ with 1,3,5-Trimethoxybenzene as internal standard.

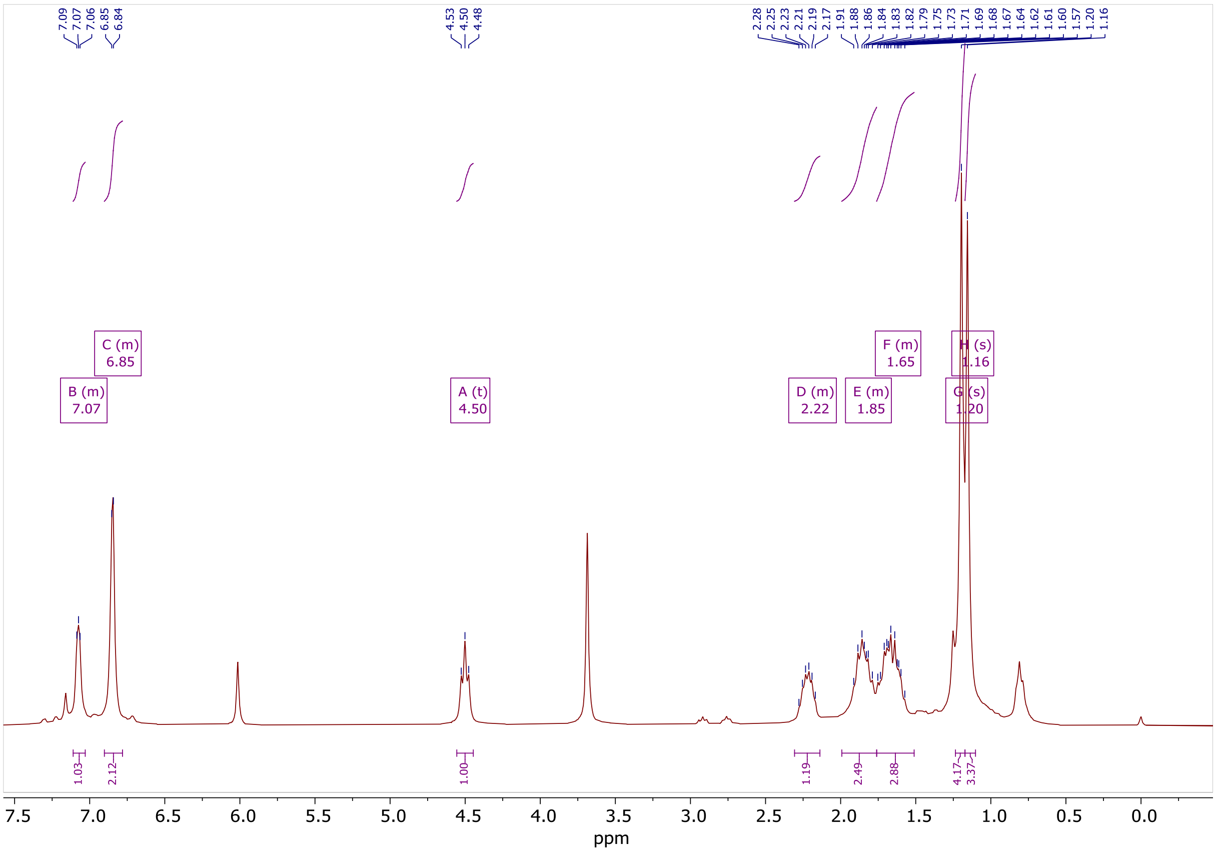

Figure S69: ^1H NMR spectrum of crude **6b** (t = 30 min) in $CDCl_3$ with 1,3,5-Trimethoxybenzene as internal standard.

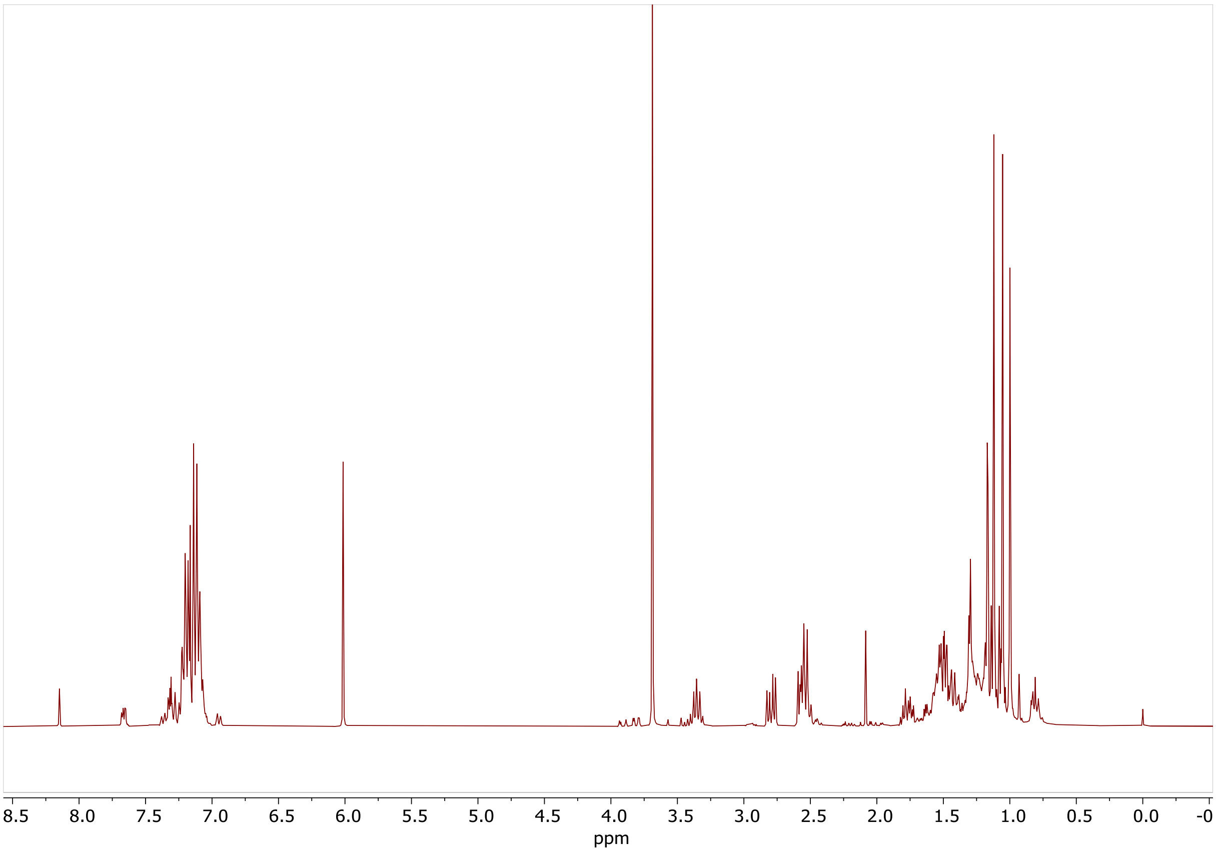

Figure S70: ^1H NMR spectrum of crude reaction mixture with **7a** (t = 6 h) in $CDCl_3$ with 1,3,5-Trimethoxybenzene as internal standard.

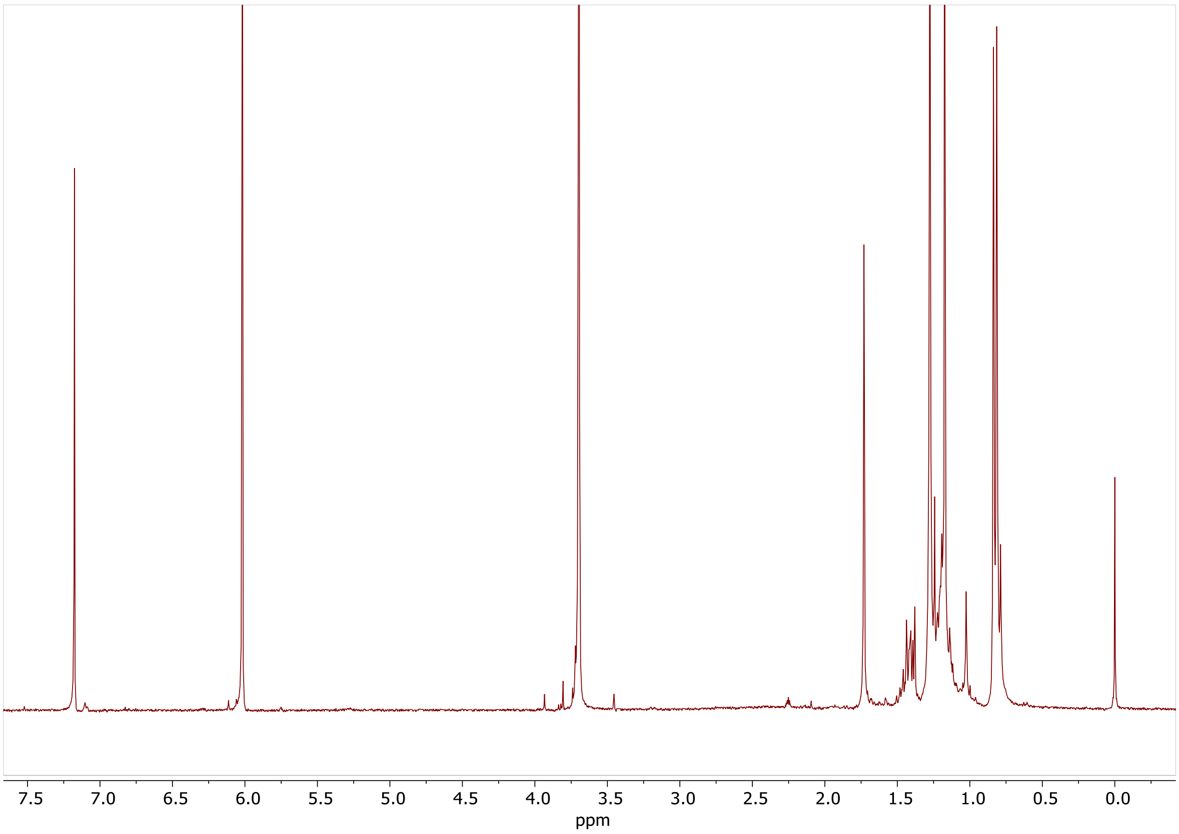

Figure S71: ^{1}H NMR spectrum of crude reaction mixture with **8a** (t = 30 min) in $CDCl_3$ with 1,3,5-Trimethoxybenzene as internal standard.

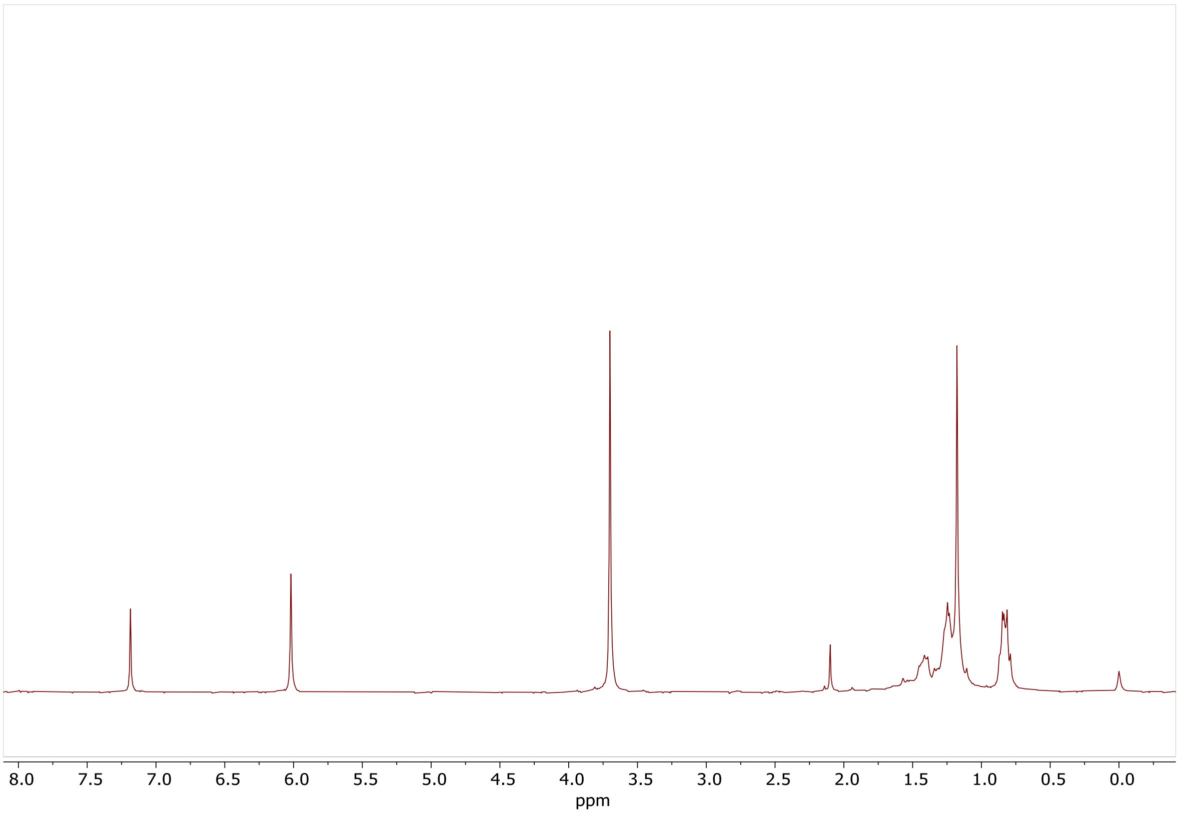

Figure S72: ^{1}H NMR spectrum of crude reaction mixture with **9a** (t = 30 min) in $CDCl_3$ with 1,3,5-Trimethoxybenzene as internal standard.

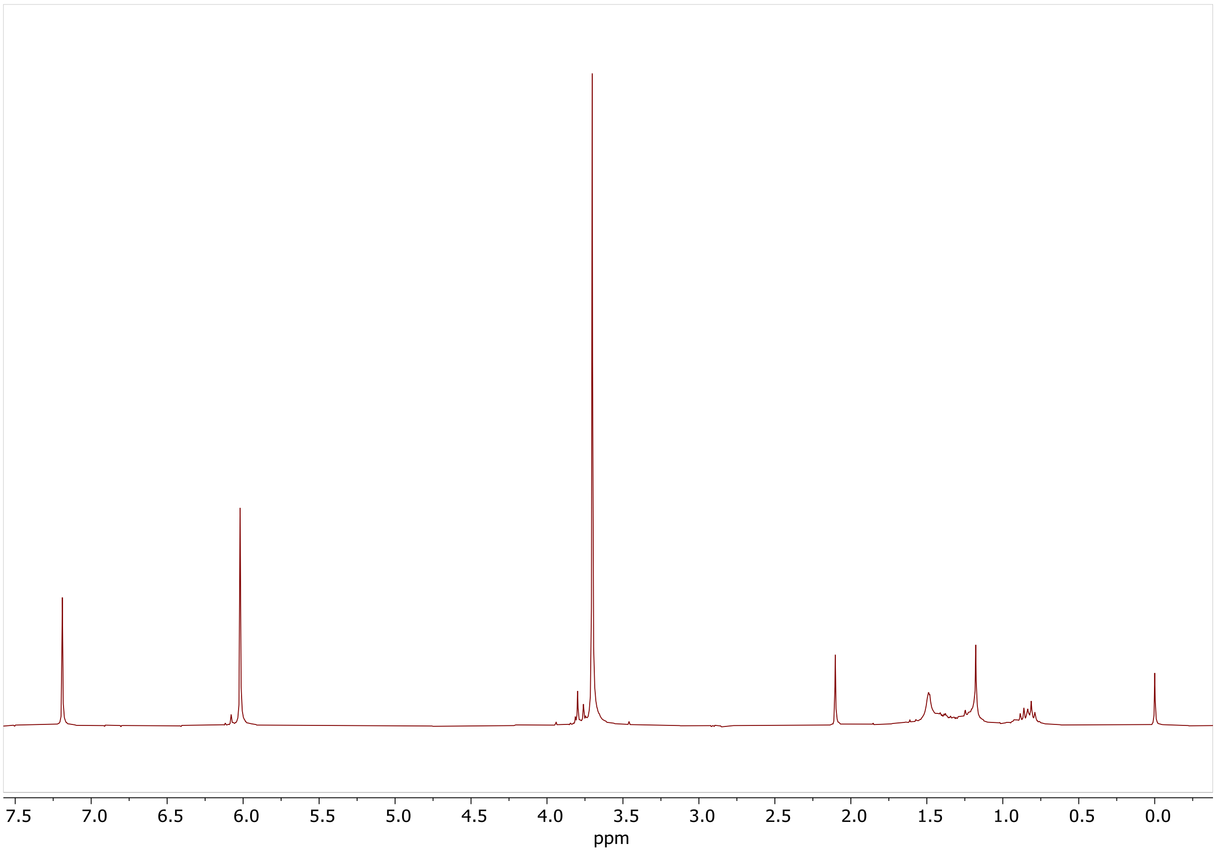

Figure S73: ^{1}H NMR spectrum of crude reaction mixture with **10a** (t = 30 min) in $CDCl_3$ with 1,3,5-Trimethoxybenzene as internal standard.

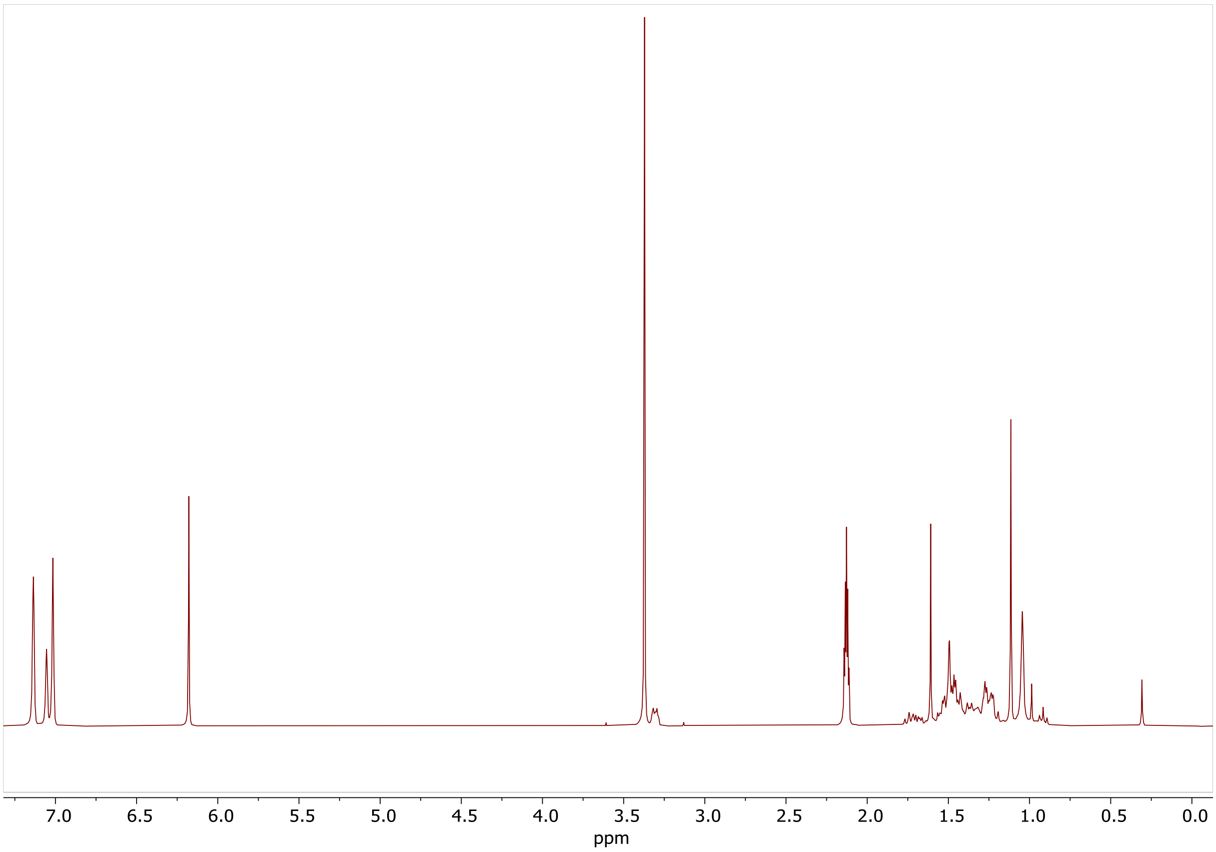

Figure S74: ^{1}H NMR spectrum of crude reaction mixture with **11a** (t = 6 h) in $CDCl_3$ with 1,3,5-Trimethoxybenzene as internal standard.

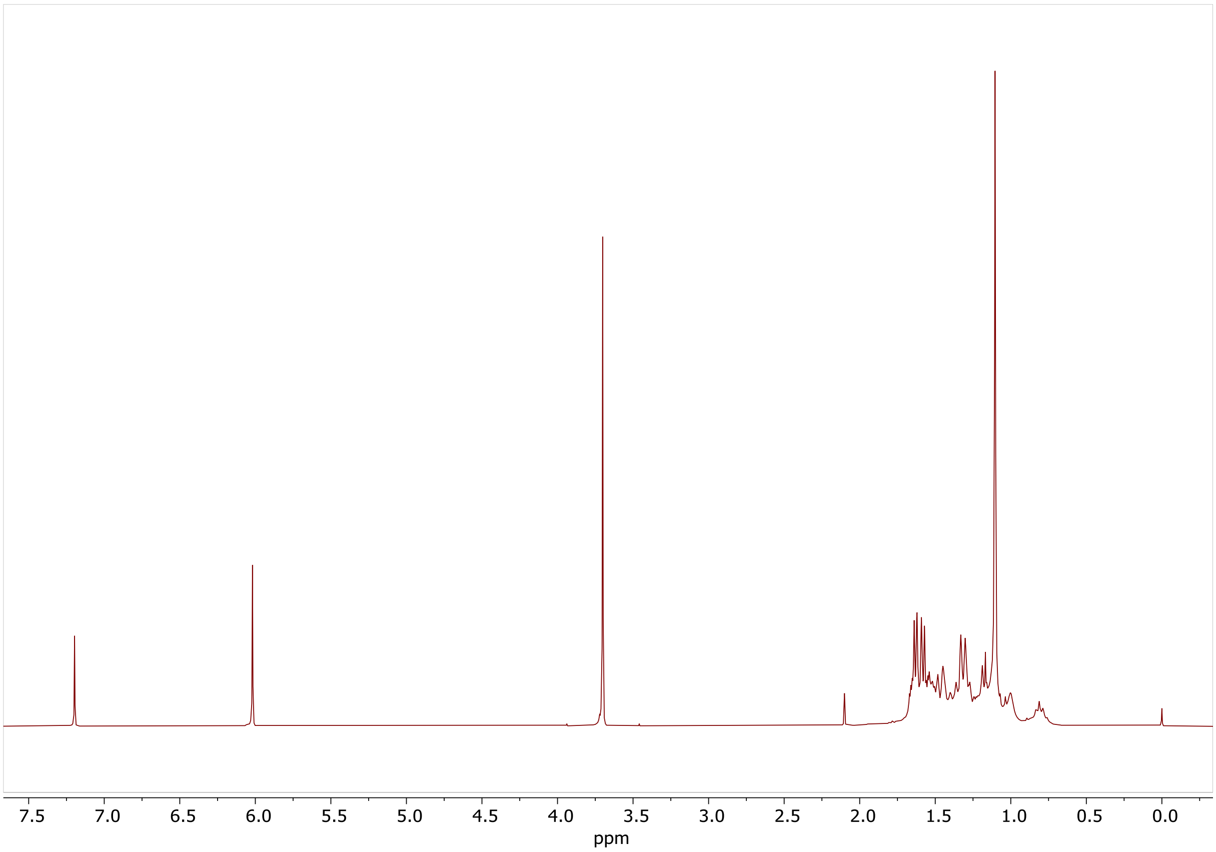

Figure S75: ^{1}H NMR spectrum of crude reaction mixture with **12a** (t = 6 h) in $CDCl_3$ with 1,3,5-Trimethoxybenzene as internal standard.

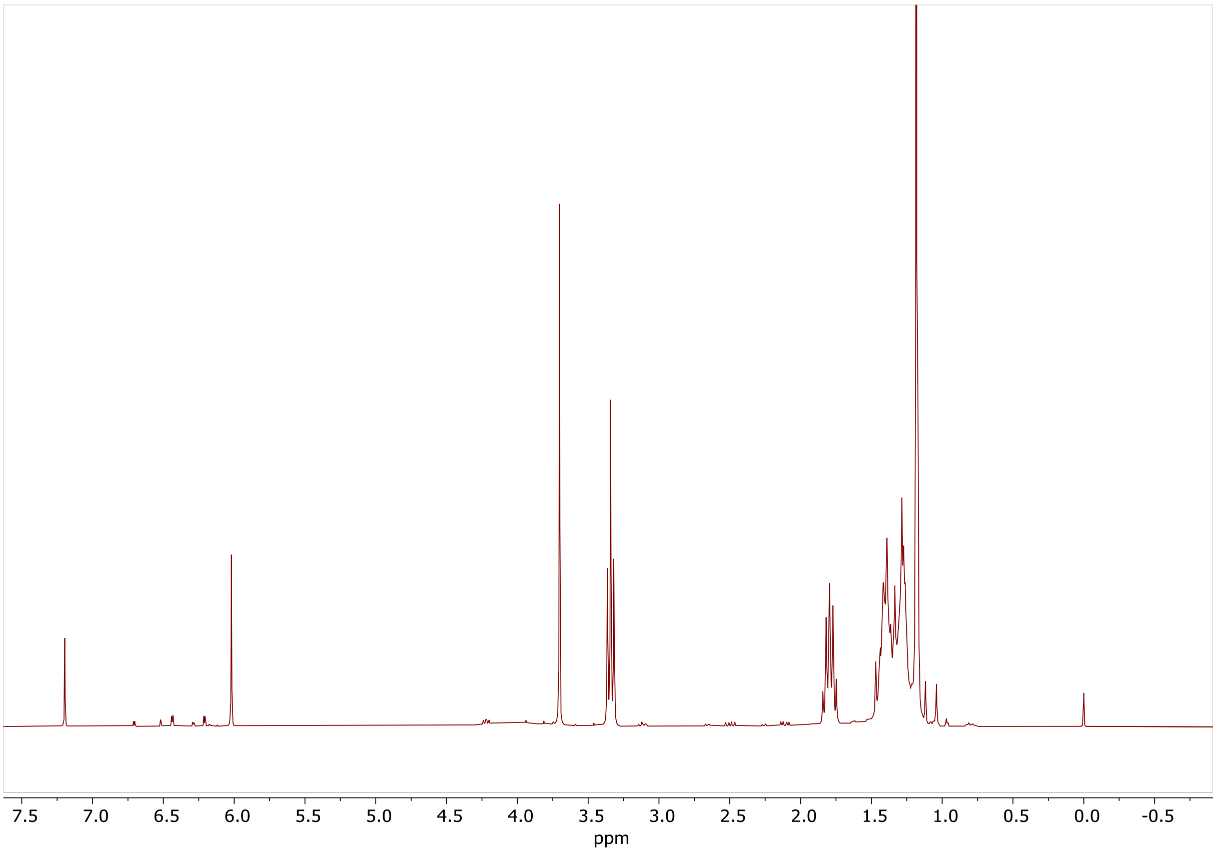

Figure S76: ^{1}H NMR spectrum of crude reaction mixture with **13a** (t = 30 min) in $CDCl_3$ with 1,3,5-Trimethoxybenzene as internal standard.

Crystallographic and refinement data

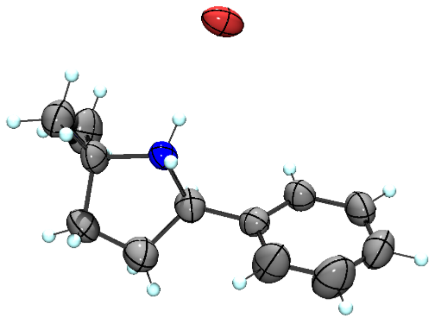

Figure S77: ORTEP representation of protonated amine **1b** (50% probability ellipsoids).

Table S3: Crystal data and structure refinement for WS381.

**Identification code**	21MA168_WS381
**CCDC deposit number**	2208587
**Empirical formula**	$C_{24}H_{53}FeN_3Si_4$
**Formula weight**	551.90
**Temperature/K**	173.01(10)
**Crystal system**	monoclinic
**Space group**	$P2_1/n$
**a/Å**	18.67067(17)
**b/Å**	18.94109(18)
**c/Å**	18.90816(17)

**α/°**	90
**β/°**	94.3084(8)
**γ/°**	90
**Volume/Å^3**	6667.84(11)
**Z**	8
**ρ_{calc}g/cm^3**	1.100
**μ/mm^{-1}**	5.110
**F(000)**	2400.0
**Crystal size/mm^3**	0.258 × 0.226 × 0.083
**Radiation**	Cu Kα (λ = 1.54184)
**2Θ range for data collection/°**	6.416 to 153.478
**Index ranges**	-23 ≤ h ≤ 23, -23 ≤ k ≤ 22, -23 ≤ l ≤ 23
**Reflections collected**	132519
**Independent reflections**	14043 [R_{int} = 0.0583, R_{sigma} = 0.0238]
**Data/restraints/parameters**	14043/90/742
**Goodness-of-fit on F^2**	1.076
**Final R indexes [I>=2σ (I)]**	R_1 = 0.0509, wR_2 = 0.1462
**Final R indexes [all data]**	R_1 = 0.0572, wR_2 = 0.1523
**Largest diff. peak/hole / e Å^{-3}**	0.97/-0.58

References

[S1] Fulmer, G. R.; Miller, A. J. M.; Sherden, N. H.; Gottlieb, H. E.; Nudelman, A.; Stoltz, B. M.; Bercaw, J. E.; Goldberg, K. I. NMR Chemical Shifts of Trace Impurities: Common Laboratory Solvents, Organics, and Gases in Deuterated Solvents Relevant to the Organometallic Chemist. *Organometallics* **2010**, *29*, 2176–2179.

[S2] Oxford Diffraction (2018). CrysAlisPro (Version 1.171.40.37a). Oxford Diffraction Ltd., Yarnton, Oxfordshire, UK.

[S3] Sheldrick, G. M. (2015). Acta Cryst. C71, 3-8.

[S4] Dolomanov, O. V.; Bourhis, L. J.; Gildea, R. J.; Howard, J. A. K.; Puschmann, H. OLEX2: A Complete Structure Solution, Refinement and Analysis Program. *J. Appl. Crystallogr.* **2009**, *42*, 339–341.

[S5] Hennessy, E. T.; Betley, T. A. Complex N-Heterocycle Synthesis via Iron-Catalyzed, Direct C-H Bond Amination. *Science* **2013**, *340*, 591–595.

[S6] Baek, Y.; Betley, T. A. Catalytic C-H Amination Mediated by Dipyrrin Cobalt Imidos. *J. Am. Chem. Soc.* **2019**, *141*, 7797–7806.

[S7] Kuijpers, P. F.; Tiekink, M. J.; Breukelaar, W. B.; Broere, D. L. J.; van Leest, N. P.; van der Vlugt, J. I.; Reek, J. N. H.; de Bruin, B. Cobalt-Porphyrin-Catalysed Intramolecular Ring-Closing C−H Amination of Aliphatic Azides: A Nitrene-Radical Approach to Saturated Heterocycles. *Chem. Eur. J.* **2017**, *23*, 7945–7952.

[S8] Shimogaki, M.; Fujita, M.; Sugimura, T. Metal-Free Enantioselective Oxidative Arylation of Alkenes: Hypervalent-Iodine-Promoted Oxidative C−C Bond Formation. *Angew. Chem., Int. Ed.* **2016**, *55*, 15797–15801.

[S9] Álvarez, S.; Álvarez, R.; Khanwalkar, H.; Germain, P.; Lemaire, G.; Rodríguez-Barrios, F.; Gronemeyer, H.; de Lera, Á. R. Retinoid Receptor Subtype-Selective Modulators through Synthetic Modifications of RARγ Agonists. *Bioorganic Med. Chem.* **2009**, *17*, 4345–4359.

[S10] Dong, Y.; Clarke, R. M.; Porter, G. J.; Betley, T. A. Efficient C-H Amination Catalysis Using Nickel-Dipyrrin Complexes. *J. Am. Chem. Soc.* **2020**, *142*, 10996–11005.

[S11] Li, N. N.; Zhang, Y. L.; Mao, S.; Gao, Y. R.; Guo, D. D.; Wang, Y. Q. Palladium-Catalyzed C-H Homocoupling of Furans and Thiophenes Using Oxygen as the Oxidant. *Org. Lett.* **2014**, *16*, 2732–2735.

[S12] Estévez, M. C.; Galve, R.; Sánchez-Baeza, F.; Marco, M. P. Disulfide Symmetric Dimers as Stable Pre-Hapten Forms for Bioconjugation: A Strategy to Prepare Immunoreagents for the Detection of Sulfophenyl Carboxylate Residues in Environmental Samples. *Chem. Eur. J.* **2008**, *14*, 1906–1917.

[S13] Khalaf, A. A.; Roberts, R. M. Friedel-Crafts Cyclialkylations of Certain Mono- and Diphenylsubstituted Alcohols and Alkyl Chlorides. *J. Org. Chem.* **1972**, *37*, 4227–4235.

[S14] Stroek, W.; Keilwerth, M.; Pividori, D. M.; Meyer, K.; Albrecht, M. An Iron–Mesoionic Carbene Complex for Catalytic Intramolecular C–H Amination Utilizing Organic Azides. *J. Am. Chem. Soc.* **2021**, *143*, 20157–20165.

[S15] Zhao, J.; Zhao, X. J.; Cao, P.; Liu, J. K.; Wu, B. Polycyclic Azetidines and Pyrrolidines via Palladium-Catalyzed Intramolecular Amination of Unactivated C(Sp3)-H Bonds. *Org. Lett.* **2017**, *19*, 4880–4883.

[S16] Łażewska, D.; Mogilski, S.; Hagenow, S.; Kuder, K.; Głuch-Lutwin, M.; Siwek, A.; Więcek, M.; Kaleta, M.; Seibel, U.; Buschauer, A.; Filipek, B.; Stark, H.; Kieć-Kononowicz, K. Alkyl Derivatives of 1,3,5-Triazine as Histamine H 4 Receptor Ligands. *Bioorganic Med. Chem.* **2019**, *27*, 1254–1262.

[S17] Okazawa, N. E.; Sorensen, T. S. Solution Carbocation Stabilities Measured by Internal Competition for a Hydride Ion. *Can. J. Chem.* **1982**, *60*, 2180–2193.

[S18] Lide, D. R. *Handbook of Chemistry and Physics*; CRC Press.
